# Copper Imidazolin-imine Coordination Compounds as Precursors for a Cu/Al Complex

**DOI:** 10.1021/acs.inorgchem.4c02530

**Published:** 2024-09-11

**Authors:** Ivan Antsiburov, Johannes Stephan, Richard J.J. Weininger, Christian Gemel, Roland A. Fischer

**Affiliations:** † Technical University of Munich, School of Natural Sciences, Department of Chemistry, Chair of Inorganic and Metal−Organic Chemistry Lichtenbergstrasse 4, 85748 Garching, Germany; ‡ Technical University of Munich, Catalysis Research Center, Ernst-Otto-Fischer Strasse 1, 85748 Garching, Germany

## Abstract

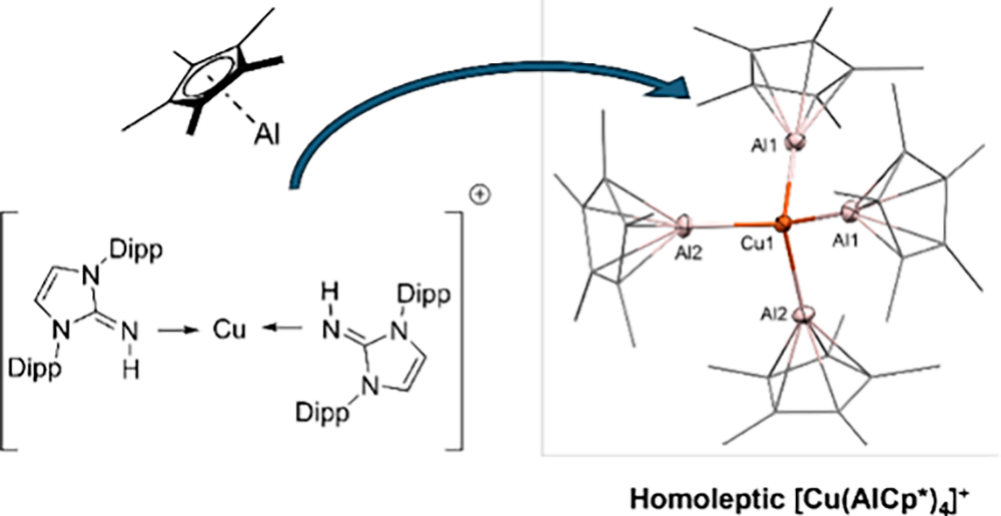

The reactions of [(CF_3_SO_3_Cu)_2_(C_6_H_6_)] with the sterically hindered imidazolin-2-imine ligands DippImTMS (1,3-Bis(2,6-diisopropylphenyl)-2-(trimethylsilylimino)imidazoline) or DippImH (1,3-bis(2,6-diisopropylphenyl) imidazolin-2-imine) lead to the formation of the linear copper(I) complexes [Cu(DippImTMS)(OTf)] (**1**) and [Cu(DippImH)_2_][OTf] (**2**), respectively. The triflate counteranion in **2** can be easily exchanged to the weakly coordinating [BAr^F^] giving [Cu(DippImH)_2_][BAr^F^] (**3**) (BAr^F^ = tetrakis[3,5-bis(trifluoromethyl)phenyl]borate). Substitution of the N-heterocyclic imine (NHI) ligand in **3** by AlCp* (Cp* = pentamethylcyclopentadienyl) gives the tetrahedral [Cu(AlCp*)_4_][BAr^F^] (**5**). The reaction between lithiated imidazolin-2-iminate DippImLi and CuCl results in the triangular cluster [Cu_3_(DippIm)_2_Cl] (**4**). All products have been fully characterized by ^1^H- and ^13^C NMR, mass spectrometry, as well as SC-XRD.

## Abstract 2

Reactions of [(CF_3_SO_3_Cu)_2_(C_6_H_6_)] with sterically hindered imidazolin-2-imine ligands DippImTMS (1,3-Bis(2,6-diisopropylphenyl)-2-(trimethylsilylimino)imidazoline) or DippImH (1,3-bis(2,6-diisopropylphenyl) imidazolin-2-imine) yield linear copper(I) complexes [Cu(DippImTMS)(OTf)] (**1**) and [Cu(DippImH)_2_][OTf] (**2**), respectively. The triflate counteranion in **2** can be easily exchanged to the weakly coordinating [BAr^F^] giving [Cu(DippImH)_2_][BAr^F^] (**3**) (BAr^F^ = tetrakis[3,5-bis(trifluoromethyl)phenyl]borate). N-heterocyclic imine (NHI) ligand substitution in **3** by AlCp* (Cp* = pentamethylcyclopentadienyl) gives the tetrahedral [Cu(AlCp*)_4_][BAr^F^] (**5**). The reaction between lithiated imidazolin-2-iminate DippImLi and CuCl gives the triangular cluster [Cu_3_(DippIm)_2_Cl] (**4**).

## Introduction

Transition metal complexes with carbenoid ligands ER (E = Al, Ga; R = Cp*, C(SiMe_3_), TMP, Ar) have been well investigated due to their diverse reactivities toward small molecules. Key examples include the reactions of the transient species Ni(AlCp*)_3_, related to [Ni(AlCp*)_4_],^1^ and [Ru(GaCp*)_3_H_2_]^2^ which both oxidatively add benzene or toluene under very mild conditions, or the rearrangement of [RhCp*(GaCp*)(CH_3_)_2_] to [RhCp*(C_5_Me_4_Ga(CH_3_)_3_)], which involves the cleavage of a C–C bond proceeding in the solid state below room temperature.^3^ The high reactivity of M(ER)_*n*_ species toward bond activation reactions of substrates XY can be attributed to cooperative effects between the two metals M and E, with the electropositive metal E pushing the thermodynamic driving force of the overall reaction by the formation of strong E–X and E–Y bonds. This is also evident in the very recent example of [Ni_3_(GaTMP)_7_] (TMP = 2,2,6,6-tetramethylpiperidide), which catalyzes the semihydrogenation of internal alkynes to alkenes. One key feature of this cluster is the ability for high H_2_ uptake (up to 3 molecules) while maintaining high catalytic activity, which is mainly provided by the gallium atoms serving as a “hydrogen reservoir”.^4^

The typical method for synthesizing such complexes involves substituting olefin ligands with ER. For instance, [Ni_3_(GaTMP)_7_] can be conveniently synthesized by the reaction of Ni(cod)_2_ with GaTMP, resulting in a complete substitution of the cod ligands (cod = 1,5-cyclooctadiene).^4^ Similarly, Pt(cod)_2_ or Pd_2_(dvds)_3_ (dvds = tetramethyl-divinyldisiloxane) react with ECp* (E = Al, Ga, In) to form closed shell complexes or clusters such as [M(ECp*)_4_], [M_2_(ECp*)_5_], or [M_3_(ECp*)_8_].^5,6^ This substitution process can also be supported by hydrogenolytic conditions, as demonstrated in the synthesis of [Mo(GaCp*)_6_] from [Mo(η^4^-C_4_H_6_)_3_] in the presence of H_2_.^7^

However, while alkene complexes as precursors are highly amenable to substitution by ER ligands, phosphine and carbonyl-containing complexes exhibit a similar yet distinctly weaker reactivity, often resulting in incomplete substitution. For example, [Ni(PEt_3_)_4_] undergoes stepwise substitution of PEt_3_ by AlCp*, yielding [Ni(PEt_3_)_4-a_(AlCp*)_*a*_] (*a* = 1, 2).^8^ Substitution of CO is even more challenging due to its strong π-acceptor properties, i.e., stepwise CO/ER_3_ exchange increases the binding of the remaining CO ligands to the M center. Thus, the above-mentioned [Mo(GaCp*)_6_] cannot be obtained from [Mo(CO)_6_], and treatment of [*fac*-(MeCN)_3_Mo(CO)_3_] with excess of GaCp* leads to [*fac*-(Cp*Ga)_3_Mo(CO)_3_] only.^9^ Also monodentate GaR ligands bearing a bulky terphenyl-derived group are known to stabilize transition metal centers.^10−12^ The same holds for targeting homoleptic clusters [M_*a*_(ER)_*b*_], i.e., the reaction of Rh_6_(CO)_16_ with GaCp* produces several substitution products, with [Rh_6_(CO)_12_(GaCp*)_4_] as the cluster with the highest degree of substitution.^13^

Examples using homoleptic precursors with nitrogen-based donor ligands such as CH_3_CN are mainly limited to the displacement of the nitrile ligands. A prominent illustration is the reaction of [Cu(MeCN)_4_][BAr^F^] with GaCp*, yielding [Cu(GaCp*)_4_][BAr^F^].^14^ The cation is isoelectronic to the well-known group 10 [M(ECp*)_4_] (E = Al, Ga) and is a typical 18 valence electron complex with a tetrahedral geometry. Due to the oxidation state of Cu(I) however, the redox properties of the ECp* ligand must be taken into account, especially in the case of Al(I) ligands.

Synthesizing transition metal complexes with AlCp* ligands is often quite challenging. The low solubility of AlCp* in common organic solvents at room temperature prevents the synthesis of these complexes under mild conditions, necessitating elevated temperatures, while the resulting complexes are often highly reactive. For example, C–H activation of solvents or the Cp* ligands may occur, such as in Ni(AlCp*)_3_ or M(AlCp*)_5_ (M = Fe, Ru), respectively.^1,15^ Another common issue is the transfer of Cp* to the transition metal, which occurs for example in the reaction of [FeBr_2_(L)_2_] with AlCp*.^16^

Aiming for expanding the precursor library for the access to homoleptic M/E complexes and building blocks for related clusters, we were thus led to investigate the chemistry of imidazolin-2-imine and imidazolin-2-iminato (NHI) ligated complexes as starting compounds for our target systems. NHI ligands have been extensively studied, particularly as ancillary ligands in a variety of homogeneous catalysts, e.g., for olefin polymerization and alkyne metathesis.^17−20^ Imidazolin-2-iminato ligands are pseudoisolobal to cyclopentadienyl ligands and exhibit efficient and tunable electron-donating and steric properties.^17,21,22^ They can form very robust metal–nitrogen bonds, especially when coordinating to highly electrophilic metal centers such as Ti(IV),^20^ V(V),^23^ and Mo(VI).^24^ However, complexes of electron-rich late transition metals featuring these ligands have received significantly less attention.^25−27^ In this study, we investigate the capability of NHI ligands to act as effective leaving groups in copper complexes, facilitating the synthesis of a new AlCp* complex of copper(I) while demonstrating tolerance against the strongly reducing nature of AlCp*.

## Results and Discussion

### Cu-NHI Complexes

**Scheme 1:**
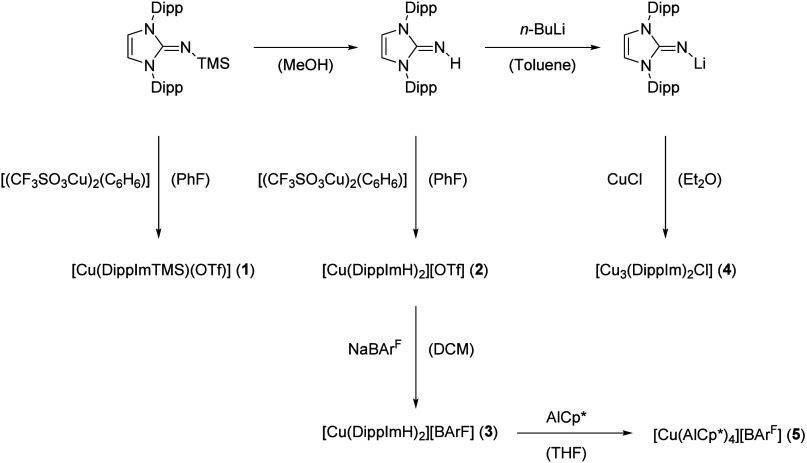
Reaction scheme of Cu and Cu/Al complexes **1** - **5**, studied within the scope of this work

**Figure 1:**
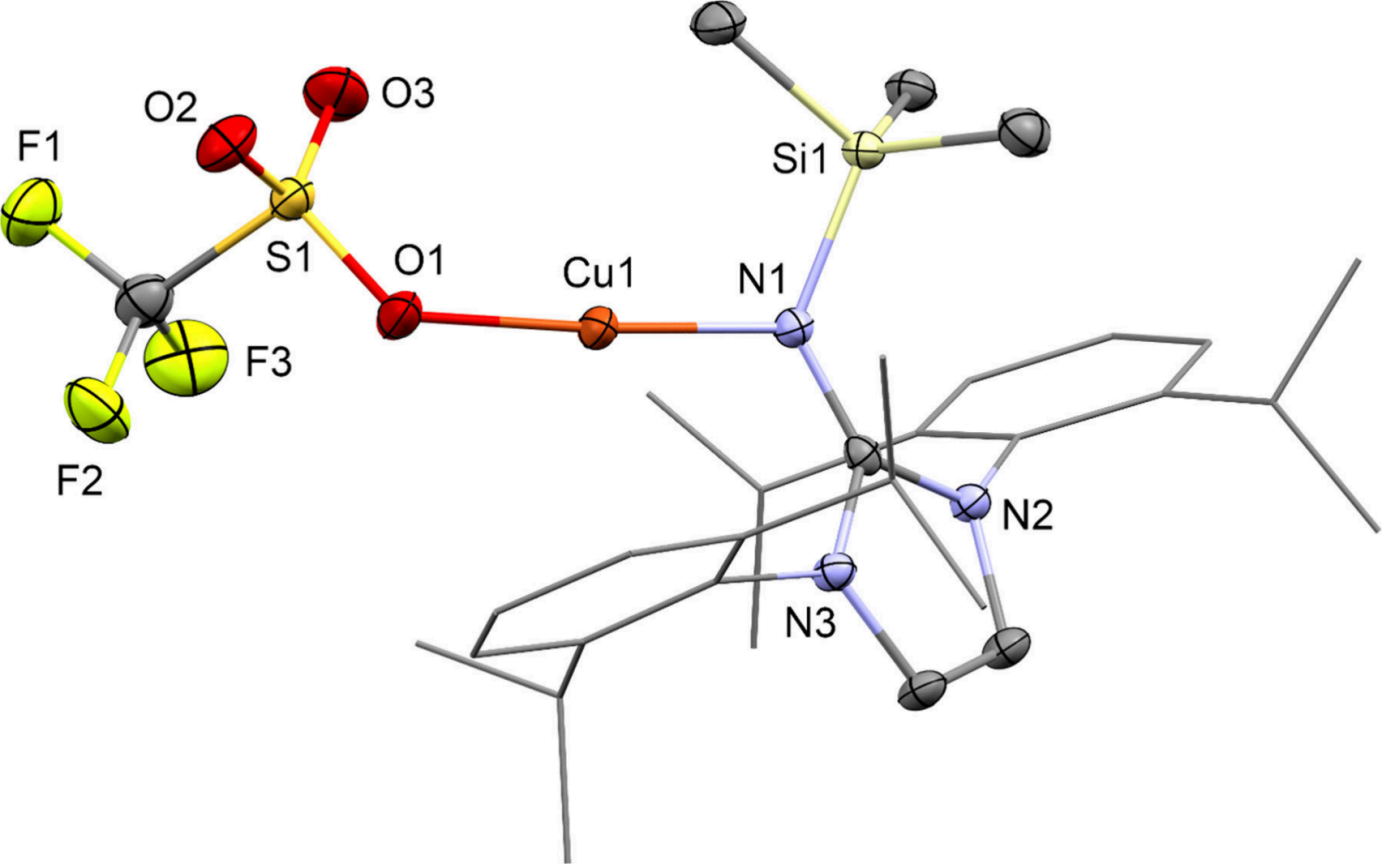
Molecular structure of [Cu(DippImTMS)(OTf)] (**1**) in the solid state as determined by SC-XRD. Cu: orange, Si: beige, S: yellow, F: green-yellow, O: red, N: blue, and C: gray. H-atoms and cocrystallized solvent molecules are omitted for clarity. Thermal ellipsoids are shown at the 50% probability level. Relevant bond distances and angles and details of the crystallographic data are given in the Supporting Information.

The reaction of [(CF_3_SO_3_Cu)_2_(C_6_H_6_)] with one equivalent (per copper) of 1,3-bis(2,6-diisopropylphenyl)-2-(trimethylsilylimino)imidazoline (DippImTMS) in fluorobenzene as the solvent gives [Cu(DippImTMS)(OTf)] (**1**) in excellent yields (Scheme 1). Complex **1** is well soluble in fluorobenzene, THF, or hot toluene and is stable under inert gas. Single crystals of **1** were obtained from cold fluorobenzene. Compound **1** crystallize in the space group *P*2_1_/*n*. As determined by X-ray crystallography, **1** is a monomeric copper complex with almost linear N–Cu–O coordination (angle 174.93°) and short Cu–N (1.884 Å) and Cu–O (1.878 Å) distances (compared to [IPrCuCl] (IPr = 1,3-Bis(2,6-diisopropylphenyl)imidazol-2-ylidene) where C–Cu is 1.953 Å)^28^ (Figure 1). Considering the sum of van-der-Waals radii of Cu with C or N, this bond appears remarkably short. This indicates a strong chemical bond between the electrophilic copper center and the σ- and π-donating NHI-Ligand. The Cu–N distance in **1** of 1.884 Å is also significantly shorter than the value of 2.002 Å found in the closely related complex [(BL^iPr^)CuCl] (BL = ethylene-bridged bis(imidazolin-2-imine)).^29^ This latter complex, however, features a chelating bis-NHI ligand and thus a tricoordinate Cu center, which explains the longer Cu–N distances. The Cu–O distance in **1** is shorter than in [(CuOTf)_2_(C_6_H_6_)] (2.00–2.22 Å),^30^ which could be explained by tetrahedral coordination of copper in [(CF_3_SO_3_Cu)_2_(C_6_H_6_)] vs linear in **1**.

In general, Cu(I) complexes in nitrogen ligand environments exhibit a variety of coordination geometries, depending on the chosen ligands. While linear geometry is very common with heterocyclic ligands such as imidazole, pyrazole, or pyridine,^31,32^ trigonal planar cooridination is well-documented for pincer-type ligands.^33^ Tetrahedral coordination is frequently observed with small ligands like MeCN or tmeda.^34^ The structural motif for copper(I) amides is dominated by cyclic oligomeric structures.^35^

**Figure 2:**
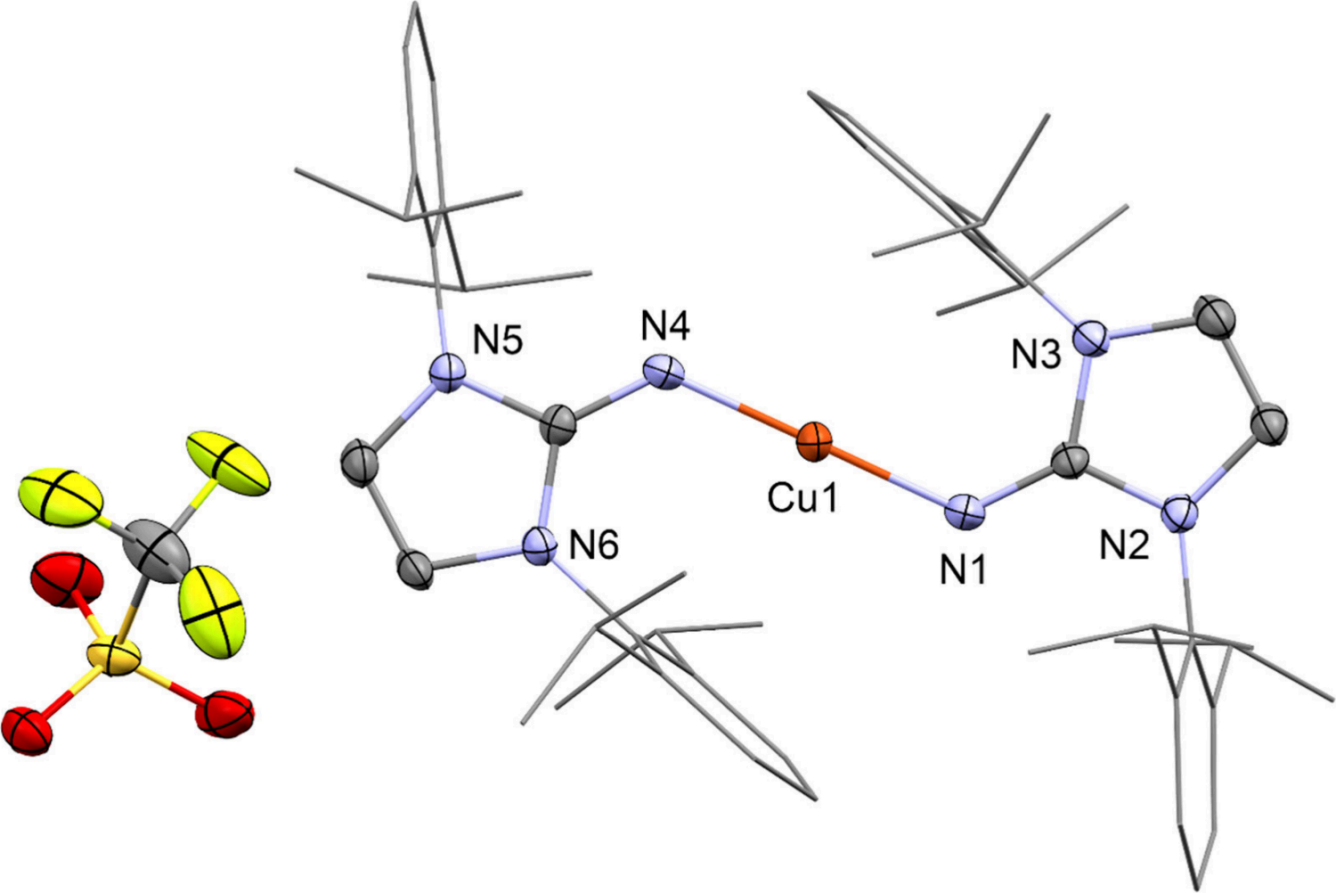
Molecular structure of [Cu(DippImH)_2_][OTf] (**2**) in the solid state as determined by SC-XRD. Cu: orange, S: yellow, F: green-yellow, O: red, N: blue, and C: gray. H-atoms and cocrystallized solvent molecules are omitted for clarity. Thermal ellipsoids are shown at the 50% probability level. Relevant bond distances and angles and details of the crystallographic data are given in the Supporting Information.

The reaction of [(CF_3_SO_3_Cu)_2_(C_6_H_6_)] with two equivalents of 1,3-bis(2,6-diisopropylphenyl)-imidazoline-2-imine per copper in fluorobenzene leads to [Cu(DippImH)_2_][OTf] (**2**) in very good yield (Scheme 1). This air stable complex is highly soluble only in polar organic solvents such as CHCl_3_, CH_2_Cl_2_, or THF. Compound **2** crystallize in the chiral space group *P*2_1_. The molecular structure of the cation was determined by single crystal X-ray diffraction and shows the copper atom in the center coordinated by two ligands in an almost linear fashion (N–Cu–N angle 179.83°) (Figure 2). Both ligands are almost coplanar with an angle between the two plains containing ligands of 1.81° (see Figure S32). The Cu–N distance (1.842 Å) is shorter than for typical copper amides, for example [CuN(SiMe_3_)_2_]_4_ (1.921 Å),^36^ although with a different geometry. Additionally, the average Cu–N distance is slightly shorter than the Cu–N distance in **1**, despite DippImTMS expected to be a better donor than DippImH. This deviation can be explained by the absence of a direct bond to the anion in **2** as compared to **1**. In the unit cell, the triflate anion of **2** is not coordinated to the cation [Cu(DippImH)_2_]^+^ with the shortest Cu–O distance being 7.327 Å.

The triflate anion in **2** can be replaced by the sterically demanding and weakly coordinating BAr^F^ anion (Scheme 1). Reaction of **2** with NaBAr^F^ in dichloromethane gives [Cu(DippImH)_2_][BAr^F^] (**3**) in respectable isolated yields. As expected, the cation of **3** in solution as well as in the solid state is identical to that in **2**, as shown by NMR and SC-XRD analysis (see Table S3).

The reaction between CuCl and bis(2,6-diisopropylphenyl)imidazolin-2-imino-lithium (DippImLi) in diethyl ether yielded a mixture of products, from which [Cu_3_(DippIm)_2_Cl] (**4**) could be isolated in small quantities through fractional crystallization (Scheme 1). Despite numerous attempts, no other product from the mixture could be separated and characterized with satisfying accuracy. Single crystals of **4** suitable for single-crystal X-ray diffraction (SC-XRD) were obtained from cold hexane. The compound **4** crystallizes in the space group *C*2/*c*.

**Figure 3:**
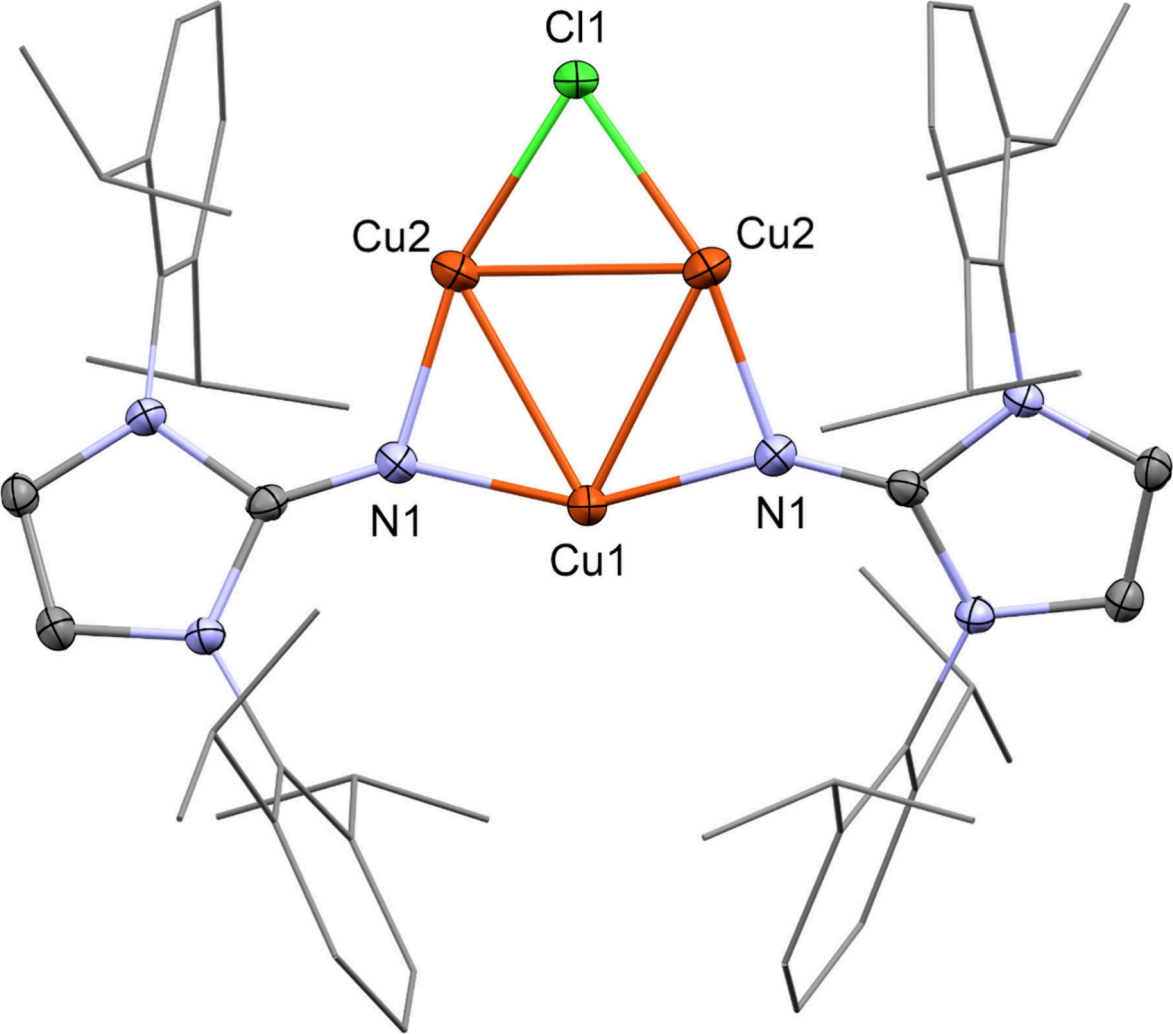
Molecular structure of [Cu_3_(DippIm)_2_Cl] (**4**) in the solid state as determined by SC-XRD. Cu: orange, Cl: green, N: blue, and C: gray. H-atoms and cocrystallized solvent molecules are omitted for clarity. Thermal ellipsoids are shown at the 50% probability level. Relevant bond distances and angles and details of the crystallographic data are given in the Supporting Information.

In the molecular structure of **4**, three copper atoms form a triangle with two imidazolin-2-iminato ligands and one chlorido ligand bridging the three Cu–Cu edges of the Cu_3_ triangle (Figure 3). The copper triangle displays slight distortion, with Cu–Cu distances of 2.522 Å for the edges bridged by NHI ligands (with an average copper–nitrogen distance of 1.847 Å), whereas the chlorido-bridged Cu–Cu bond measures 2.470 Å, notably shorter. The copper–copper distances in **4** are close to those (2.402 Å) in structural-related [Cu(Si(TMS))_3_]_3_ or for other Cu(I)–Cu(I) bonds (such as Cu_5_Mes_5_ with an average Cu(I)–Cu(I) bond of 2.447 Å).^37,38^ The copper–nitrogen distances in **4** are comparable to those in [CuN(SiMe_3_)_2_]_4_ (1.921 Å).^36^ Additionally, the copper–copper distances in **4** are well within the range (below 2.8 Å) of intramolecular ligand-supported cuprophilic interactions.^39^

Unfortunately, all attempts to more selectively synthesize and isolate compound **4** in its pure form on a preparative scale proved unsuccessful, as evidenced by insufficient copper content revealed in elemental analysis. Signals observed around 0 ppm in ^7^Li NMR may suggest the presence of organo-lithium species or anionic Cu clusters (Figure S13). Diffusion-ordered spectroscopy (DOSY) NMR analysis indicates the presence of several species bearing NHI groups with similar diffusion coefficients (Figure S14), some of which could represent further oligomers like [Cu_4_(DippIm)_2_Cl_2_], as detected in an LIFDI mass spectrum (Figure S31).

### [Cu(AlCp*)_4_][BAr^F^]

Treating [Cu(DippImH)_2_][BAr^F^] (**3**) with four equivalents of AlCp* in boiling THF results in the formation of [Cu(AlCp*)_4_][BAr^F^] (**5**) featuring the homoleptic CuAl_4_ complex cation (Scheme 1). In the ^1^H NMR spectrum of [Cu(AlCp*)_4_][BAr^F^] (**5**) in THF-*d*^8^, one signal is observed for all chemically equivalent AlCp* ligands at 1.95 ppm, alongside the aromatic signals of the anion (Figure S15). The ^13^C NMR spectrum of **5** shows no unusual features (Figure S16). The ^27^Al-NMR spectrum of **5** shows, as expected, only one broad signal at −57.74 ppm (Figure S18). Unfortunately, no [Cu(AlCp*)_4_]^+^ could be observed neither with ESI- nor with LIFDI-MS. This selective ligand exchange reaction of NHI with AlCp* is noteworthy due to the strong reducing capabilities of AlCp*, which did not induce reduction of Cu(I) to Cu(0). In comparison, the reaction of [Cu(cod)_2_][BAr^F^] with AlCp* in fluorobenzene at room temperature results in the darkening of solution and precipitation of metallic copper. This could be explained by stronger redox capability of AlCp* than GaCp*, leading to the fast reduction of Cu(I) instead of ligand exchange. After filtration and solvent removal *in vacuo*, the remaining solid was washed with pentane and dissolved in THF-*d*_8_. The ^1^H NMR spectrum shows unidentifiable decomposition products (Figure S21). According to *in situ* NMR-analysis, the formation of **5** is in principle possible starting from [Cu(MeCN)_4_][BAr^F^], as well. However, darkening of the reaction mixture and significant amount of copper precipitate was observed instead (Figures S19 and S20), similar to the reaction of [Cu(cod)_2_][BAr^F^] with AlCp*. These findings show the advantage of the NHI-ligand for the selective synthesis of **5**.

**Figure 4:**
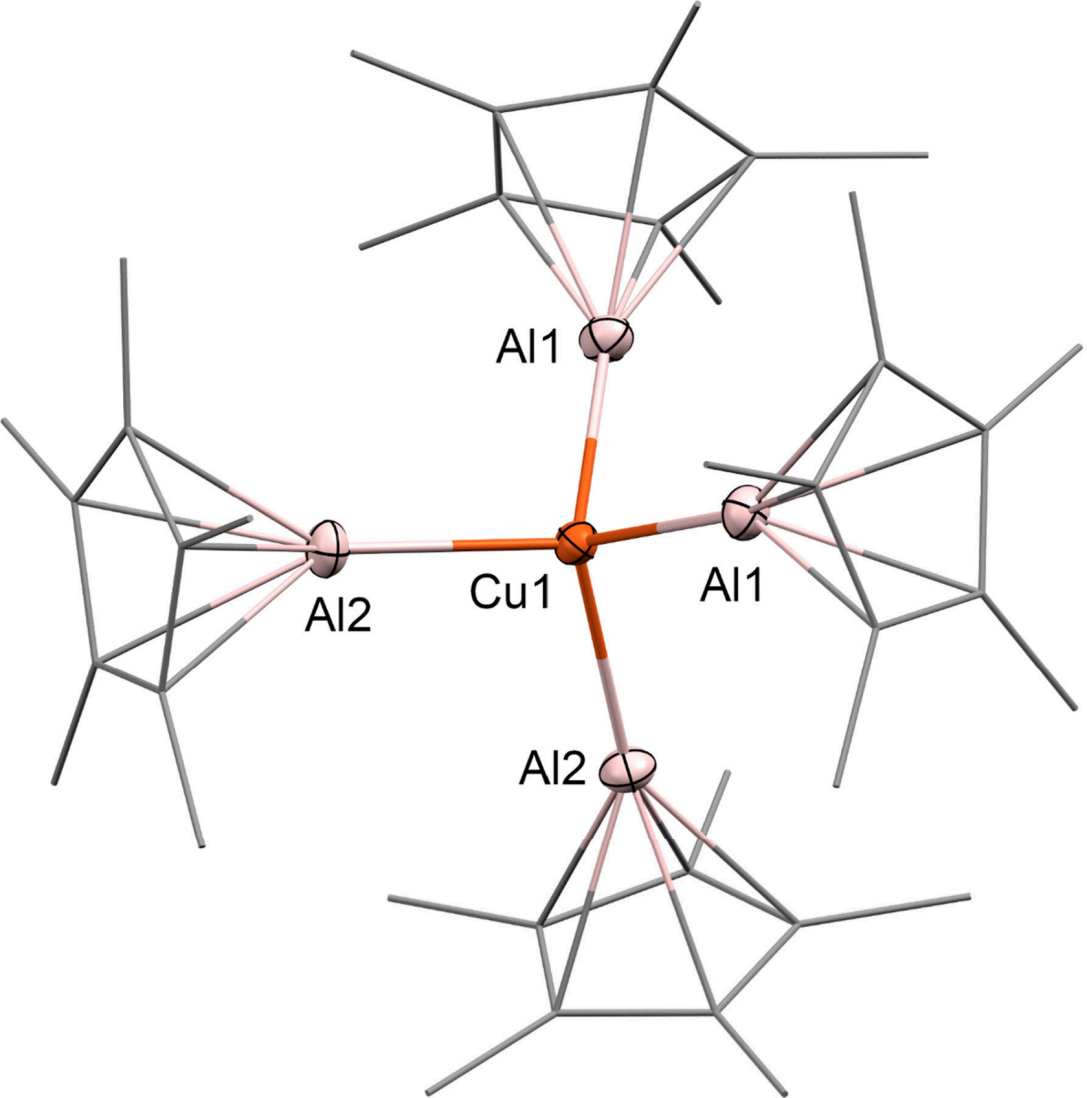
Molecular structure of the cation [Cu(AlCp*)_4_]^+^ of [Cu(AlCp*)_4_][BAr^F^] in the solid state as determined by SC-XRD. Cu: orange, Al: pink, and C: gray. H-atoms and BAr^F^ anion are omitted for clarity. Thermal ellipsoids are shown at the 50% probability level. Relevant bond distances and angles and details of the crystallographic data are given in the Supporting Information.

Single crystals suitable for X-ray diffraction (XRD) were obtained by layering a THF solution of **5** with *n*-hexane. Compound **5** crystallizes in the space group *P*2/*n*. The copper center is coordinated by four AlCp* ligands, arranged in an almost perfect tetrahedron (average Al–Cu–Al angle 109.48°) (Figure 4). The Cu–Al bonds are equidistant with 2.266 Å, which is slightly shorter than the Cu–Ga distances in the homologous compound [Cu(GaCp*)_4_][BAr^F^] (average Cu–Ga of 2.351 Å).^11^ The Cu–Al distance in **5** is also slightly shorter than in the triangular cluster [(Cp*Cu)_2_(μ^2^-AlCp*)] (average Cu–Al of 2.357 Å) or in the complex [(BDI^Mes^)CuAl(BDI^Dip^)] (Cu–Al of 2.301 Å).^40,41^ Interestingly, the Cu–Al distances in **5** is longer than the Ni–Al distances in the isoelectronic [Ni(AlCp*)_4_] (2.173 Å), however the Cp*_centroid_-Al in **5** amount to an average 1.858 Å and are shorter than in [Ni(AlCp*)_4_] (1.933 Å), presumably indicating the more electrophilic properties of the Cu acceptor center in the cation as compared to the neutral Ni complex.^1^ The M-Cp*_centroid_ distances are almost equivalent (4.124 Å for [Cu(AlCp*)_4_]^+^ and 4.106 Å for [Ni(AlCp*)_4_]).

## Conclusion

Two novel linear coordinated copper(I) complexes bearing sterically hindered monodentate imidazolin-2-imine ligands [Cu(DippImTMS)(OTf)] (**1**) and [Cu(DippImH)_2_][OTf] (**2**) were synthesized and fully characterized within the scope of this work. Both complexes feature very short copper ligand distances, but only **2** is air stable. The anion in **2** can be easily exchanged with NaBAr^F^ giving the air stable [Cu(DippImH)_2_][BAr^F^] (**3**). Complex **3**, in contrast to related and well-known [Cu(MeCN)_4_][BAr^F^] or [Cu(cod)_2_][BAr^F^], is suitable for selectively substituting the ligand with AlCp* without triggering a reduction of Cu(I) to Cu(0). The resulting tetrahedral [Cu(AlCp*)_4_][BAr^F^] (**5**) was fully characterized, and its molecular structure was expectedly very similar to its isoelectronic [Ni(AlCp*)_4_] congener. An attempt to prepare copper(I) complexes bearing anionic imidazolin-2-iminato ligands leads to a product mixture, from which the triangular cluster [Cu_3_(DippIm)_2_Cl] (**4**) was isolated, which could be characterized by SC-XRD. Altogether our results suggest the value of NHI ligands as redox-innocent and/or redox-mediating leaving groups for the synthesis of late transition metal [M_*a*_(ER)_*b*_] complexes. We are particularly aiming to explore the properties of the CuAl_4_ containing unit **5** as a building block and the reaction of the NHI-ligated Cu precursors **1**–**4** with AlCp* or alternative Al-sources to expand our library of Cu/Al clusters.^40,42,43^

## Experimental Section

### General

Unless stated otherwise, all synthetic manipulations were carried out using standard Schlenk techniques under an atmosphere of argon 4.6 purified by a BTC-catalyst and dried over 3 Å molecular sieves or in a glovebox under an atmosphere of purified argon. All reactions were carried out in standardized *Schlenk* flasks and tubes which were rinsed with 1,1,1,3,3,3-hexamethyldisilyzane (HMDS), heated with a heat gun to 650 °C, and allowed to cool under vacuum. THF, Et_2_O, toluene, DCM, hexane, pentane, and acetonitrile were purified with solvent purification system (SPS) MB SPS-800 and dried over 3 Å molecular sieves. Benzene and fluorobenzene were distilled under argon and dried over 3 Å molecular sieves. Deuterated solvents were degassed prior to their use and dried over carefully activated 3 Å molecular sieves. The precursor compound [(CF_3_SO_3_Cu)_2_(C_6_H_6_)] was obtained from TCI and recrystallized from boiling benzene. All further reagents were purchased from commercial sources and used as such without further purification. Sodium tetrakis(3,5-bis(trifluoromethyl)phenyl)borate, NaBAr^F^, was prepared according to the literature procedure.^44−46^ 1,3-Bis(2,6-diisopropylphenyl)-2-(trimethylsilylimino)imidazoline, DippImTMS, was prepared according to the literature procedure and distilled prior to use (3 × 10^–3^ mbar, 180 °C).^20^ 1,3-Bis(2,6-diisopropylphenyl)-imidazoline-2-imine, DippImH, and bis(2,6-diisopropylphenyl)imidazolin-2-imino lithium, DippImLi, were prepared according to literature-known methods.^47,48^ Pentamethylcyclopentadienylaluminum(I), AlCp*, was prepared according to a literature procedure.^49^ Caution! All chemicals should be handled with care, and personal protective equipment must be used.

### Analytical Methods

NMR spectra were recorded on a Bruker Avance III 400US (^1^H, 400 MHz; ^13^C 101 MHz, ^27^Al 104 MHz), Bruker AVDH500cr (^1^H, 500 MHz; ^13^C 126 MHz), and Bruker AVHD400 (^1^H, 400 MHz; ^13^C 101 MHz, ^19^F 376 MHz, ^29^Si 79 MHz). Chemical shifts are given relative to TMS for ^1^H, ^13^C, and ^29^Si; CFCl_3_ for ^19^F; Al(NO_3_)_3_ for ^27^Al; and were referenced to the residual solvent peak as internal standards. Chemical shifts are reported in parts per million, downfield shifted from TMS, and are consecutively reported as position (δH or δC), relative integral, multiplicity (s = singlet, d = doublet, q = quartet, sept = septet and m = multiplet), and assignment. FT-IR spectra were measured on an ATR setup with a Bruker Alpha FTIR spectrometer under an inert gas atmosphere and handled within a glovebox. The mass spectra were taken using a Linden CMS LIFDI and ESI as ionization sources and a ThermoFisher Scientific Exactive Plus Orbitrap as the detector. The sample application was performed via a fumed silica capillary from a glovebox under an argon atmosphere to enable the measurement of highly air-sensitive compounds.^50^ The recorded mass spectra were evaluated using the FreeStyle 1.3 program from ThermoFisher Scientific.

#### Elemental Analysis (EA)

EA measurements were conducted in the Microanalytical Laboratory at Technical University Munich.

## Experimental Procedures

### Synthesis of [Cu(DippImTMS)][OTf] (**1**)

A Schlenk tube was charged with [(CF_3_SO_3_Cu)_2_(C_6_H_6_)] (1.60 g, 3.18 mmol, 1.0 equiv) and 1,3-bis(2,6-diisopropylphenyl)-2-(trimethylsilylimino)imidazoline, DippImTMS, (3.10 g, 6.52 mmol, 2.05 equiv). Then fluorobenzene (100 mL) was added and the starting materials quickly dissolved. After 4 h at r.t., the resulting slightly yellowish solution was filtered and concentrated to 30 mL. Addition of hexane (50 mL) gives a white precipitate, which was filtered off, washed with hexane (3 × 20 mL), and dried *in vacuo* giving the product as a white solid (4.09 g, 5.94 mmol, 93%). This complex is air sensitive.

^1^H NMR (400 MHz, benzene-*d*_6_): δ 7.34 (t, ^3^*J* = 7.8 Hz, 2H, *p*-Ar*H*), 7.10 (d, ^3^*J* = 7.8 Hz, 4H, *m*-Ar*H*), 5.80 (s, 2H, NC*H*), 2.69 (hept, ^3^*J* = 6.8 Hz, 4H, C*H*), 1.30 (d, ^3^*J* = 6.8 Hz, 12H, C*H*_3_), 0.94 (d, ^3^*J* = 6.8 Hz, 12H, C*H*_3_), −0.07 (s, 9H, SiMe_3_).

^13^C NMR (101 MHz, benzene-*d*_6_): δ 152.89 (N*C*N), 146.62 (*o-C*), 132.18 *(p*-*C*H), 130.74 (*ipso-C*), 126.01 (*m*-*C*H), 116.57 (N*C*H), 29.12 (*C*H), 24.92 (CH_3_), 23.05 (*C*H_3_), 3.66 (Si*C*H_3_).

^29^Si NMR (79 MHz, benzene-*d*_6_): δ 0.01.

^19^F-NMR (376 MHz, benzene-*d*_6_): δ −76.99.

IR (ATR, 298 K): ν [cm^–1^] = 2962 (m), 2933 (w), 2872 (w), 1588 (w), 1535 (s), 1463 (m), 1421 (w), 1386 (w), 1366 (w), 1322 (s), 1269 (w), 1248 (w), 1234 (s), 1205 (s), 1176 (s), 1131 (w), 1090 (w), 1059 (w), 1049 (w), 1024 (s), 1016 (s), 933 (m), 904 (s), 832 (s), 801 (m), 756 (s), 731 (w), 713 (w), 692 (m), 630 (s), 571 (w), 515 (m), 443 (w), 433 (w), 414 (w).

ESI-MS: *m*/*z* = 539.26 [Cu(DippImTMS)]^+^; 476.75 (DippImTMS + H)^+^; 870.71 [Cu(DippImH)_2_]^+^; 404.58 (DippImH + H)^+^; 150.70 (OTf)^−^.

Anal. Calcd. C_31_H_45_CuF_3_N_3_O_3_SSi: C, 54.09; H, 6.59; N, 6.10; S, 4.66. Found: C, 54.52; H, 6.88; N, 6.17; S, 4.17.

### Synthesis of [Cu(DippImH)_2_][OTf] (**2**)

A Schlenk tube was charged with [(CF_3_SO_3_Cu)_2_(C_6_H_6_)] (226 mg, 0.45 mmol, 1.0 equiv) and 1,3-bis(2,6-diisopropylphenyl)-imidazoline-2-imine, DippImH (725 mg, 1.80 mmol, 4.0 equiv). Then fluorobenzene (25 mL) was added, and the resulting white suspension was allowed to stir for 24 h. Addition of hexane (30 mL) gives a white precipitate, which was filtered off, washed with hexane (3 × 10 mL), and dried *in vacuo* giving the product as a white solid (812 mg, 0.80 mmol, 89%). This complex is air stable and not hygroscopic.

^1^H NMR (500 MHz, methylene chloride-*d*_2_): δ 7.67 (s, 2H, *p*-Ar*H*), 7.45 (s, 4H, *m*-Ar*H*), 6.93 (s, 4H, *m*-Ar*H*), 6.66 (s, 2H, *p*-Ar*H*), 6.54 (s, 4H, NC*H*), 2.54 (bs, 8H, C*H*), 2.20 (s, 2H, N*H*), 1.38–0.90 (m, 48H, C*H*_3_).

^13^C NMR (126 MHz, methylene chloride-*d*_2_): δ 154.08 (N*C*N), 153.79 (*ipso-C*), 147.99 (*o-C*), 131.70 (*p-C*), 131.01(*p-C*), 129.83 (*p-C*), 129.27 (*p-C*), 125.25 (m-C), 125.07(*m*-C), 121.38 (q, ^1^*J*_*CF*_ = 321.3 Hz), 115.70 (N*C*H), 29.07 (*C*H), 24.24 (*C*H_3_), 23.97(*C*H_3_), 23.20 (*C*H_3_).

^19^F-NMR (376 MHz, methylene chloride-*d*_2_): δ −78.93.

IR (ATR, 298 K): ν [cm^–1^] = 3354 (w), 2964 (m), 2929 (w), 2870 (w), 1611 (s), 1588 (w), 1568 (w), 1496 (m), 1461 (w), 1386 (w), 1366 (w), 1339 (w), 1273 (s),1222 (m), 1180 (w), 1145 (s), 1119 (w),1088 (w), 1061 (w), 1046 (w), 1032 (s), 935 (w),822 (w),803 (w),775 (w),758 (m), 688 (w),637 (s),593 (w), 571 (w), 517 (w), 447 (w), 437 (w), 426 (w), 408 (w).

ESI-MS: *m*/*z* = 870.71 [Cu(DippImH)_2_]^+^; 404.58 (DippImH + H)^+^; 150.70 (OTf)^−^.

Anal. Calcd. C_55_H_74_CuF_3_N_6_O_3_S: C, 64.78; H, 7.31; N, 8.24; S, 3.14. Found: C, 64.05; H, 7.60; N, 8.09; S, 2.71.

#### Synthesis of [Cu(DippImH)_2_][BAr^F^] (**3**)

[Cu(DippImH)_2_][OTf] (**2**) (691 mg, 0.67 mmol, 1.0 equiv) and NaBAr^F^ (601 mg, 0.67 mmol, 1.0 equiv) were placed in a beaker under ambient conditions and stirred in DCM (60 mL) for 1 h. The resulting solution was extracted with H_2_O (3 × 60 mL) and dried *in vacuo* giving 1.14 g of raw product. This was dissolved in 3 mL DCM and precipitated with 50 mL pentane. Double recrystallization followed by drying in high vacuum gives analytically pure **3** as white-off solid (1.08 g, 0.62, 92%).

^1^H NMR (500 MHz, chloroform-*d*): δ 7.71 (s, 8H, BAr^F^), 7.61 (s, 2H, *p*-Ar*H*), 7.52 (s, 4H, BAr^F^), 7.41 (s, 4H, *m*-Ar*H*), 6.91 (s, 4H, *m*-Ar*H*), 6.65 (s, 2H, *p*-Ar*H*), 6.53–6.39 (m, 4H, NC*H*), 2.63–2.35 (m, 8H, C*H*), 2.21 (s, 2H, N*H*), 1.32–0.93 (m, 48H, C*H*_3_).

^13^C NMR (126 MHz, chloroform-*d*): δ 162.23 (q, *J* = 49.8 Hz, BAr^F^), 153.87 (N*C*N), 147.66 (*o-C*), 134.94 (BAr^F^), 131.58 (*p-C*), 130.67 (*p-C*), 129.68 (*p-C*), 129.36 (q, J = 31.5 Hz, BAr^F^), 125.06 (*m-C*), 124.88 (*m-C*), 124.70 (q, *J* = 272.4 Hz, BAr^F^), 117.56 (p, *J* = 3.9 Hz, BAr^F^), 115.35 (N*C*H), 28.88 (*C*H), 24.19 (*C*H_3_), 23.87 (*C*H_3_), 23.07 (*C*H_3_). * Signal of *ipso-C* is not observed.

^19^F-NMR (376 MHz, chloroform-*d*): δ −62.45.

IR (ATR, 298 K): ν [cm^–1^] = 3352 (w), 2968 (w), 2931 (w), 2874 (w), 1621 (m), 1611 (m), 1588 (w), 1566 (w), 1498 (w), 1475 (w), 1467 (w), 1388 (w), 1366 (w), 1351 (m),1273 (s), 1226 (w), 1211 (w), 1162 (m), 1125 (s), 1061 (w), 1046 (w), 937 (w), 886 (w), 838 (w), 822 (w), 803 (w), 775 (w), 756 (w), 746 (w), 713 (w), 682 (m), 667 (m), 608 (w), 470 (w), 447 (w), 416 (w).

ESI-MS: *m*/*z* = 870.71 [Cu(DippImH)_2_]^+^; 404.58 (DippImH + H)^+^; 863.20 (BAr^F^)^−^.

Anal. Calcd C_86_H_86_BCuF_24_N_6_: C, 59.57; H, 5.00; N, 4.85. Found: C, 59.35; H, 5.33; N, 4.85.

#### Synthesis of [Cu_3_(DippIm)_2_Cl] (**4**)

A 100 mL Schlenk flask, equipped with reflux condenser was charged with CuCl (250 mg, 2.53 mmol, 1.0 equiv) and bis(2,6-diisopropylphenyl)imidazolin-2-imino lithium, DippImLi, (1.03 g, 2.53 mmol, 1.0 equiv). Then diethyl ether (40 mL) was added, and the reaction mixture was heated under reflux overnight. The resulting yellow solution was dried *in vacuo* giving a foamy material, which was extracted with 20 mL benzene (or toluene) and filtered. After the solvent was removed *in vacuo*, the raw product was washed with hexane (3 × 2 mL) and dried, giving 0.82 g of the material containing **4**. Crystals of **4**, suitable for SX-XRD were grown in hexane by cooling at −40 °C.

#### Synthesis of [Cu(AlCp*)_4_][BAr^F^] (**5**)

A Schlenk tube was charged with [Cu(DippImH)_2_][BAr^F^] (**3**) (200 mg, 0.115 mmol, 1.0 equiv) and AlCp* (75 mg, 0.46 mmol, 4.0 equiv). Then THF (7 mL) was added, and the tube was heated for 3 h at 70 °C. The resulting slightly yellowish solution was concentrated to 3 mL. Addition of hexane (10 mL) gives a white precipitate, which was filtered off, washed with hexane (3 × 3 mL), and dried *in vacuo* giving the product as a white solid (123 mg, 0.08 mmol, 68%). Caution! Compound **5** is air-sensitive and pyrophoric. Its residues were quenched with isopropanol.

^1^H NMR (400 MHz, THF-*d*_8_): δ 7.80 (s, 8H, BAr^F^), 7.58 (s, 4H, BAr^F^), 1.95 (s, 60H, Cp*).

^13^C NMR (101 MHz, THF-*d*_8_): δ 163.00 (q, *J* = 49.7 Hz, BAr^F^), 135.79, 130.16 (q, *J* = 32.3 Hz, BAr^F^), 125.70 (q, *J* = 272.2 Hz, BAr^F^), 118.64–118.14 (m, BAr^F^), 115.41 (*C*_5_Me_5_), 10.18(C_5_*Me*_5_).

^19^F-NMR (376 MHz, THF-*d*_8_): δ −63.39.

^27^Al-NMR (104 MHz, THF): δ −57.74.

IR (ATR, 298 K): ν [cm^–1^] = 2960 (w), 2923 (w), 2870 (w), 1609 (w), 1483 (w), 1452 (w), 1419 (w), 1382 (w), 1351 (m), 1271 (s), 1170 (m), 1149 (m), 1125 (s), 1026 (w), 946 (w), 925 (w), 898 (m), 886 (m), 838 (m), 799 (w), 744 (w), 715 (m), 682 (m), 667 (m), 585 (w), 486 (s), 447 (w), 426 (w), 412 (w).

Anal. Calcd C_72_H_72_Al_4_BCuF_24_: C, 54.89; H, 4.61; Al, 6.85; Cu, 4.03. Found: C, 54.64; H, 4.43; Al, 6.5; Cu, 4.0.

## Supporting Information Available

NMR spectra for all compounds, FT-IR spectra for all compounds, mass spectrometric data, and crystallographic details for all compounds (PDF)
