# Supplementary material for: Copper Imidazolin-imine Coordination Compounds as Precursors for a Cu/Al Complex
Source: Inorg Chem. 2024 Sep 11;63(38):17331–9. doi: 10.1021/acs.inorgchem.4c02530 (PMC11423405; doi:10.1021/acs.inorgchem.4c02530)
Supplement: Supplementary file 1 — ic4c02530_si_001.pdf [file ic4c02530_si_001.pdf]

Supporting Information

Copper Imidazolin-imine Coordination Compounds as Precursors for a Cu/Al Complex.

*Ivan Antsiburov, Johannes Stephan, Richard J.J. Weininger, Christian Gemel and Roland A. Fischer**

Technical University of Munich, School of Natural Sciences, Department of Chemistry, Chair of Inorganic and Metal-Organic Chemistry Lichtenbergstr. 4, 85748 Garching, Germany.

Technical University of Munich, Catalysis Research Centre, Ernst-Otto-Fischer Str. 1, 85748 Garching, Germany.

E-mail: roland.fischer@tum.de

1. NMR Data ………………………………………………………………3
2. FT-IR Data……………………………………………………………..15
3. MS-Data………………………………………………………………..18
4. Crystallography 19
5. Literature 26

1. NMR-Spectra

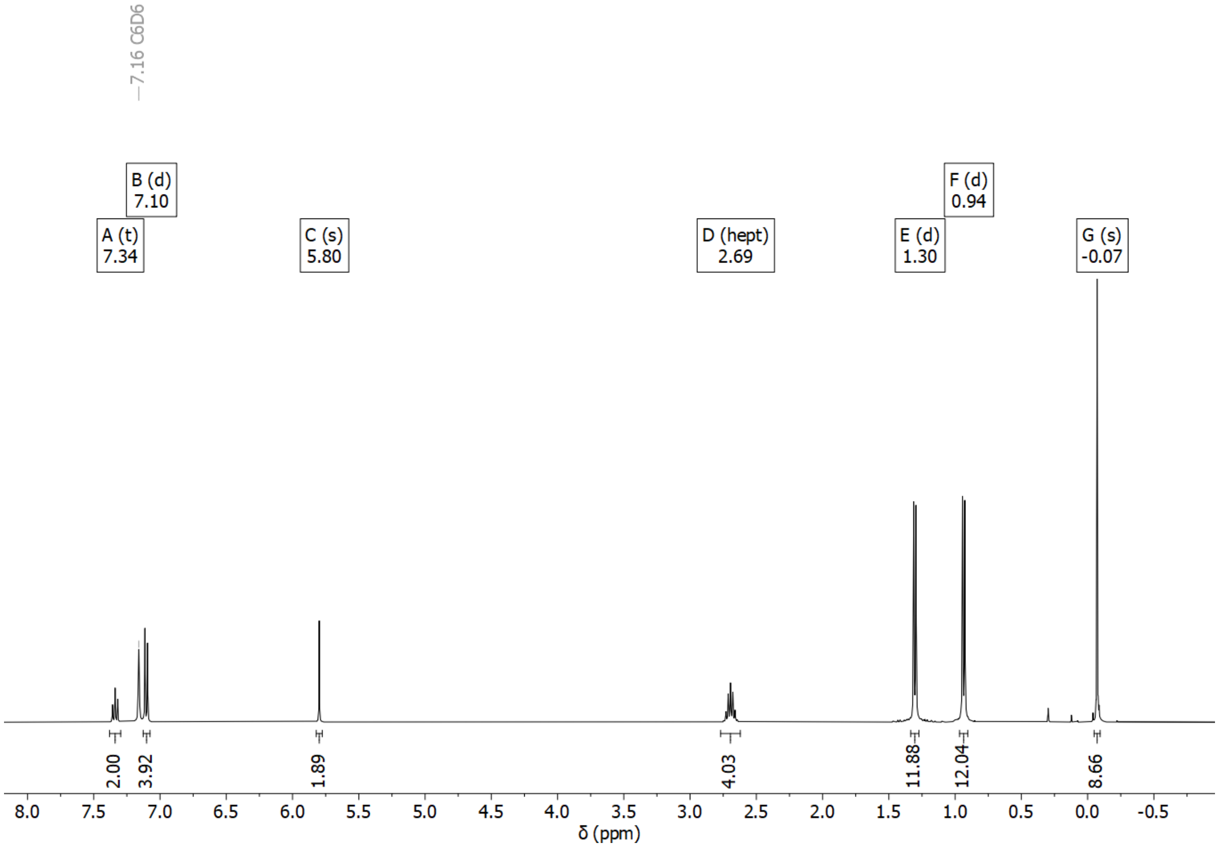

Figure S1: ^{1}H-NMR (benzene-d_6) spectrum of [Cu(DippImTMS)(OTf)] (**1**).

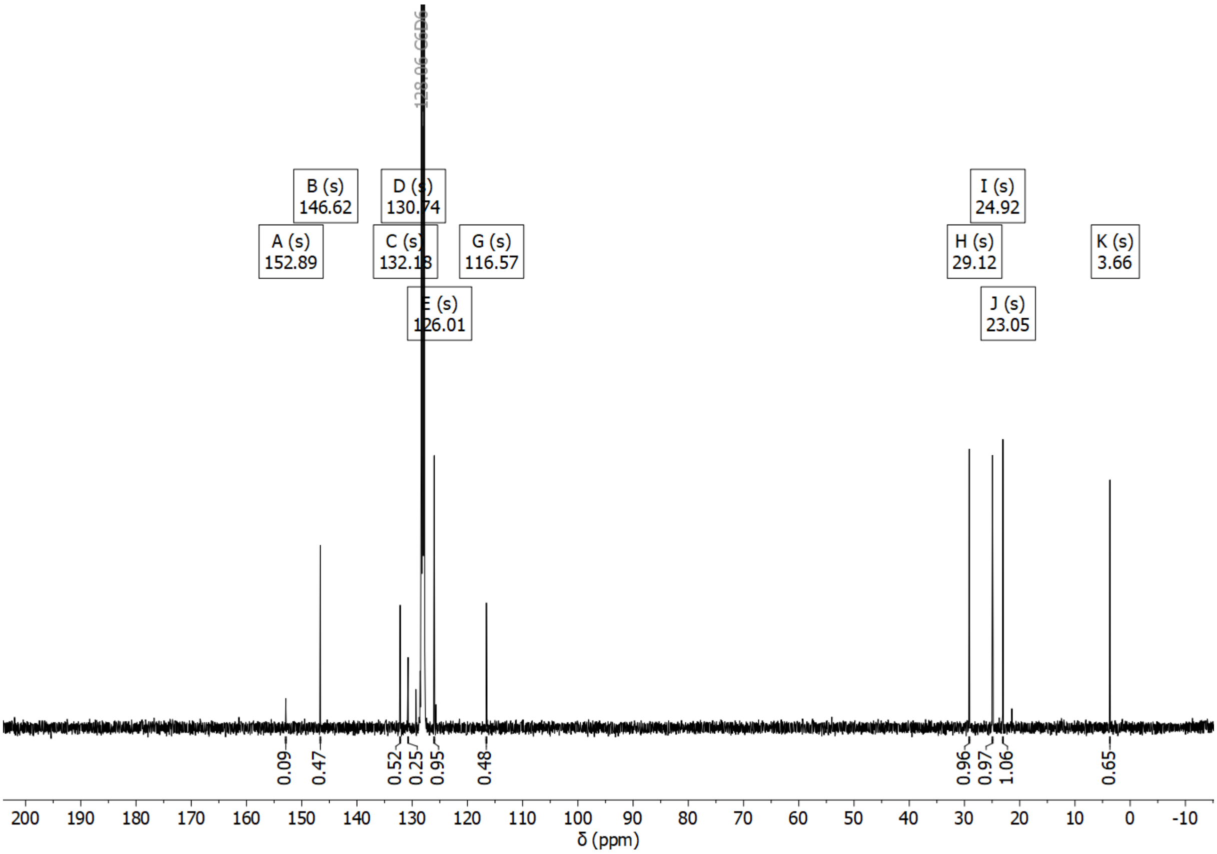

Figure S2: ^{13}C-NMR (benzene-d$_6$) spectrum of [Cu(DippImTMS)(OTf)] (**1**).

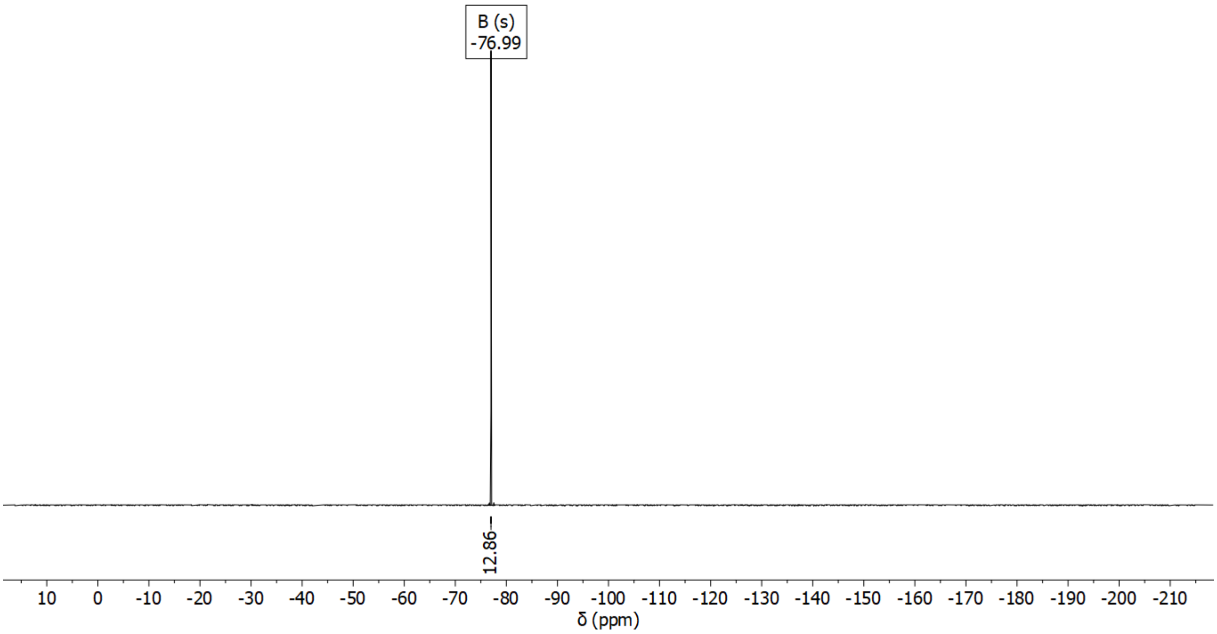

Figure S3: ^{19}F-NMR (benzene-d$_6$) spectrum of [Cu(DippImTMS)(OTf)] (**1**).

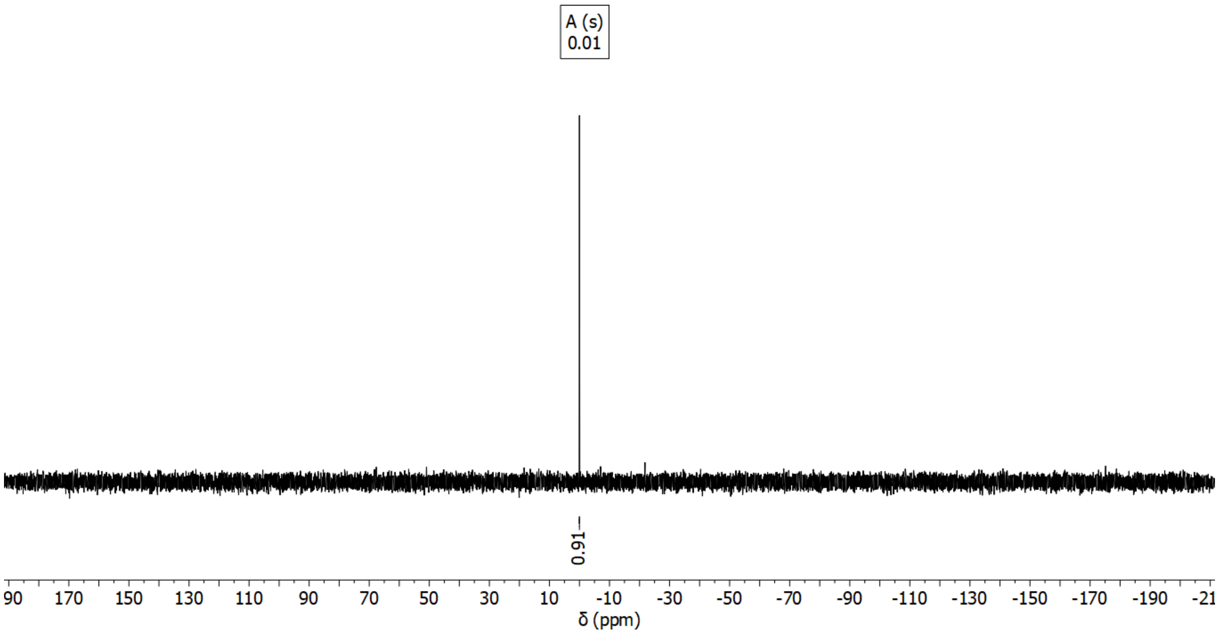

Figure S4: ^{29}Si-NMR (benzene-d_6) spectrum of [Cu(DippImTMS)(OTf)] (**1**).

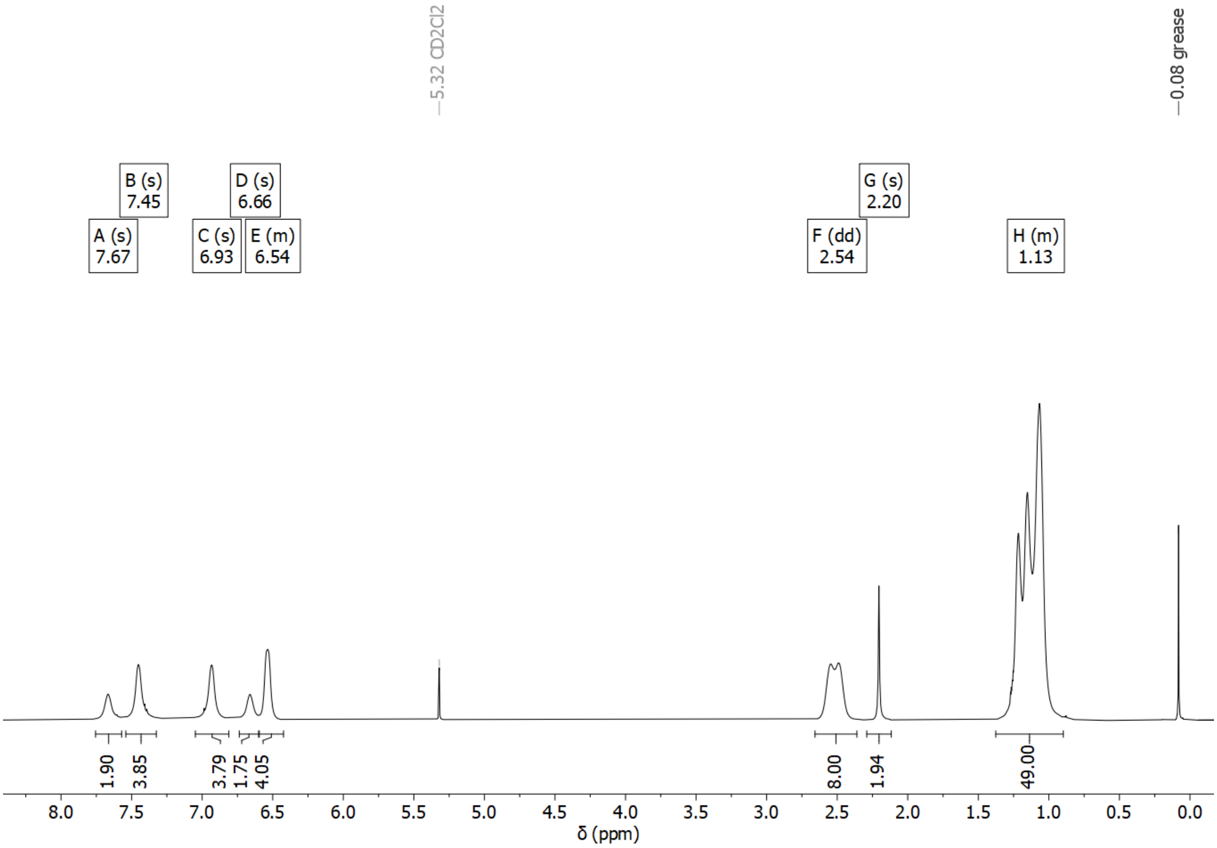

Figure S5: ^{1}H-NMR (CD_2Cl_2) spectrum of [Cu(DippImH)$_2$][OTf] (**2**).

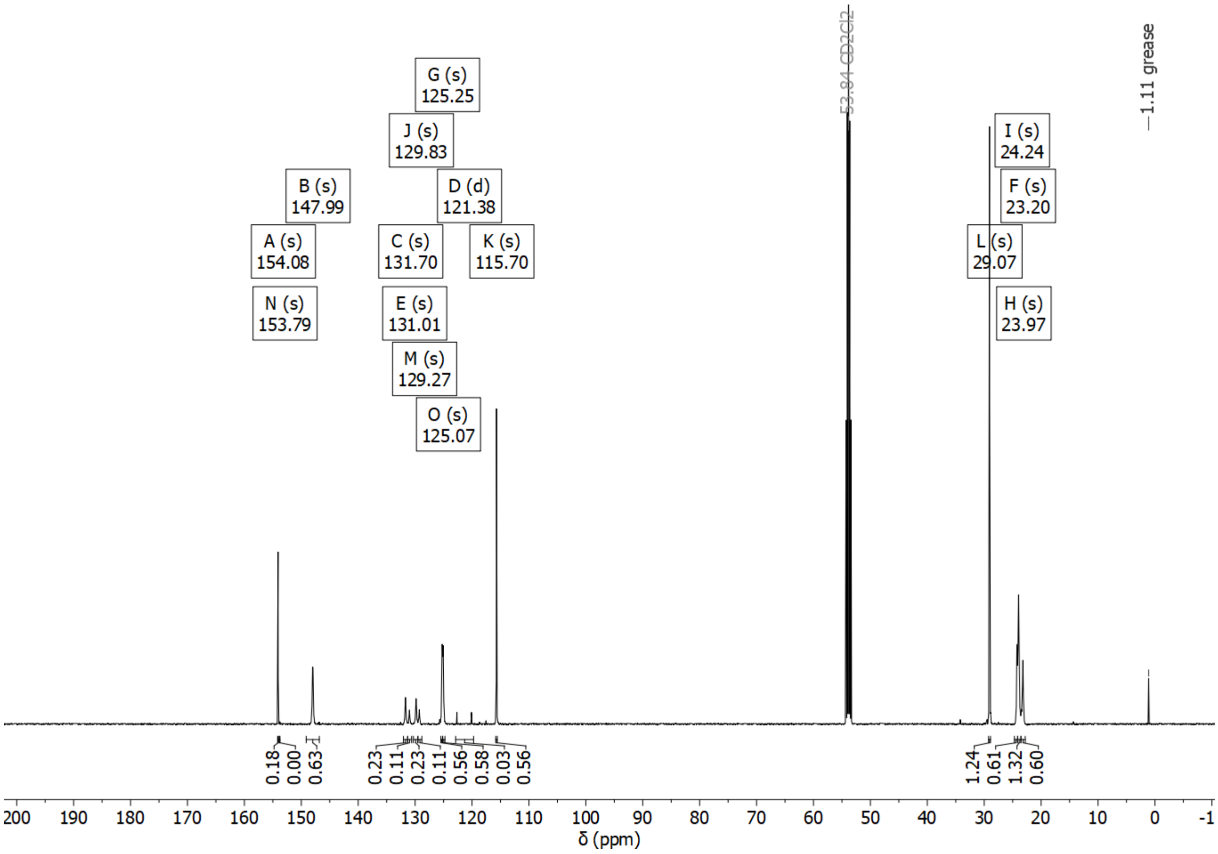

Figure S6: ^{13}C-NMR (CD_2Cl_2) spectrum of [Cu(DippImH)$_2$][OTf] (**2**).

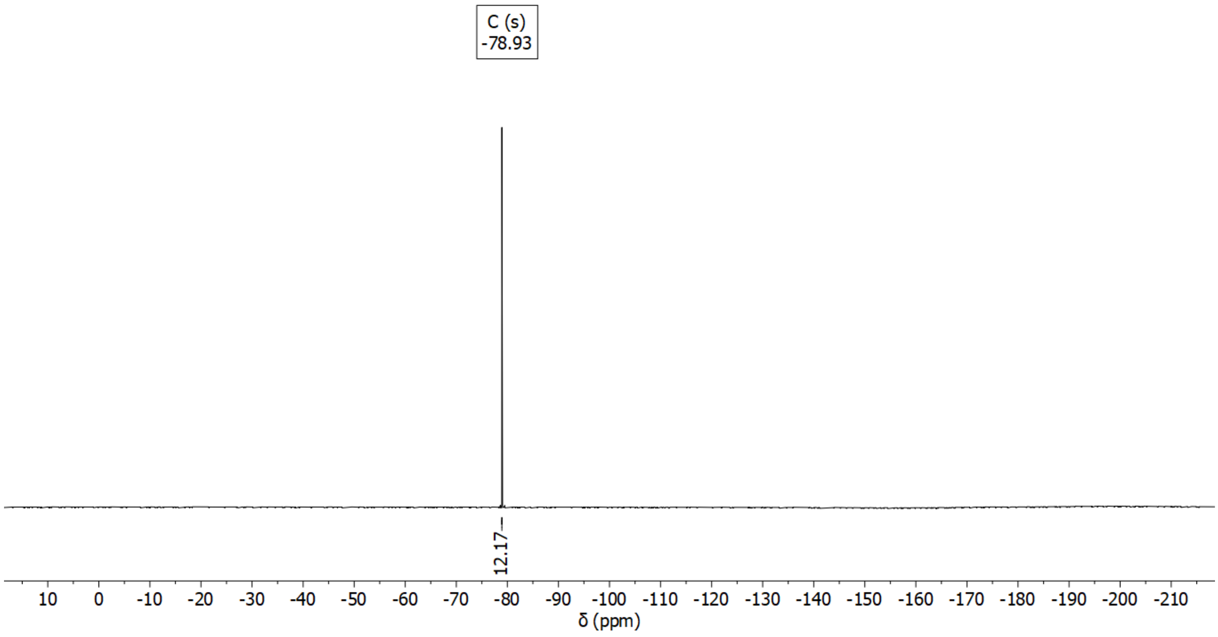

Figure S7: ^{19}F-NMR (CD_2Cl_2) spectrum of [Cu(DippImH)$_2$][OTf] (**2**).

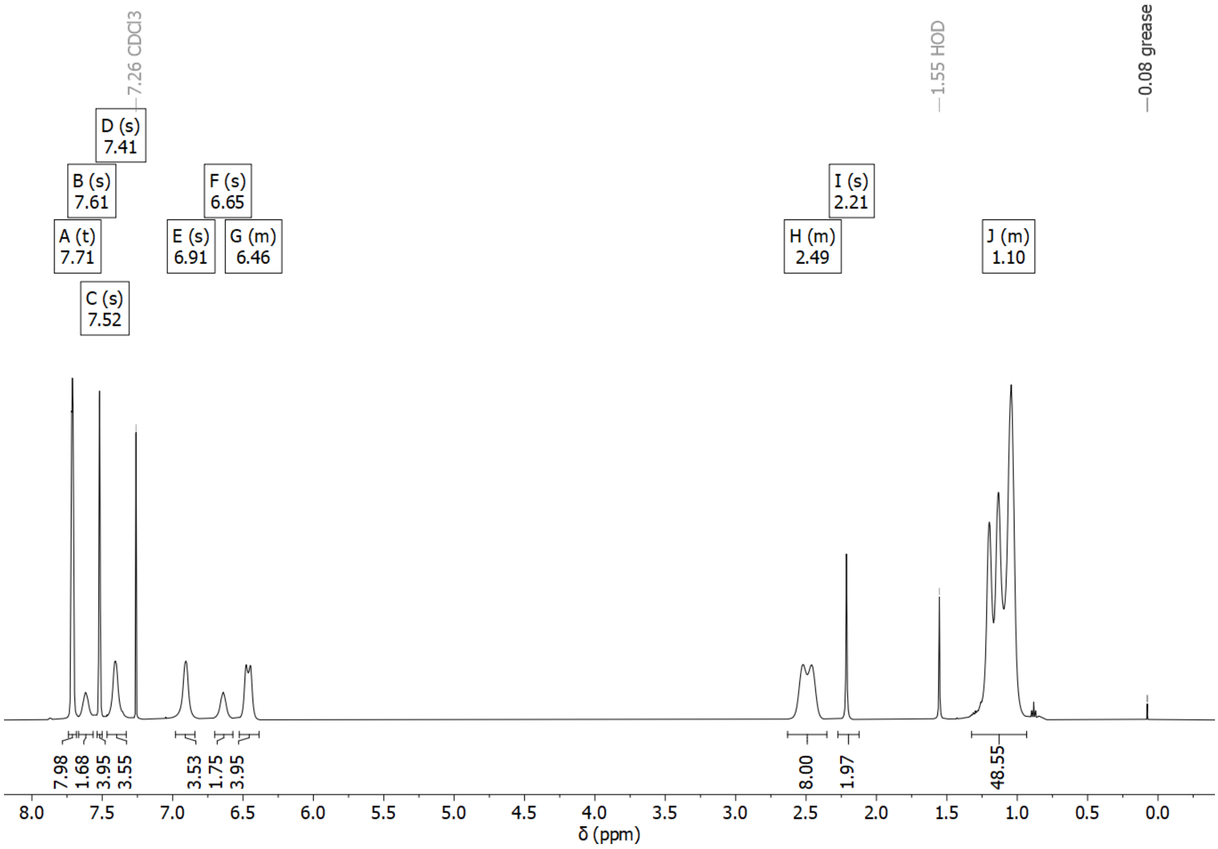

Figure S8: ^{1}H-NMR (chloroform-d) spectrum of [Cu(DippImH)$_2$][BAr^F] (**3**).

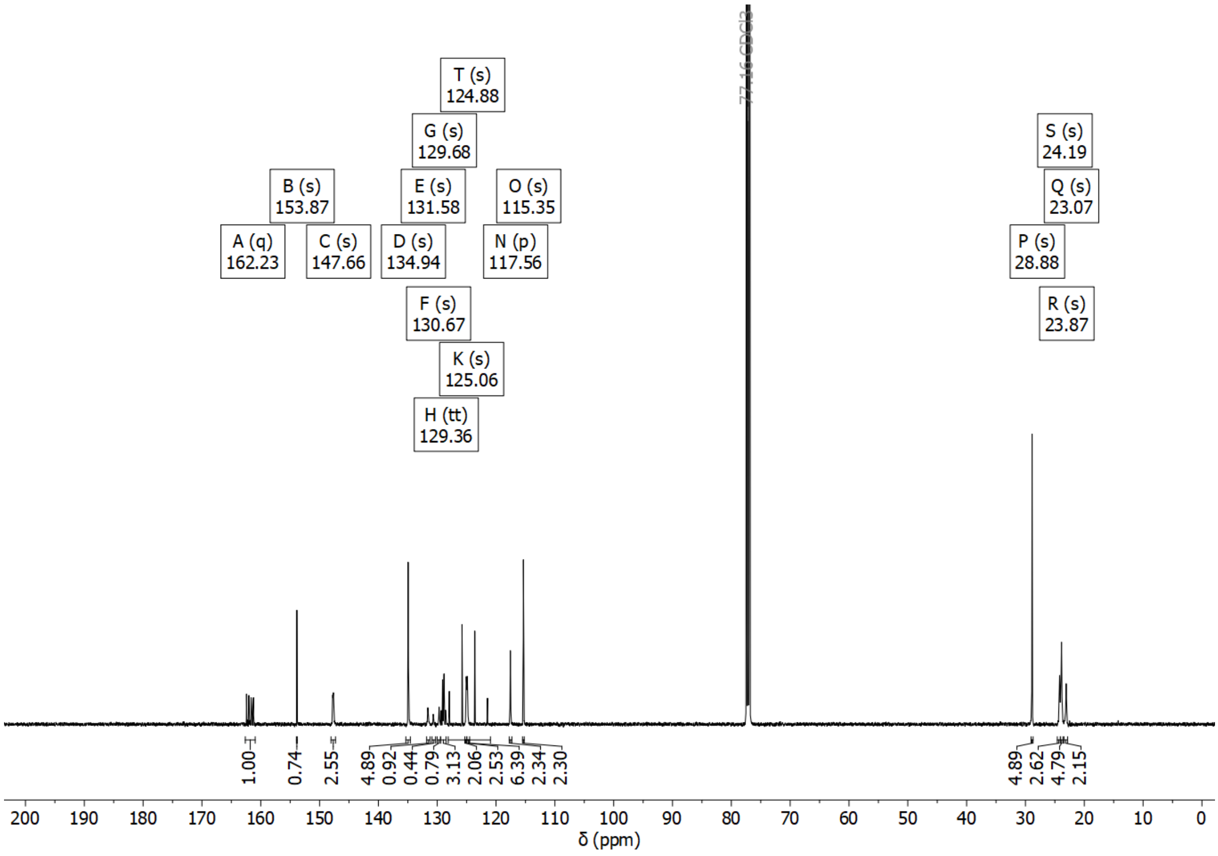

Figure S9: ^{13}C-NMR (chloroform-d) spectrum of [Cu(DippImH)$_2$][BAr^F] (**3**).

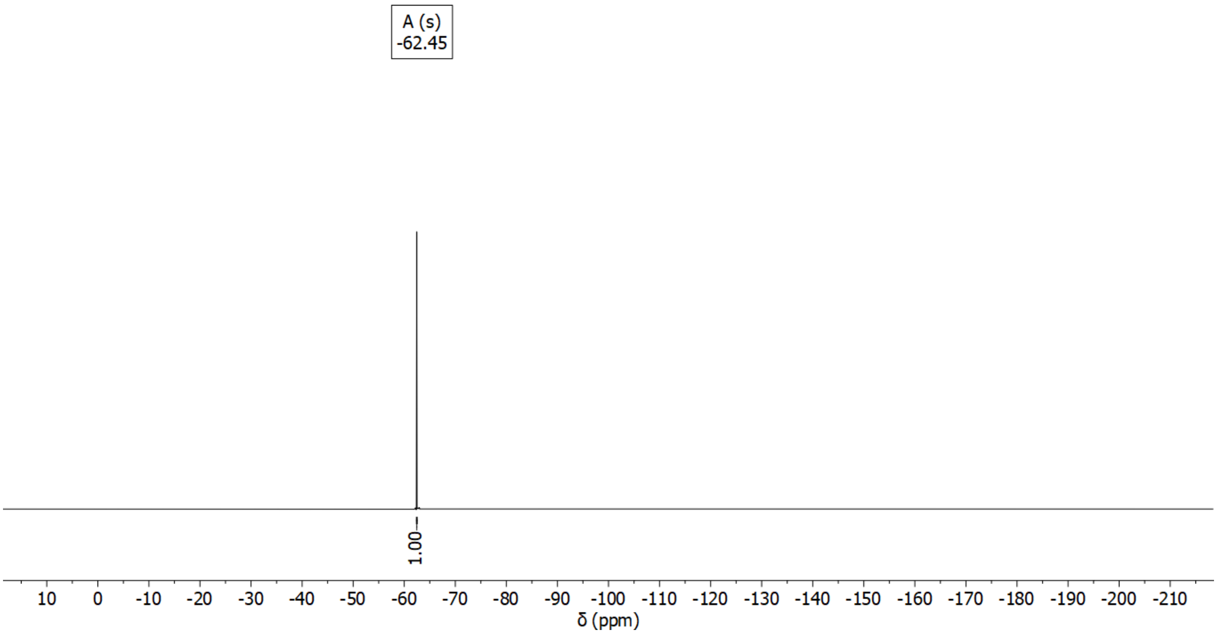

Figure S10: ^{19}F-NMR (chloroform-d) spectrum of [Cu(DippImH)$_2$][BAr^F] (**3**).

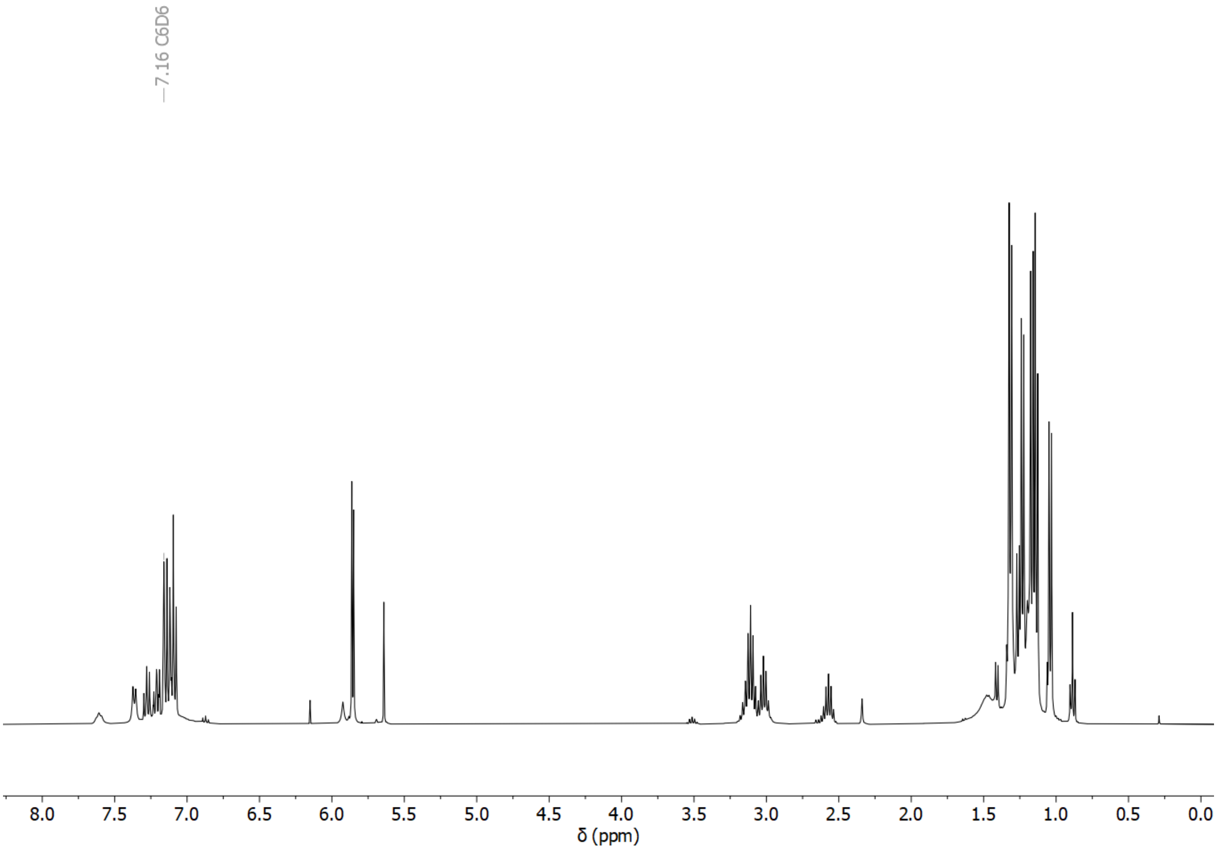

Figure S11: ^{1}H-NMR (benzene-d$_6$) spectrum of the sample, from which [Cu$_3$(DippIm)$_2$Cl] (**4**) was crystallized.

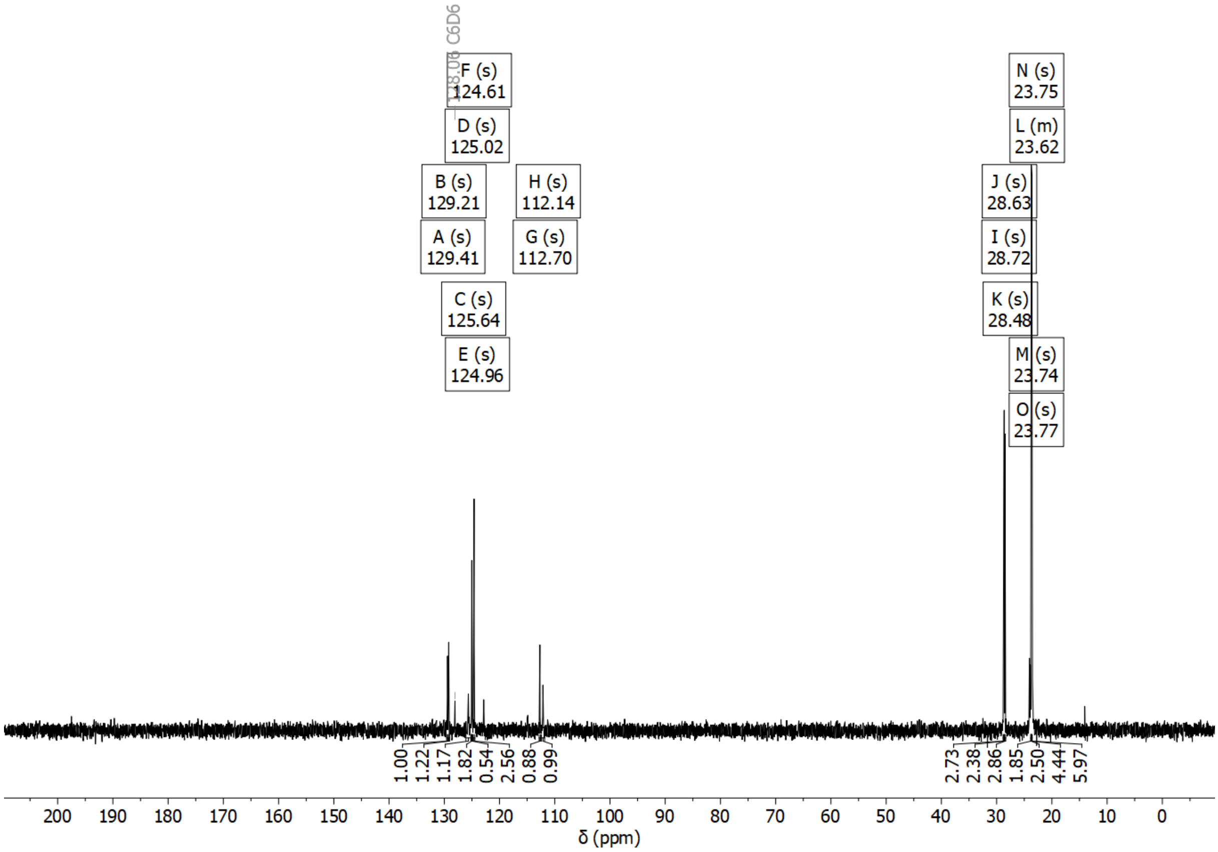

Figure S12: DEPT 135-NMR (benzene-d_6) spectrum of the sample, from which [Cu_3(DippIm)$_2$Cl] (**4**) was crystallized.

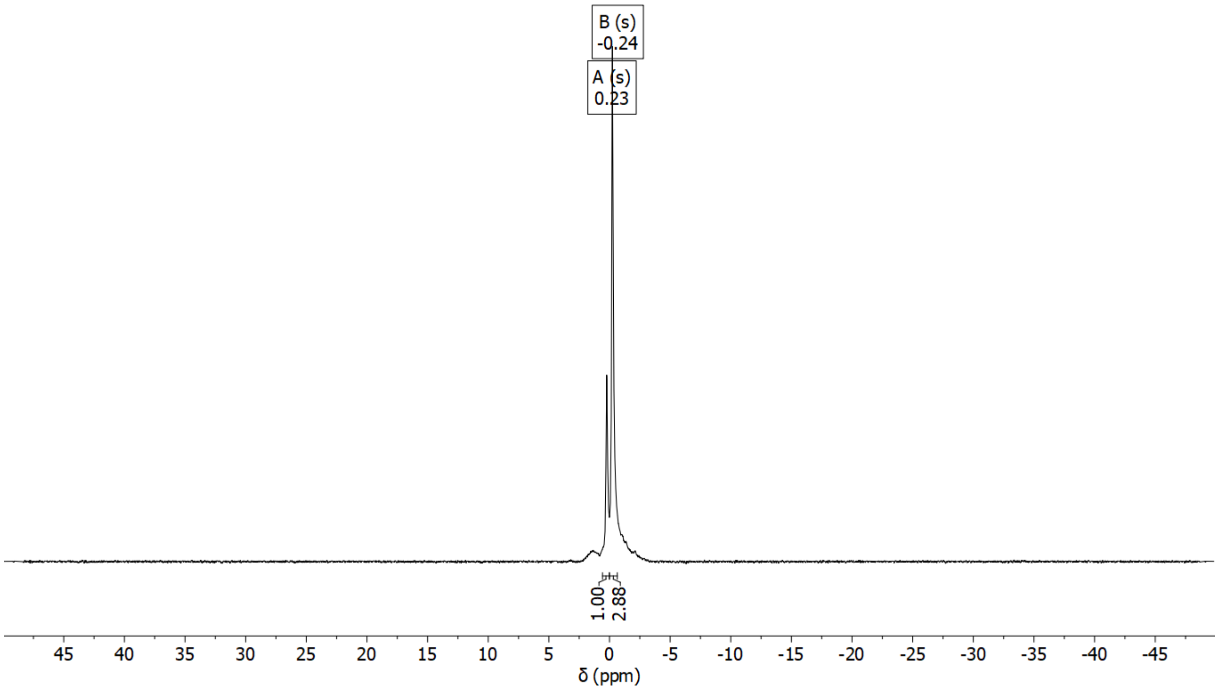

Figure S13: ^{7}Li-NMR (benzene-d_6) spectrum of the sample, from which [Cu_3(DippIm)$_2$Cl] (**4**) was crystallized.

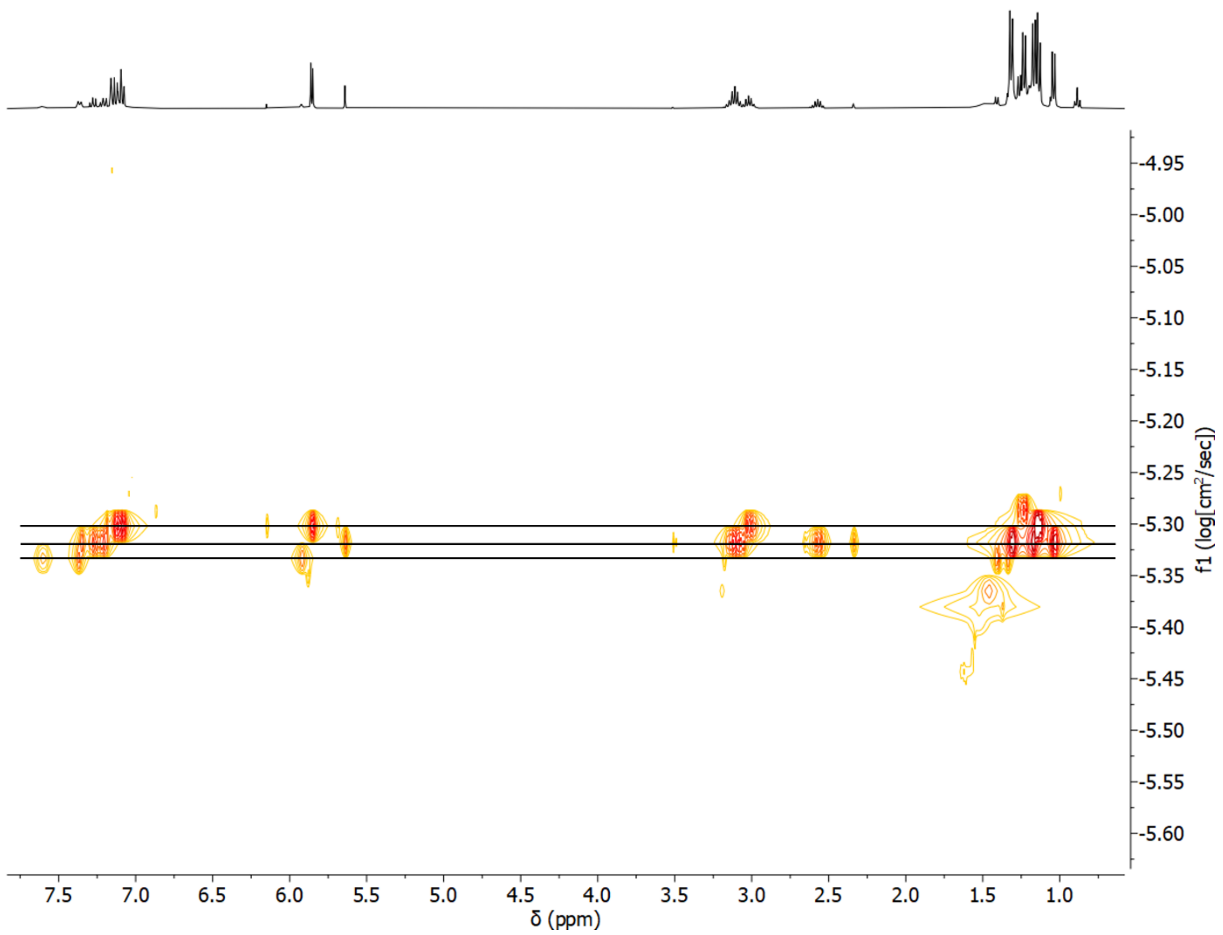

Figure S14: DOSY-NMR (benzene-d_6) spectrum of the sample, from which [$Cu_3(DippIm)_2Cl$] (**4**) was crystallized.

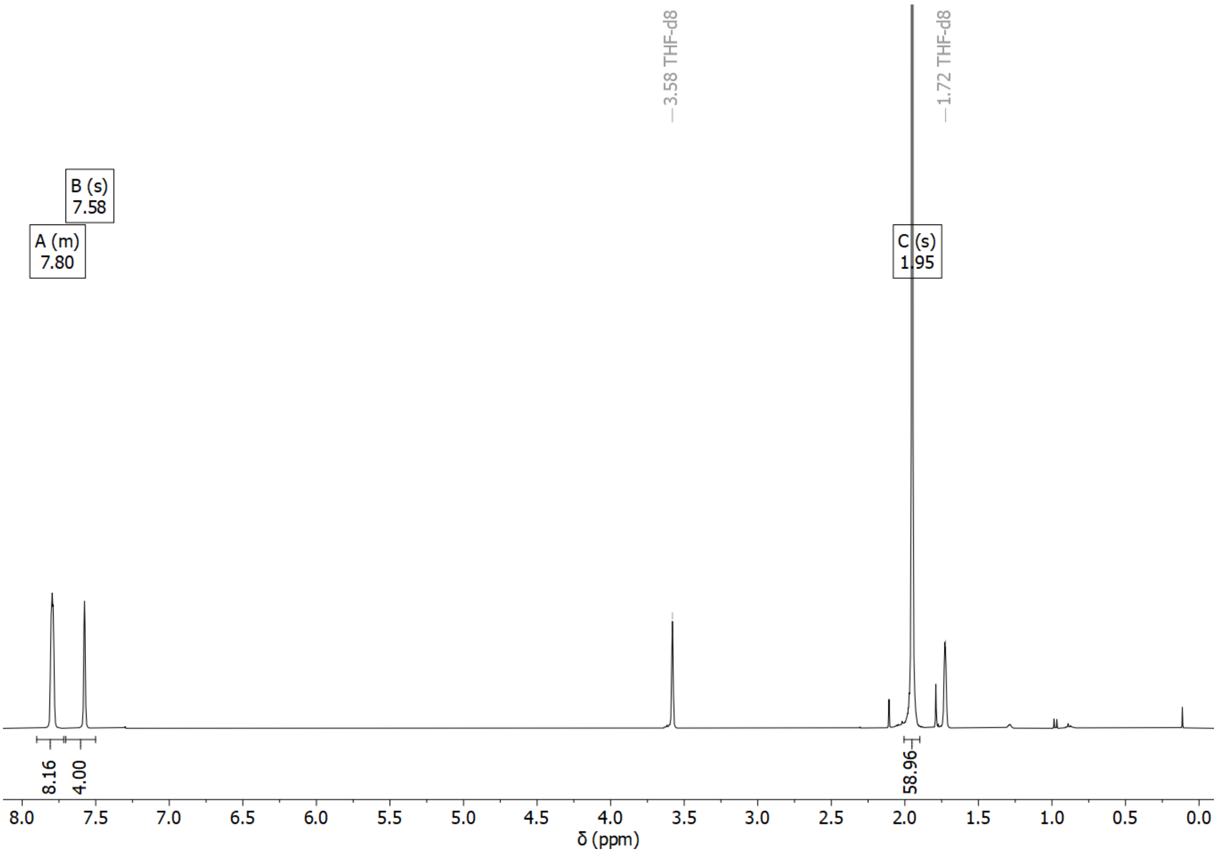

Figure S15: ^{1}H-NMR (THF-d8) spectrum of [Cu(AlCp*)$_4$][BAr^F] (**5**).

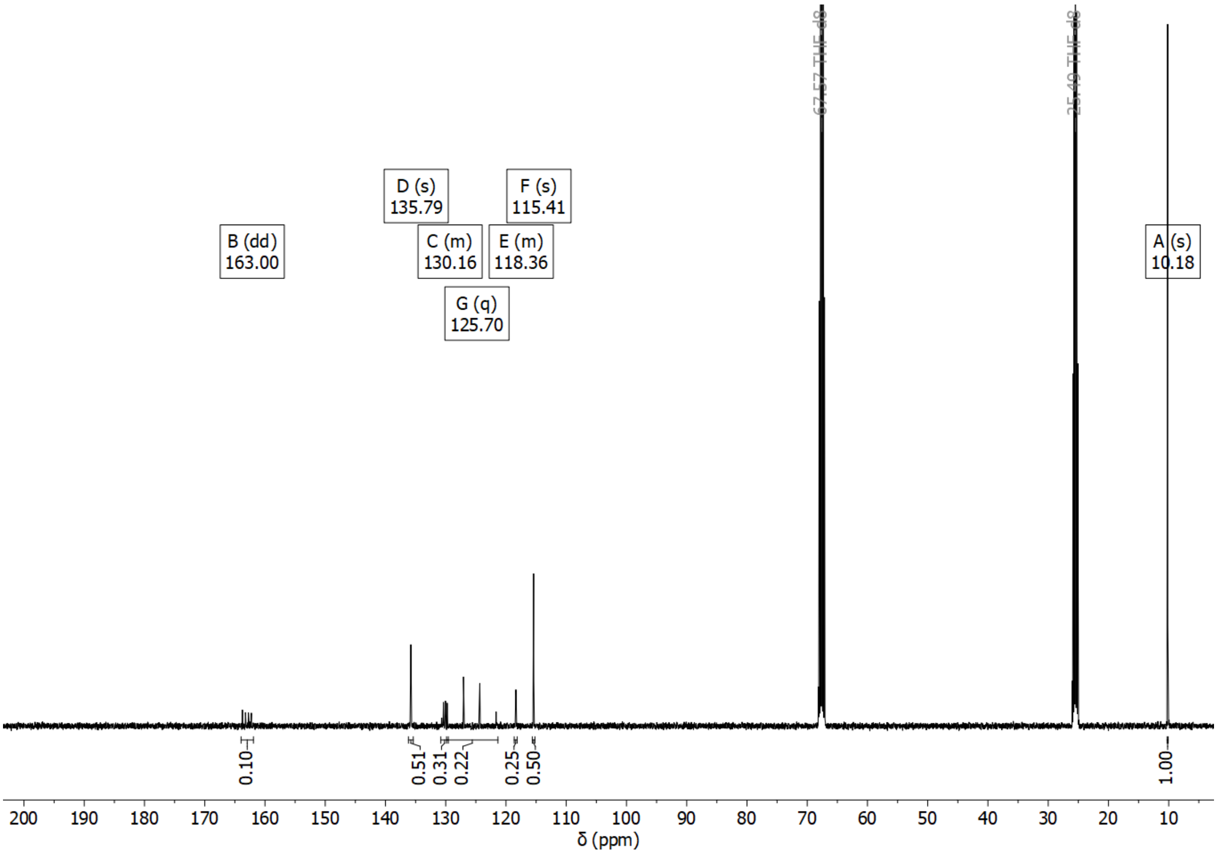

Figure S16: ^{13}C-NMR (THF-d8) spectrum of [Cu(AlCp*)$_4$][BAr^F] (**5**).

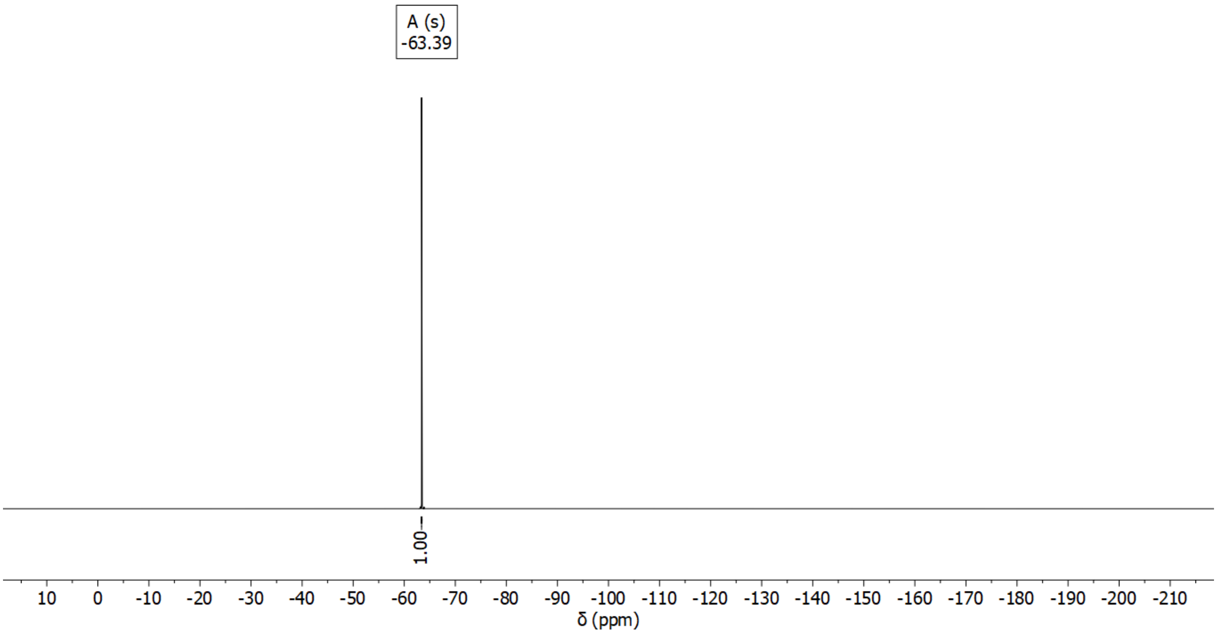

Figure S17: ^{19}F-NMR (THF-d8) spectrum of [Cu(AlCp*)$_4$][BAr^F] (**5**).

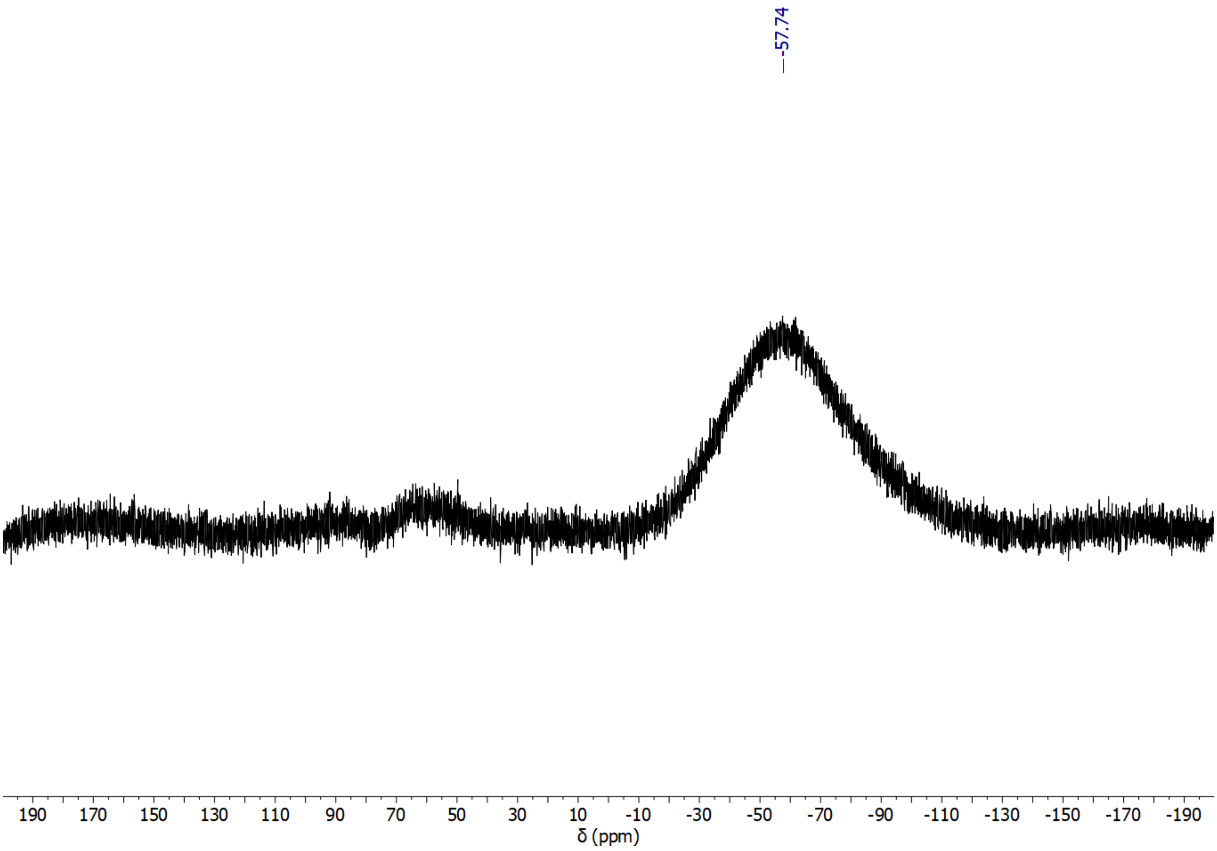

Figure S18: ^{27}Al-NMR (THF-d_8) spectrum of [Cu(AlCp*)$_4$][BAr^F] (**5**); background signal of aluminium in glass was removed by subtraction of blank THF-d8 spectrum.

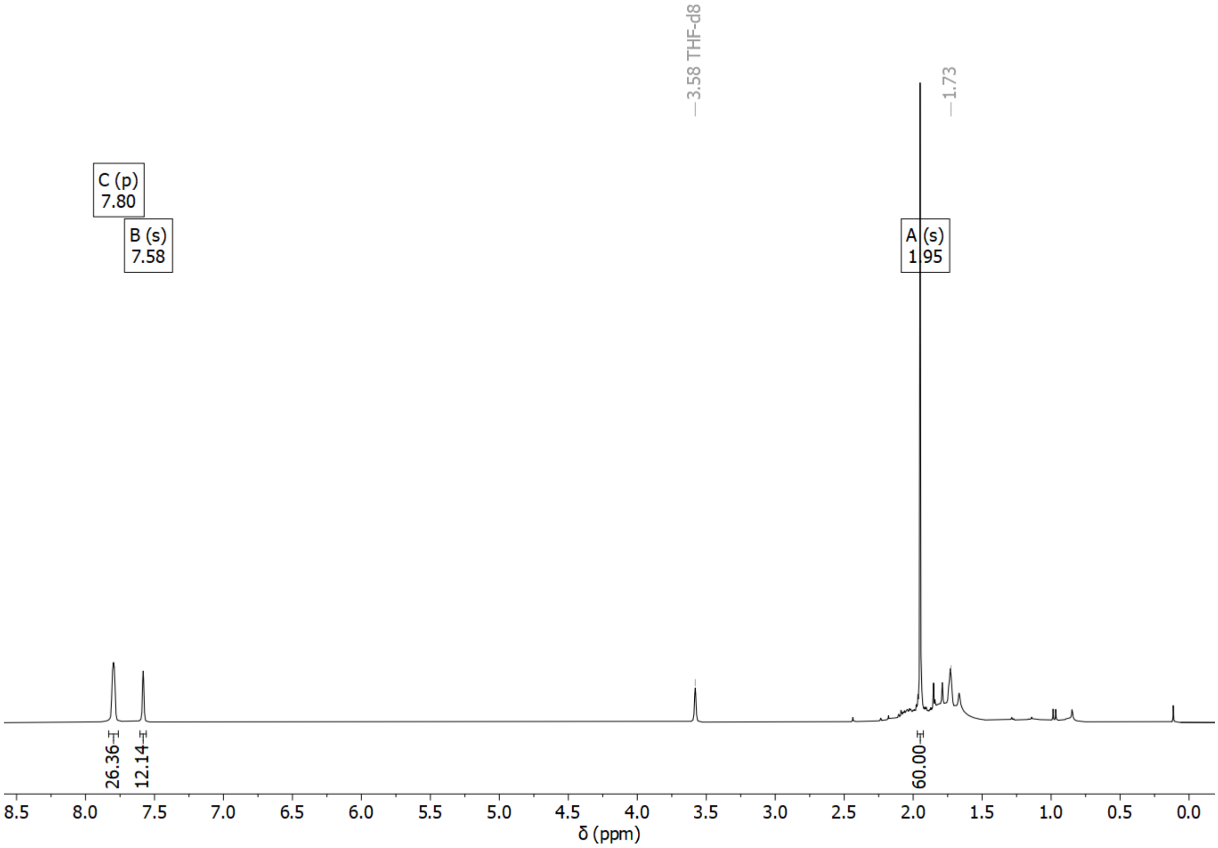

Figure S19: *In situ* ^{1}H-NMR spectra (THF-d8) of the reaction $[Cu(MeCN)_4][BAr^F]$ + AlCp* (1:4, 70 °C, 3 h).

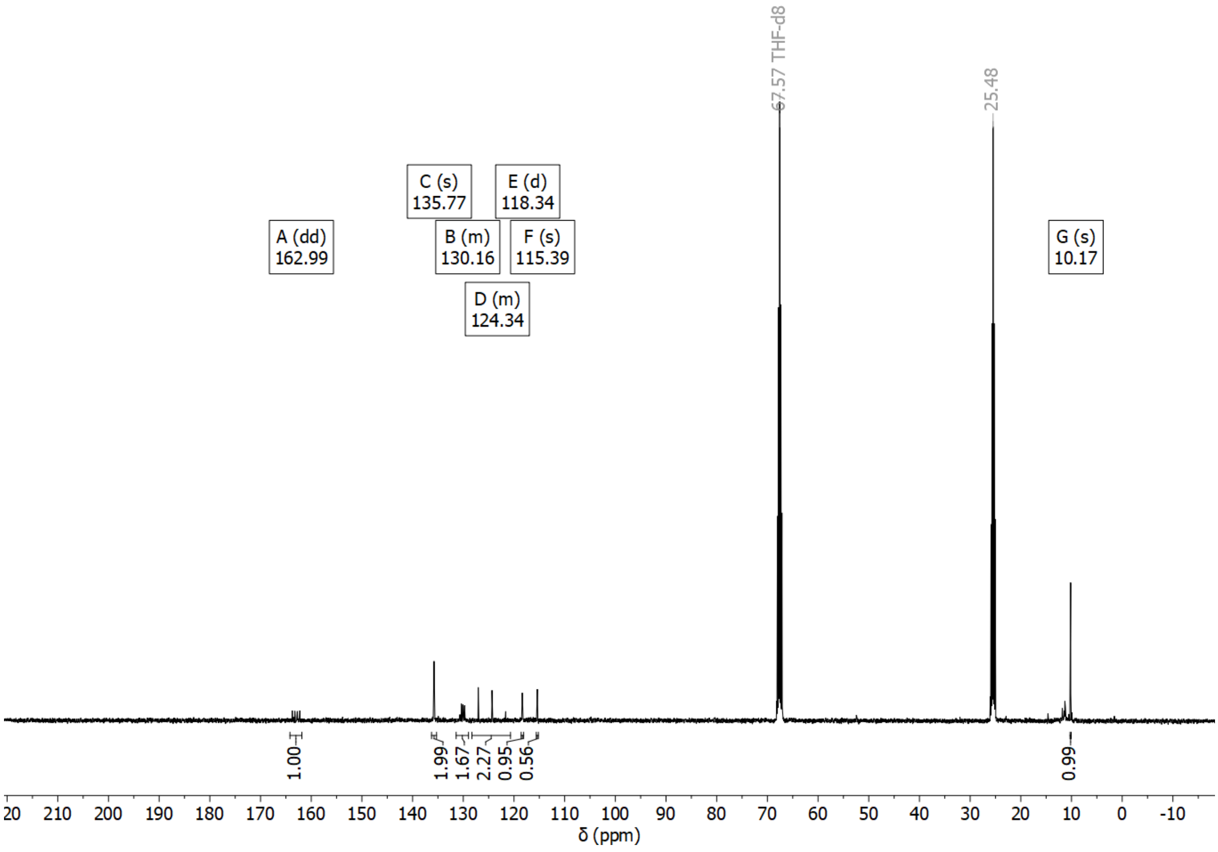

Figure S20: *In situ* ^{13}C-NMR spectra (THF-d8) of the reaction $[Cu(MeCN)_4][BAr^F]$ + AlCp* (1:4, 70 °C, 3 h).

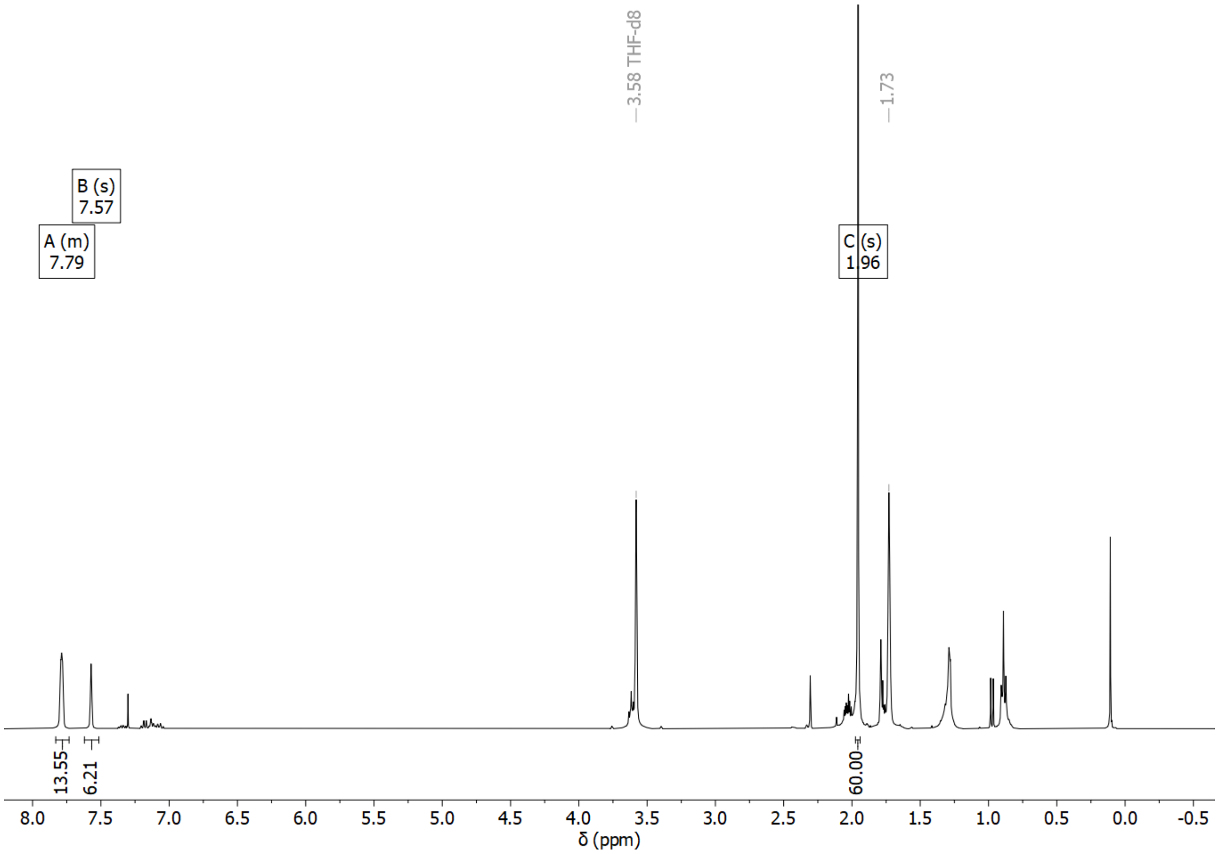

Figure S21: ^{1}H-NMR (THF-d8) spectrum of the reaction $[Cu(cod)_2][BAr^F]$ + AlCp* (1:4, r.t., 1 h).

2. IR-Spectra

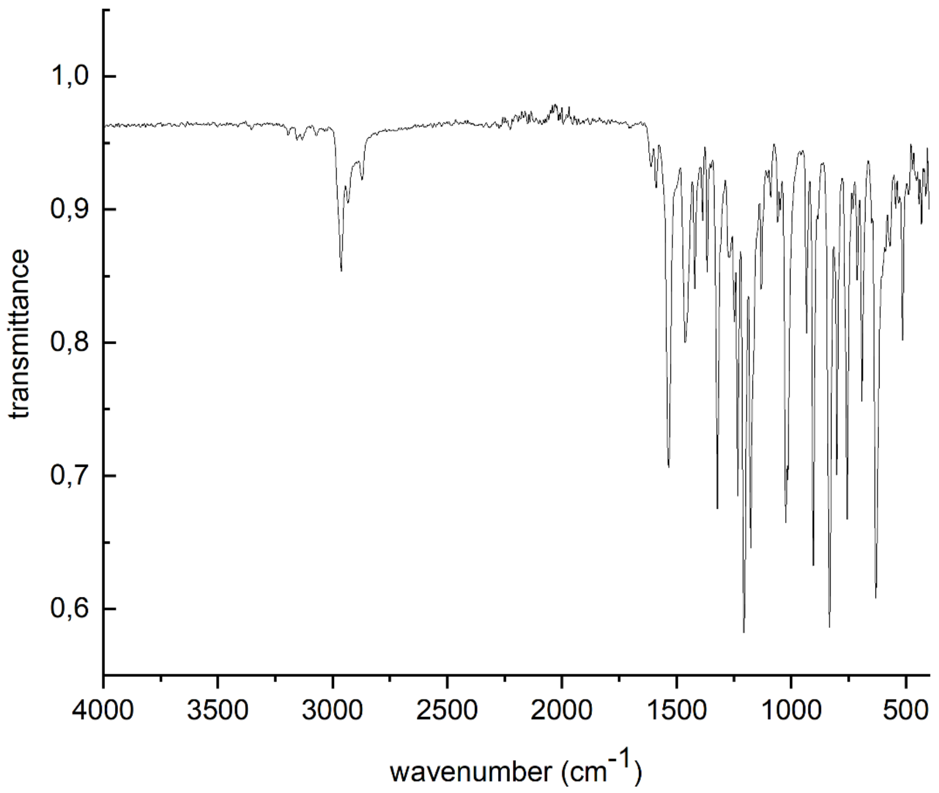

Figure S22: ATR-IR spectrum of [Cu(DippImTMS)(OTf)] (**1**).

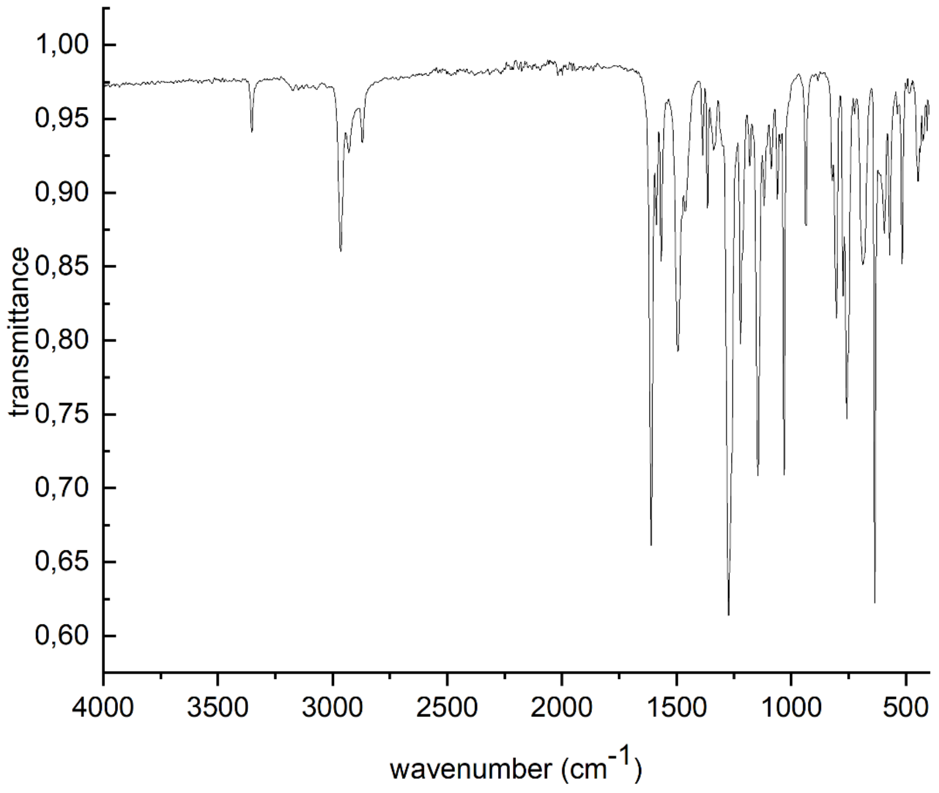

Figure S23: ATR-IR spectrum of [Cu(DippImH)$_2$][OTf] (**2**).

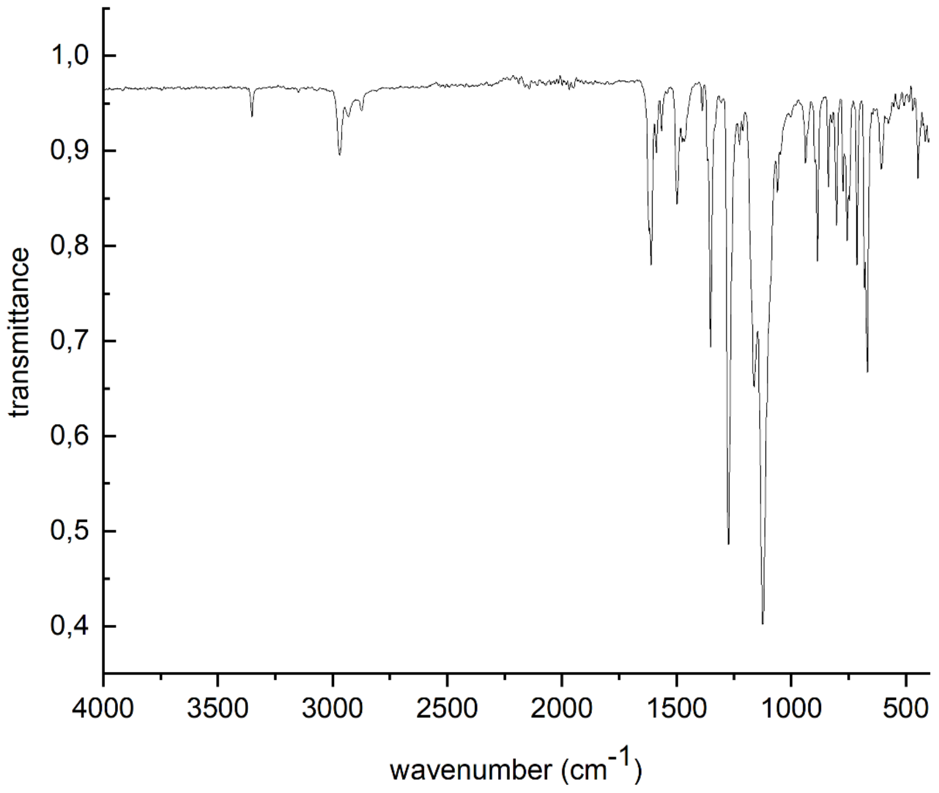

Figure S24: ATR-IR spectrum of $[Cu(DippImH)_2][BAr^F]$ (**3**).

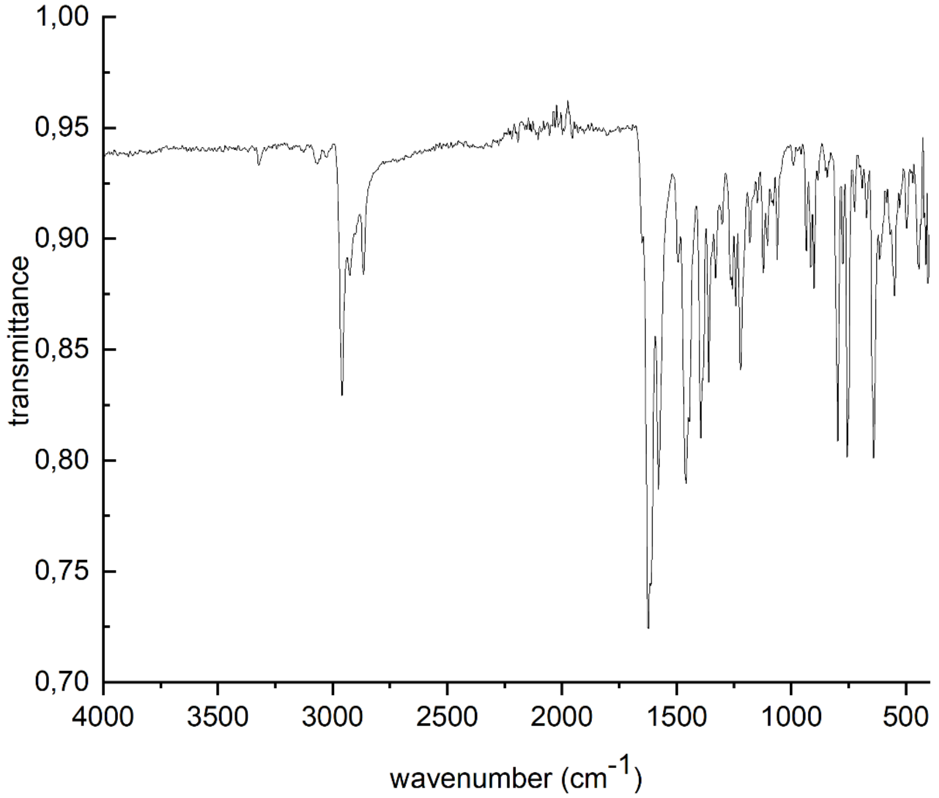

Figure S25: ATR-IR spectrum of the sample, from which $[Cu_3(DippIm)_2Cl]$ (**4**) was crystallized.

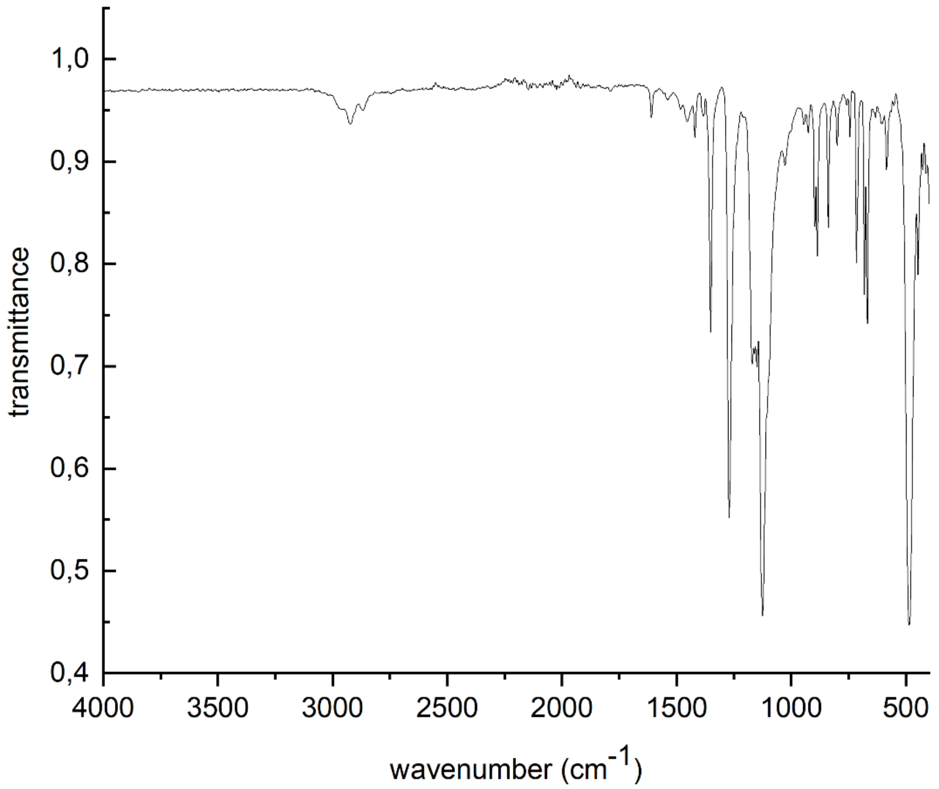

Figure S26: ATR-IR spectrum of [Cu(AlCp*)$_4$][BAr^F] (**5**).

3. Mass spectrometry

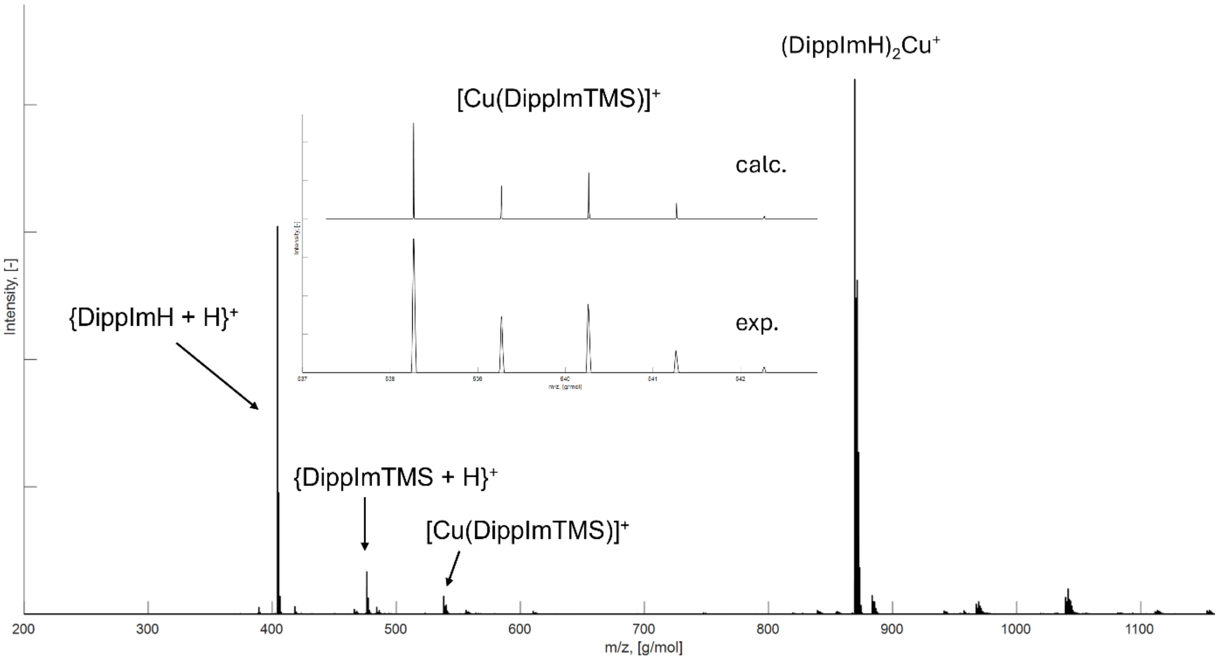

Figure S27: ESI-MS (positive) spectrum of [Cu(DippImTMS)(OTf)] (**1**).

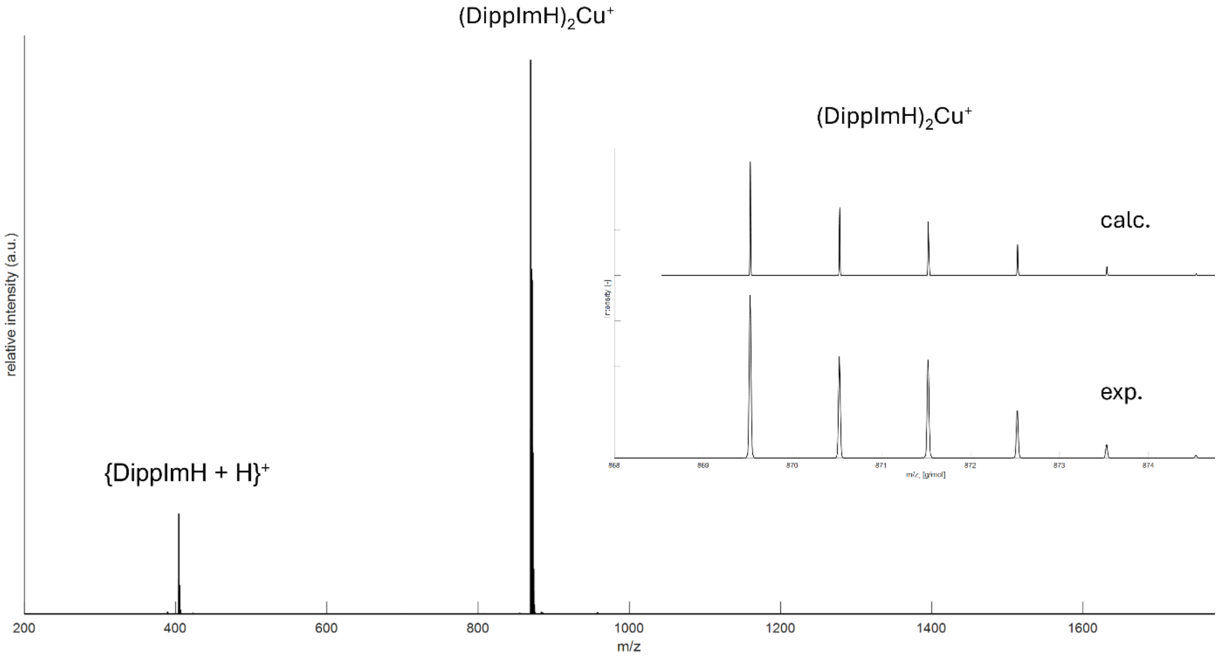

Figure S28: ESI-MS (positive) spectrum of [Cu(DippImH)$_2$][OTf] (**2**).

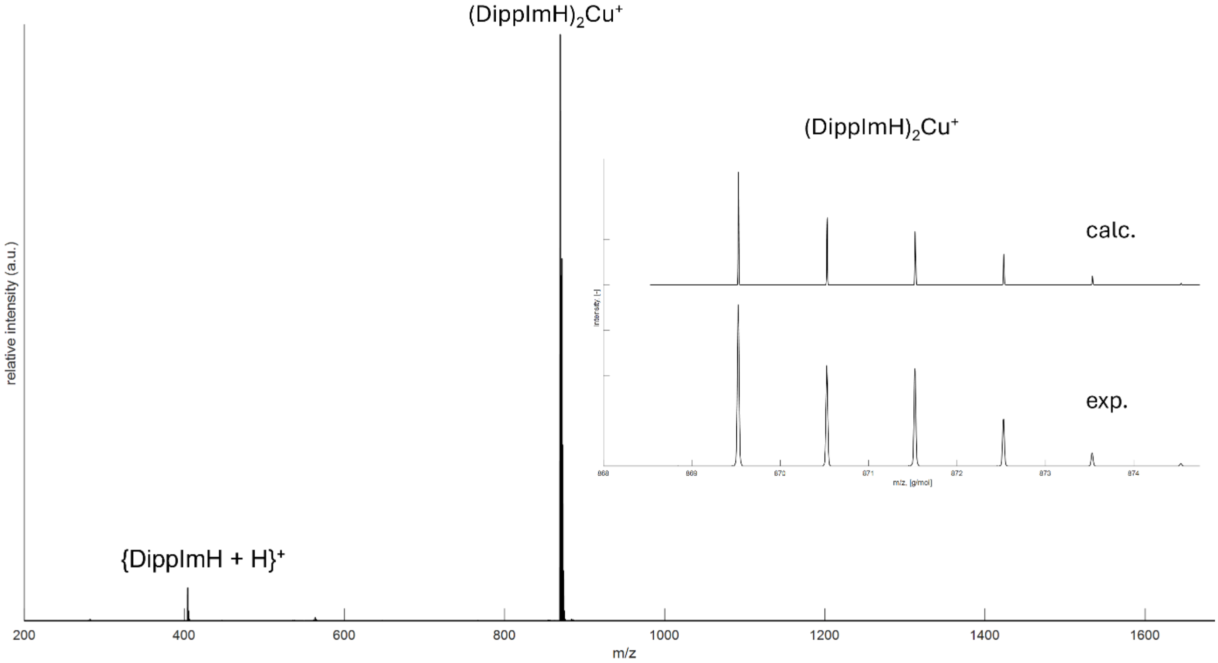

Figure S29: ESI-MS (positive) spectrum of [Cu(DippImH)$_2$][BAr^F] (**3**).

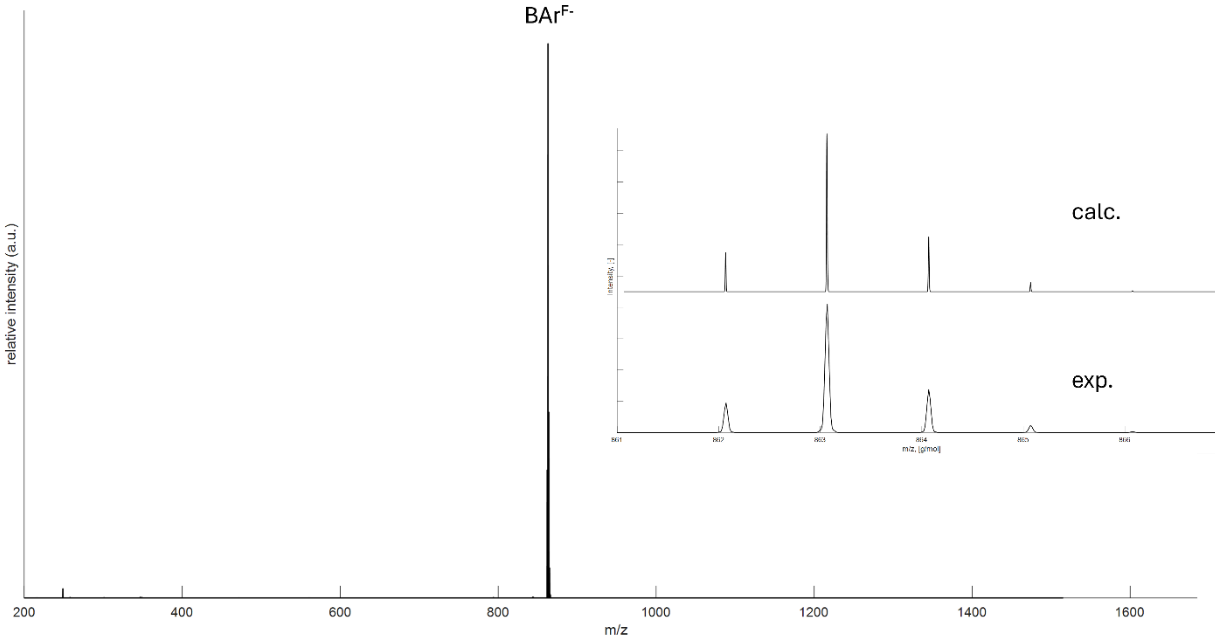

Figure S30: ESI-MS (negative) spectrum of $[Cu(DippImH)_2][BAr^F]$ (**3**).

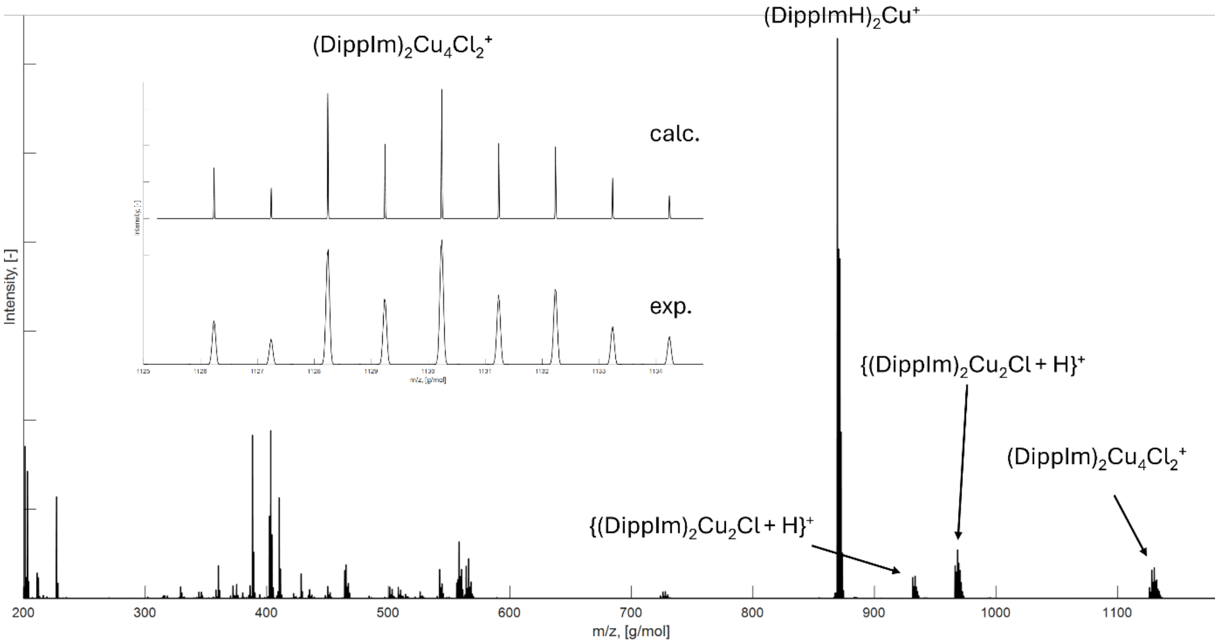

Figure S31: LIFDI-MS (positive) spectrum of the sample, from which $[Cu_3(DippIm)_2Cl]$ (**4**) was crystallized.

4. Crystallography

SC-XRD data were collected on a Bruker D8-Venture single crystal x-ray diffractometer equipped with either a TXS rotating anode with MoK_α radiation (λ = 0.71073 Å), a Bruker Photon III detector and a Helios optic (compounds **2**, **4** and **5**) or equipped with an IMS microsource with MoK_α radiation (λ = 0.71073 Å), a Bruker Photon II detector (compounds **1** and **3**), using the Bruker APEX4 software package.[C1] Single crystals were coated with perfluorinated ether, fixed on top of a micro sampler and frozen under a stream of cold

nitrogen. A matrix scan was used to determine the initial lattice parameters. All data were integrated with the Bruker SAINT V8.40B software package using a narrow-frame algorithm and the reflections were corrected for Lorentz and polarisation effects, scan speed, and background.[C2] Data were corrected for absorption effects including odd and even ordered spherical harmonics by the multi-scan method (SADABS 2016/2).[C3] Space group assignment was based upon systematic absences, E statistics, and successful refinement of the structure.

The structures were solved by direct methods using SHELXT and refined by full-matrix least-squares methods against F^2 by minimizing $\Sigma w(F_o^2 - F_c^2)^2$ using SHELXL in conjunction with SHELXLE.[C4-C6] All non-hydrogen atoms were refined with anisotropic displacement parameters. Hydrogen atoms were refined isotropically on calculated positions using a riding model with their U_{iso} values constrained to 1.5 times the U_{eq} of their pivot atoms for terminal sp^3 carbon atoms and a C–H distance of 0.98 Å. Non-methyl hydrogen atoms were refined using a riding model with methylene, aromatic, and other C–H distances of 0.99 Å, 0.95 Å, and 1.00 Å, respectively, and U_{iso} values constrained to 1.2 times the U_{eq} of their pivot atoms. A split layer refinement was used for disordered groups and additional restraints on distances, angles and anisotropic displacement parameters were employed to ensure convergence within chemically reasonable limits, if necessary. Severe disorder of e.g. CF_3 groups was treated using the DSR plugin within SHELXLE.[C7] Compound **2** was refined as a two-component inversion twin.

Neutral atom scattering factors for all atoms and anomalous dispersion corrections for the non-hydrogen atoms were taken from International Tables for Crystallography.[C8] Crystallographic data for the structures reported in this paper have been deposited with the Cambridge Crystallographic Data Centre.[C9] Supplementary crystallographic data reported in this paper have been deposited with the Cambridge Crystallographic Data Centre (CCDC 2363593-2363597) and can be obtained free of charge from The Cambridge Crystallographic Data Centre via www.ccdc.cam.ac.uk/structures. All CIF files were generated using FinalCif.[C10] Images of the crystal structures were generated with *Mercury* and *Povray*.[C11]

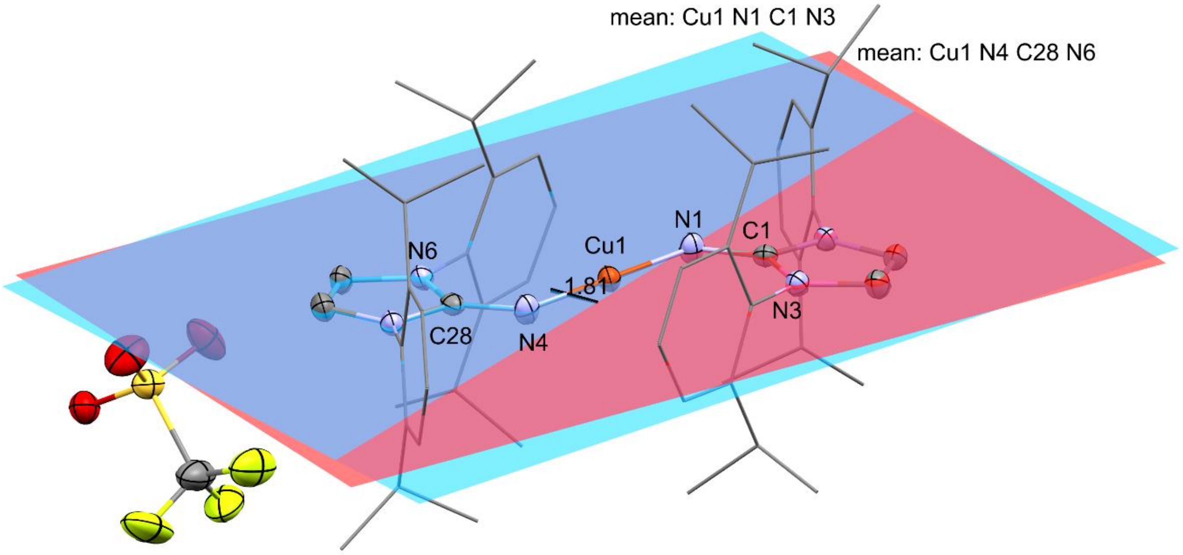

Figure S32: Angle between the two plains containing ligands in compound **2**.

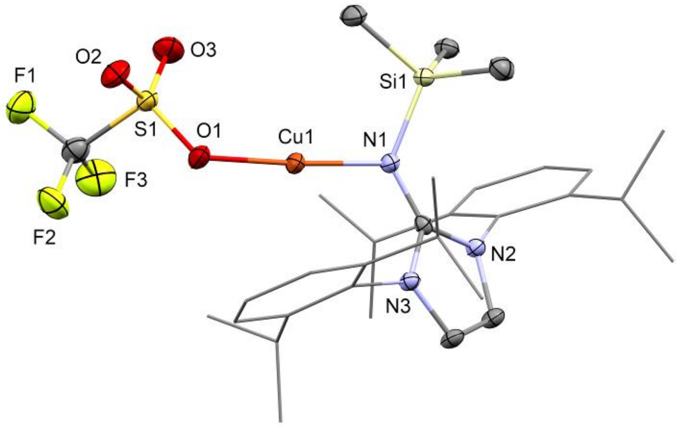

Table S1: Crystal data and structure refinement for [Cu(DippImTMS)(OTf)] (**1**).

CCDC number	2363593
Empirical formula	$C_{31}H_{45}CuF_3N_3O_3SSi$
Formula weight	688.39
Temperature [K]	100(2)
Crystal system	monoclinic
Space group (number)	$P2_1/n$ (14)
a [Å]	10.0367(4)
b [Å]	20.8306(9)
c [Å]	16.5295(7)
α [°]	90
β [°]	96.4400(10)

γ [°]	90
Volume [Å^3]	3434.0(2)
Z	4
ρ_{calc} [gcm^{-3}]	1.331
μ [mm^{-1}]	0.782
F(000)	1448
Crystal size [mm^3]	0.274×0.551×0.940
Crystal colour	colourless
Crystal shape	block
Radiation	MoK_α (λ=0.71073 Å)
2θ range [°]	4.53 to 50.05 (0.84 Å)
Index ranges	−11 ≤ h ≤ 11<br>−24 ≤ k ≤ 24<br>−19 ≤ l ≤ 19
Reflections collected	74153
Independent reflections	6039<br>R_{int} = 0.0422<br>R_{sigma} = 0.0165
Completeness to<br>θ = 25.026°	99.7 %
Data / Restraints / Parameters	6039 / 0 / 399
Goodness-of-fit on F^2	1.046
Final *R* indexes<br>[I≥2σ(I)]	R_1 = 0.0254<br>wR_2 = 0.0598
Final *R* indexes<br>[all data]	R_1 = 0.0291<br>wR_2 = 0.0625
Largest peak/hole [eÅ^{-3}]	0.29/−0.30

Table S2: Atomic coordinates and Ueq [Å^2] for compound **1**.

**Atom**	***x***	***y***	***z***	U_{eq}
Cu1	0.28518(2)	0.72471(2)	0.32187(2)	0.01848(6)
S1	0.15473(4)	0.82573(2)	0.41967(2)	0.02126(10)
Si1	0.37468(4)	0.59557(2)	0.38984(3)	0.01472(10)
F1	0.18765(15)	0.94112(6)	0.47827(7)	0.0499(3)
F2	0.16357(12)	0.93775(5)	0.34733(7)	0.0402(3)
F3	0.34873(11)	0.90918(6)	0.41315(8)	0.0459(3)
O1	0.18734(13)	0.79878(6)	0.34195(7)	0.0273(3)
O2	0.01368(12)	0.83384(6)	0.42148(8)	0.0302(3)
O3	0.23005(14)	0.79970(7)	0.49069(8)	0.0357(3)
N1	0.38962(13)	0.65066(6)	0.31150(8)	0.0147(3)
N2	0.56207(12)	0.61407(6)	0.22882(8)	0.0138(3)
N3	0.39165(13)	0.66649(6)	0.16834(8)	0.0145(3)
C1	0.44407(15)	0.64313(7)	0.24273(9)	0.0132(3)
C2	0.58052(16)	0.62007(8)	0.14643(9)	0.0181(3)
H2	0.654296	0.603979	0.121278	0.022
C3	0.47628(16)	0.65234(8)	0.10958(10)	0.0179(3)
H3	0.462304	0.663611	0.053581	0.021
C4	0.67173(15)	0.59484(8)	0.28818(9)	0.0147(3)
C5	0.74350(15)	0.64321(8)	0.33377(10)	0.0173(3)
C6	0.85576(17)	0.62380(9)	0.38550(10)	0.0234(4)
H6	0.906539	0.654998	0.417572	0.028
C7	0.89465(17)	0.56013(9)	0.39106(11)	0.0264(4)
H7	0.971400	0.548100	0.426766	0.032

C8	0.82237(17)	0.51388(8)	0.34491(10)	0.0229(4)
H8	0.850364	0.470328	0.349201	0.027
C9	0.70905(15)	0.53011(8)	0.29216(10)	0.0172(3)
C10	0.70791(17)	0.71388(8)	0.32508(10)	0.0205(4)
H10	0.612172	0.716621	0.300928	0.025
C11	0.7197(2)	0.74991(9)	0.40621(12)	0.0321(4)
H11A	0.685328	0.793666	0.397314	0.048
H11B	0.814060	0.751610	0.429137	0.048
H11C	0.667432	0.727532	0.444167	0.048
C12	0.7929(2)	0.74591(9)	0.26544(12)	0.0332(4)
H12A	0.767111	0.791130	0.258717	0.050
H12B	0.778296	0.724099	0.212657	0.050
H12C	0.887895	0.743041	0.286685	0.050
C13	0.63284(16)	0.47835(8)	0.24088(10)	0.0190(3)
H13	0.545124	0.496990	0.217391	0.023
C14	0.60321(18)	0.41994(8)	0.29215(11)	0.0257(4)
H14A	0.546472	0.389681	0.258478	0.039
H14B	0.556583	0.433872	0.338128	0.039
H14C	0.687594	0.398888	0.312683	0.039
C15	0.70925(17)	0.45740(9)	0.16977(11)	0.0253(4)
H15A	0.658090	0.424032	0.138103	0.038
H15B	0.797237	0.440392	0.191165	0.038
H15C	0.721137	0.494414	0.134695	0.038
C16	0.27561(15)	0.70750(8)	0.15380(9)	0.0154(3)
C17	0.29624(17)	0.77412(8)	0.15523(9)	0.0184(3)
C18	0.18361(18)	0.81279(8)	0.13740(10)	0.0232(4)
H18	0.193253	0.858160	0.138456	0.028
C19	0.05794(18)	0.78608(9)	0.11819(10)	0.0245(4)
H19	−0.017583	0.813327	0.106108	0.029
C20	0.04079(17)	0.72016(9)	0.11633(10)	0.0226(4)
H20	−0.046204	0.702698	0.102388	0.027
C21	0.14961(16)	0.67905(8)	0.13467(9)	0.0181(3)
C22	0.43558(17)	0.80346(8)	0.17233(10)	0.0228(4)
H22	0.497716	0.768822	0.195251	0.027
C23	0.4423(2)	0.85763(11)	0.23433(13)	0.0402(5)
H23A	0.534588	0.873536	0.244252	0.060
H23B	0.413702	0.841654	0.285406	0.060
H23C	0.382970	0.892620	0.213281	0.060
C24	0.4862(2)	0.82706(11)	0.09351(12)	0.0367(5)
H24A	0.578635	0.842455	0.105268	0.055
H24B	0.428983	0.862172	0.070521	0.055
H24C	0.483459	0.791658	0.054312	0.055
C25	0.13318(17)	0.60673(8)	0.12894(11)	0.0254(4)
H25	0.210488	0.586631	0.163242	0.031
C26	0.0044(2)	0.58310(11)	0.15983(16)	0.0462(6)
H26A	0.002930	0.536063	0.159417	0.069
H26B	−0.073026	0.599467	0.124433	0.069
H26C	0.000470	0.598584	0.215495	0.069
C27	0.1395(2)	0.58558(10)	0.04019(13)	0.0407(5)
H27A	0.124320	0.539179	0.035598	0.061
H27B	0.227925	0.595934	0.023938	0.061
H27C	0.070160	0.608159	0.004540	0.061

C28	0.25017(17)	0.62952(9)	0.45387(10)	0.0239(4)
H28A	0.236099	0.599179	0.497428	0.036
H28B	0.165005	0.636984	0.420034	0.036
H28C	0.283975	0.670221	0.477799	0.036
C29	0.30533(18)	0.51904(8)	0.34411(11)	0.0243(4)
H29A	0.299427	0.487226	0.387242	0.036
H29B	0.364367	0.502858	0.305350	0.036
H29C	0.215742	0.526824	0.315711	0.036
C30	0.53087(16)	0.58119(8)	0.45982(10)	0.0199(3)
H30A	0.508153	0.560256	0.509564	0.030
H30B	0.575172	0.622281	0.473772	0.030
H30C	0.591365	0.553489	0.432891	0.030
C31	0.21704(18)	0.90772(9)	0.41375(11)	0.0278(4)

U_{eq} is defined as 1/3 of the trace of the orthogonalized U_{ij} tensor.

Table S3: Bond lengths and angles for compound **1**.

**Atom–Atom**	**Length [Å]**
Cu1–O1	1.8784(12)
Cu1–N1	1.8835(13)
S1–O2	1.4294(13)
S1–O3	1.4299(13)
S1–O1	1.4722(12)
S1–C31	1.8250(19)
Si1–N1	1.7493(13)
Si1–C30	1.8650(16)
Si1–C28	1.8651(17)
Si1–C29	1.8653(17)
F1–C31	1.334(2)
F2–C31	1.324(2)
F3–C31	1.323(2)
N1–C1	1.325(2)
N2–C1	1.372(2)
N2–C2	1.400(2)
N2–C4	1.4465(19)
N3–C1	1.3714(19)
N3–C3	1.392(2)
N3–C16	1.4426(19)
C2–C3	1.332(2)
C2–H2	0.9500
C3–H3	0.9500
C4–C9	1.399(2)
C4–C5	1.407(2)
C5–C6	1.396(2)
C5–C10	1.518(2)
C6–C7	1.383(3)
C6–H6	0.9500
C7–C8	1.383(2)
C7–H7	0.9500
C8–C9	1.395(2)
C8–H8	0.9500
C9–C13	1.523(2)
C10–C12	1.528(2)
C10–C11	1.530(2)

C10–H10	1.0000
C11–H11A	0.9800
C11–H11B	0.9800
C11–H11C	0.9800
C12–H12A	0.9800
C12–H12B	0.9800
C12–H12C	0.9800
C13–C14	1.531(2)
C13–C15	1.537(2)
C13–H13	1.0000
C14–H14A	0.9800
C14–H14B	0.9800
C14–H14C	0.9800
C15–H15A	0.9800
C15–H15B	0.9800
C15–H15C	0.9800
C16–C21	1.400(2)
C16–C17	1.403(2)
C17–C18	1.392(2)
C17–C22	1.523(2)
C18–C19	1.383(3)
C18–H18	0.9500
C19–C20	1.384(3)
C19–H19	0.9500
C20–C21	1.394(2)
C20–H20	0.9500
C21–C25	1.517(2)
C22–C23	1.521(3)
C22–C24	1.532(2)
C22–H22	1.0000
C23–H23A	0.9800
C23–H23B	0.9800
C23–H23C	0.9800
C24–H24A	0.9800
C24–H24B	0.9800
C24–H24C	0.9800
C25–C26	1.524(3)
C25–C27	1.540(3)
C25–H25	1.0000
C26–H26A	0.9800
C26–H26B	0.9800
C26–H26C	0.9800
C27–H27A	0.9800
C27–H27B	0.9800
C27–H27C	0.9800
C28–H28A	0.9800
C28–H28B	0.9800
C28–H28C	0.9800
C29–H29A	0.9800
C29–H29B	0.9800
C29–H29C	0.9800
C30–H30A	0.9800
C30–H30B	0.9800
C30–H30C	0.9800

**Atom–Atom–Atom**	**Angle [°]**
O1–Cu1–N1	174.93(5)
O2–S1–O3	117.47(8)
O2–S1–O1	112.39(8)
O3–S1–O1	115.02(8)
O2–S1–C31	103.70(8)
O3–S1–C31	104.25(9)
O1–S1–C31	101.48(8)
N1–Si1–C30	115.38(7)
N1–Si1–C28	106.85(7)
C30–Si1–C28	106.20(8)
N1–Si1–C29	108.65(7)
C30–Si1–C29	111.32(8)
C28–Si1–C29	108.10(8)
S1–O1–Cu1	129.74(8)
C1–N1–Si1	129.45(11)
C1–N1–Cu1	117.55(10)
Si1–N1–Cu1	112.03(7)
C1–N2–C2	109.53(12)
C1–N2–C4	127.87(13)
C2–N2–C4	120.72(13)
C1–N3–C3	110.35(13)
C1–N3–C16	125.51(13)
C3–N3–C16	123.63(13)
N1–C1–N3	125.01(14)
N1–C1–N2	130.01(14)
N3–C1–N2	104.94(13)
C3–C2–N2	107.87(14)
C3–C2–H2	126.1
N2–C2–H2	126.1
C2–C3–N3	107.30(14)
C2–C3–H3	126.4
N3–C3–H3	126.4
C9–C4–C5	123.14(14)
C9–C4–N2	118.56(14)
C5–C4–N2	118.03(14)
C6–C5–C4	116.75(15)
C6–C5–C10	120.28(15)
C4–C5–C10	122.88(14)
C7–C6–C5	121.41(16)
C7–C6–H6	119.3
C5–C6–H6	119.3
C6–C7–C8	120.33(15)
C6–C7–H7	119.8
C8–C7–H7	119.8
C7–C8–C9	121.09(16)
C7–C8–H8	119.5
C9–C8–H8	119.5
C8–C9–C4	117.28(15)
C8–C9–C13	119.96(14)
C4–C9–C13	122.75(14)
C5–C10–C12	110.01(14)
C5–C10–C11	113.49(15)
C12–C10–C11	110.94(14)
C5–C10–H10	107.4

C12–C10–H10	107.4
C11–C10–H10	107.4
C10–C11–H11A	109.5
C10–C11–H11B	109.5
H11A–C11–H11B	109.5
C10–C11–H11C	109.5
H11A–C11–H11C	109.5
H11B–C11–H11C	109.5
C10–C12–H12A	109.5
C10–C12–H12B	109.5
H12A–C12–H12B	109.5
C10–C12–H12C	109.5
H12A–C12–H12C	109.5
H12B–C12–H12C	109.5
C9–C13–C14	111.78(14)
C9–C13–C15	111.35(13)
C14–C13–C15	110.02(14)
C9–C13–H13	107.8
C14–C13–H13	107.8
C15–C13–H13	107.8
C13–C14–H14A	109.5
C13–C14–H14B	109.5
H14A–C14–H14B	109.5
C13–C14–H14C	109.5
H14A–C14–H14C	109.5
H14B–C14–H14C	109.5
C13–C15–H15A	109.5
C13–C15–H15B	109.5
H15A–C15–H15B	109.5
C13–C15–H15C	109.5
H15A–C15–H15C	109.5
H15B–C15–H15C	109.5
C21–C16–C17	123.39(15)
C21–C16–N3	118.61(14)
C17–C16–N3	117.91(14)
C18–C17–C16	116.99(15)
C18–C17–C22	120.92(15)
C16–C17–C22	122.05(15)
C19–C18–C17	120.93(16)
C19–C18–H18	119.5
C17–C18–H18	119.5
C18–C19–C20	120.82(16)
C18–C19–H19	119.6
C20–C19–H19	119.6
C19–C20–C21	120.81(16)
C19–C20–H20	119.6
C21–C20–H20	119.6
C20–C21–C16	117.06(15)
C20–C21–C25	121.28(15)
C16–C21–C25	121.56(14)
C23–C22–C17	113.16(15)
C23–C22–C24	109.94(16)
C17–C22–C24	110.87(14)
C23–C22–H22	107.5
C17–C22–H22	107.5

C24–C22–H22	107.5
C22–C23–H23A	109.5
C22–C23–H23B	109.5
H23A–C23–H23B	109.5
C22–C23–H23C	109.5
H23A–C23–H23C	109.5
H23B–C23–H23C	109.5
C22–C24–H24A	109.5
C22–C24–H24B	109.5
H24A–C24–H24B	109.5
C22–C24–H24C	109.5
H24A–C24–H24C	109.5
H24B–C24–H24C	109.5
C21–C25–C26	112.96(16)
C21–C25–C27	109.13(15)
C26–C25–C27	110.72(17)
C21–C25–H25	108.0
C26–C25–H25	108.0
C27–C25–H25	108.0
C25–C26–H26A	109.5
C25–C26–H26B	109.5
H26A–C26–H26B	109.5
C25–C26–H26C	109.5
H26A–C26–H26C	109.5
H26B–C26–H26C	109.5
C25–C27–H27A	109.5
C25–C27–H27B	109.5
H27A–C27–H27B	109.5
C25–C27–H27C	109.5
H27A–C27–H27C	109.5
H27B–C27–H27C	109.5
Si1–C28–H28A	109.5
Si1–C28–H28B	109.5
H28A–C28–H28B	109.5
Si1–C28–H28C	109.5
H28A–C28–H28C	109.5
H28B–C28–H28C	109.5
Si1–C29–H29A	109.5
Si1–C29–H29B	109.5
H29A–C29–H29B	109.5
Si1–C29–H29C	109.5
H29A–C29–H29C	109.5
H29B–C29–H29C	109.5
Si1–C30–H30A	109.5
Si1–C30–H30B	109.5
H30A–C30–H30B	109.5
Si1–C30–H30C	109.5
H30A–C30–H30C	109.5
H30B–C30–H30C	109.5
F3–C31–F2	107.18(15)
F3–C31–F1	107.73(15)
F2–C31–F1	108.12(16)
F3–C31–S1	111.75(13)
F2–C31–S1	112.16(12)
F1–C31–S1	109.72(13)

Table S4: Torsion angles for compound **1**.

Atom–Atom–Atom–Atom	Torsion Angle [°]
O2–S1–O1–Cu1	−123.83(11)
O3–S1–O1–Cu1	14.15(15)
C31–S1–O1–Cu1	125.99(11)
C30–Si1–N1–C1	76.90(15)
C28–Si1–N1–C1	−165.30(14)
C29–Si1–N1–C1	−48.89(15)
C30–Si1–N1–Cu1	−114.85(8)
C28–Si1–N1–Cu1	2.95(9)
C29–Si1–N1–Cu1	119.36(8)
Si1–N1–C1–N3	138.81(13)
Cu1–N1–C1–N3	−28.90(19)
Si1–N1–C1–N2	−43.8(2)
Cu1–N1–C1–N2	148.46(13)
C3–N3–C1–N1	177.61(14)
C16–N3–C1–N1	5.6(2)
C3–N3–C1–N2	−0.29(16)
C16–N3–C1–N2	−172.29(13)
C2–N2–C1–N1	−177.65(15)
C4–N2–C1–N1	−13.4(3)
C2–N2–C1–N3	0.11(16)
C4–N2–C1–N3	164.35(14)
C1–N2–C2–C3	0.12(18)
C4–N2–C2–C3	−165.44(14)
N2–C2–C3–N3	−0.29(18)
C1–N3–C3–C2	0.37(18)
C16–N3–C3–C2	172.55(14)
C1–N2–C4–C9	120.83(17)
C2–N2–C4–C9	−76.49(19)
C1–N2–C4–C5	−65.0(2)
C2–N2–C4–C5	97.67(17)
C9–C4–C5–C6	−0.8(2)
N2–C4–C5–C6	−174.66(14)
C9–C4–C5–C10	175.85(15)
N2–C4–C5–C10	2.0(2)
C4–C5–C6–C7	0.4(2)
C10–C5–C6–C7	−176.33(16)
C5–C6–C7–C8	0.1(3)
C6–C7–C8–C9	−0.2(3)
C7–C8–C9–C4	−0.2(3)
C7–C8–C9–C13	178.87(16)
C5–C4–C9–C8	0.7(2)
N2–C4–C9–C8	174.52(14)
C5–C4–C9–C13	−178.33(15)
N2–C4–C9–C13	−4.5(2)
C6–C5–C10–C12	81.38(19)
C4–C5–C10–C12	−95.15(18)
C6–C5–C10–C11	−43.6(2)
C4–C5–C10–C11	139.90(16)
C8–C9–C13–C14	48.9(2)
C4–C9–C13–C14	−132.10(16)
C8–C9–C13–C15	−74.59(19)
C4–C9–C13–C15	104.39(18)
C1–N3–C16–C21	−91.62(18)
C3–N3–C16–C21	97.39(18)
C1–N3–C16–C17	91.84(18)
C3–N3–C16–C17	−79.15(19)
C21–C16–C17–C18	0.8(2)
N3–C16–C17–C18	177.16(14)
C21–C16–C17–C22	−177.02(15)
N3–C16–C17–C22	−0.7(2)
C16–C17–C18–C19	−0.8(2)
C22–C17–C18–C19	176.99(15)
C17–C18–C19–C20	0.1(3)
C18–C19–C20–C21	0.7(3)
C19–C20–C21–C16	−0.8(2)
C19–C20–C21–C25	−177.06(16)
C17–C16–C21–C20	0.0(2)
N3–C16–C21–C20	−176.34(14)
C17–C16–C21–C25	176.28(15)
N3–C16–C21–C25	−0.1(2)
C18–C17–C22–C23	49.2(2)
C16–C17–C22–C23	−133.05(17)
C18–C17–C22–C24	−74.8(2)
C16–C17–C22–C24	102.89(19)
C20–C21–C25–C26	−39.8(2)
C16–C21–C25–C26	144.09(17)
C20–C21–C25–C27	83.8(2)
C16–C21–C25–C27	−92.30(19)
O2–S1–C31–F3	178.75(12)
O3–S1–C31–F3	55.26(14)
O1–S1–C31–F3	−64.53(14)
O2–S1–C31–F2	−60.86(15)
O3–S1–C31–F2	175.65(13)
O1–S1–C31–F2	55.86(14)
O2–S1–C31–F1	59.32(14)
O3–S1–C31–F1	−64.17(14)
O1–S1–C31–F1	176.04(12)

Table S5: Hydrogen bonds for compound **1**.

**D–H···A [Å]**	**d(D–H) [Å]**	**d(H···A) [Å]**	**d(D···A) [Å]**	**<(DHA) [°]**
C2–H2···F1$^{\#1}$	0.95	2.60	3.343(2)	135.4
C3–H3···O2$^{\#1}$	0.95	2.30	3.186(2)	155.0

Symmetry transformations used to generate equivalent atoms:
#1: 0.5+X, 1.5-Y, -0.5+Z;

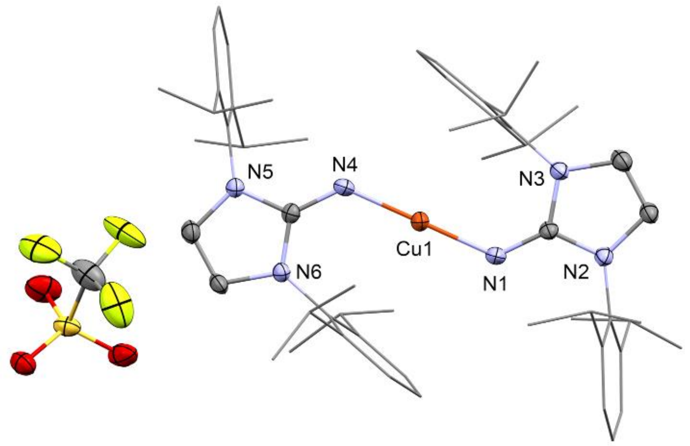

Table S6: Crystal data and structure refinement for [Cu(DippImH)$_2$][OTf] (**2**).

CCDC number	2363594
Empirical formula	$C_{67}H_{84}CuF_5N_6O_3S$
Formula weight	1212.00
Temperature [K]	100(2)
Crystal system	monoclinic
Space group (number)	$P2_1$ (4)
a [Å]	10.7222(7)
b [Å]	21.1889(14)
c [Å]	14.2981(10)
α [°]	90
β [°]	90.948(2)
γ [°]	90
Volume [Å^3]	3248.0(4)
Z	2
ρ_{calc} [gcm^{-3}]	1.239
μ [mm^{-1}]	0.432
F(000)	1284
Crystal size [mm^3]	0.085×0.128×0.139
Crystal colour	white
Crystal shape	plate
Radiation	MoK_α (λ=0.71073 Å)
2θ range [°]	4.71 to 50.05 (0.84 Å)
Index ranges	−12 ≤ h ≤ 12<br>−25 ≤ k ≤ 25<br>−17 ≤ l ≤ 17
Reflections collected	151265
Independent reflections	11452<br>R_{int} = 0.0631<br>R_{sigma} = 0.0276
Completeness to θ = 25.028°	99.9 %
Data / Restraints / Parameters	11452 / 623 / 852
Goodness-of-fit on F^2	1.045
Final *R* indexes [I≥2σ(I)]	R_1 = 0.0322<br>wR_2 = 0.0769
Final *R* indexes [all data]	R_1 = 0.0380<br>wR_2 = 0.0806
Largest peak/hole [eÅ^{-3}]	0.47/−0.28
Flack X parameter	0.061(10)

Table S7: Atomic coordinates and Ueq [Å^2] for compound **2**.

Atom	*x*	*y*	*z*	U_{eq}
Cu1	0.63314(4)	0.51962(2)	0.49029(3)	0.01974(10)
S1	0.30502(8)	0.29198(4)	0.08361(7)	0.0283(2)
F1	0.1114(3)	0.36212(15)	0.0430(3)	0.0786(10)
F2	0.2398(3)	0.40528(13)	0.1393(3)	0.0794(11)
F3	0.1210(3)	0.33100(17)	0.1839(2)	0.0777(10)
F4	0.3012(3)	0.60525(14)	0.7985(3)	0.0726(9)
O1	0.3751(3)	0.32216(15)	0.0115(2)	0.0492(8)
O2	0.2300(2)	0.23927(12)	0.05364(19)	0.0330(6)
O3	0.3740(3)	0.28222(13)	0.1690(2)	0.0457(8)
N1	0.6779(3)	0.51077(14)	0.61471(19)	0.0232(6)
H1	0.690618	0.471814	0.633959	0.028
N2	0.7158(3)	0.54715(13)	0.7713(2)	0.0199(6)
N3	0.6893(3)	0.61834(13)	0.6623(2)	0.0210(6)
N4	0.5879(3)	0.52832(14)	0.36599(19)	0.0223(6)
H4	0.578916	0.567284	0.345676	0.027
N5	0.5409(3)	0.49035(13)	0.21109(19)	0.0194(6)
N6	0.5703(3)	0.42066(13)	0.32114(19)	0.0189(6)
C1	0.6933(3)	0.55455(15)	0.6779(2)	0.0188(7)
C2	0.7244(3)	0.60668(16)	0.8143(3)	0.0234(8)
H2	0.739138	0.614705	0.878881	0.028
C3	0.7080(3)	0.64992(17)	0.7470(3)	0.0269(8)
H3	0.708947	0.694394	0.755470	0.032
C4	0.7267(3)	0.48555(17)	0.8130(2)	0.0210(7)
C5	0.8451(3)	0.45762(17)	0.8203(2)	0.0240(8)
C6	0.8507(4)	0.39604(18)	0.8534(3)	0.0312(9)
H6	0.929135	0.375396	0.858569	0.037
C7	0.7444(4)	0.3645(2)	0.8789(3)	0.0361(9)
H7	0.750467	0.322140	0.900510	0.043
C8	0.6284(4)	0.39352(19)	0.8737(3)	0.0328(9)
H8	0.556303	0.371389	0.893082	0.039
C9	0.6174(3)	0.45511(18)	0.8399(2)	0.0260(8)
C10	0.9621(3)	0.49278(18)	0.7915(3)	0.0289(8)
H10	0.942133	0.538878	0.791006	0.035
C11	1.0005(4)	0.4745(2)	0.6932(3)	0.0386(10)
H11A	0.933319	0.485353	0.648677	0.058
H11B	1.016423	0.429034	0.690685	0.058
H11C	1.076410	0.497484	0.676780	0.058
C12	1.0710(4)	0.4826(2)	0.8607(3)	0.0431(11)
H12A	1.097723	0.438373	0.858448	0.065
H12B	1.044179	0.492855	0.924094	0.065
H12C	1.140756	0.509964	0.843800	0.065
C13	0.4896(3)	0.48606(19)	0.8313(2)	0.0275(8)
H13	0.501717	0.529803	0.806776	0.033
C14	0.4056(4)	0.4507(3)	0.7619(3)	0.0490(12)
H14A	0.326966	0.473769	0.753503	0.074
H14B	0.388483	0.408370	0.786000	0.074
H14C	0.447410	0.447380	0.701683	0.074
C15	0.4289(4)	0.4912(2)	0.9264(3)	0.0423(11)
H15A	0.351818	0.515900	0.920442	0.063

H15B	0.486269	0.512241	0.970434	0.063
H15C	0.409376	0.448909	0.949604	0.063
C16	0.6720(3)	0.64527(15)	0.5702(2)	0.0220(7)
C17	0.5519(3)	0.66229(16)	0.5409(3)	0.0242(8)
C18	0.5369(4)	0.68565(17)	0.4500(3)	0.0296(9)
H18	0.456381	0.697794	0.427924	0.036
C19	0.6377(4)	0.69129(18)	0.3918(3)	0.0302(9)
H19	0.625817	0.706484	0.329819	0.036
C20	0.7563(4)	0.67493(17)	0.4234(3)	0.0282(8)
H20	0.825152	0.680099	0.383154	0.034
C21	0.7760(3)	0.65113(17)	0.5128(3)	0.0249(8)
C22	0.4394(3)	0.65583(17)	0.6042(3)	0.0280(8)
H22	0.469303	0.636743	0.664446	0.034
C23	0.3427(5)	0.6125(3)	0.5627(5)	0.088(2)
H23A	0.309035	0.630886	0.504754	0.131
H23B	0.275196	0.606701	0.607286	0.131
H23C	0.380841	0.571529	0.549132	0.131
C24	0.3858(6)	0.7195(2)	0.6261(4)	0.0633(16)
H24A	0.359373	0.740313	0.567805	0.095
H24B	0.449371	0.745342	0.657907	0.095
H24C	0.313849	0.714480	0.666723	0.095
C25	0.9065(3)	0.63418(18)	0.5482(3)	0.0288(8)
H25	0.897495	0.601542	0.598193	0.035
C26	0.9881(4)	0.6061(2)	0.4723(3)	0.0408(10)
H26A	0.942197	0.572537	0.439572	0.061
H26B	1.064226	0.588624	0.500980	0.061
H26C	1.010315	0.639188	0.427659	0.061
C27	0.9692(4)	0.6918(2)	0.5928(3)	0.0431(11)
H27A	0.981425	0.724240	0.544970	0.065
H27B	1.050235	0.679486	0.619762	0.065
H27C	0.916221	0.708538	0.642118	0.065
C28	0.5674(3)	0.48357(16)	0.3039(2)	0.0182(7)
C29	0.5284(3)	0.43051(17)	0.1706(3)	0.0256(8)
H29	0.511090	0.421928	0.106449	0.031
C30	0.5452(3)	0.38787(17)	0.2376(2)	0.0243(8)
H30	0.541064	0.343348	0.230401	0.029
C31	0.5434(3)	0.55050(17)	0.1653(2)	0.0226(8)
C32	0.4313(3)	0.58395(17)	0.1558(3)	0.0266(8)
C33	0.4387(4)	0.6435(2)	0.1143(3)	0.0393(10)
H33	0.364980	0.667887	0.106030	0.047
C34	0.5511(4)	0.6675(2)	0.0850(3)	0.0419(11)
H34	0.553839	0.708299	0.057404	0.050
C35	0.6589(4)	0.6333(2)	0.0952(3)	0.0373(10)
H35	0.735307	0.650502	0.074208	0.045
C36	0.6580(3)	0.57381(18)	0.1359(3)	0.0278(8)
C37	0.3085(4)	0.5581(2)	0.1893(3)	0.0352(9)
H37	0.320797	0.512256	0.203550	0.042
C38	0.2056(4)	0.5633(3)	0.1144(3)	0.0483(12)
H38A	0.233406	0.543380	0.056582	0.072
H38B	0.187159	0.607954	0.102566	0.072
H38C	0.130286	0.542082	0.136207	0.072
C39	0.2701(4)	0.5908(3)	0.2796(3)	0.0505(12)

H39A	0.264491	0.636416	0.268935	0.076
H39B	0.332324	0.582232	0.328879	0.076
H39C	0.188666	0.574762	0.298770	0.076
C40	0.7777(3)	0.5357(2)	0.1489(3)	0.0331(9)
H40	0.753905	0.493021	0.172443	0.040
C41	0.8459(4)	0.5264(4)	0.0570(3)	0.0605(14)
H41A	0.790059	0.505931	0.011352	0.091
H41B	0.919589	0.499895	0.067868	0.091
H41C	0.871957	0.567566	0.032706	0.091
C42	0.8627(4)	0.5663(2)	0.2232(3)	0.0415(11)
H42A	0.937016	0.540094	0.233138	0.062
H42B	0.817636	0.570105	0.282027	0.062
H42C	0.887822	0.608362	0.202004	0.062
C43	0.5932(3)	0.39330(15)	0.4119(2)	0.0197(7)
C44	0.7166(3)	0.38416(16)	0.4414(2)	0.0222(7)
C45	0.7365(3)	0.35866(17)	0.5301(3)	0.0259(8)
H45	0.819365	0.351915	0.552440	0.031
C46	0.6372(4)	0.34301(17)	0.5862(3)	0.0263(8)
H46	0.652507	0.325928	0.646774	0.032
C47	0.5158(3)	0.35203(17)	0.5549(3)	0.0254(8)
H47	0.448518	0.340464	0.593780	0.031
C48	0.4908(3)	0.37799(16)	0.4666(2)	0.0223(7)
C49	0.8242(3)	0.40004(18)	0.3777(3)	0.0286(8)
H49	0.796348	0.435607	0.336366	0.034
C50	0.8539(4)	0.3445(2)	0.3148(4)	0.0489(12)
H50A	0.778152	0.331391	0.280825	0.073
H50B	0.885168	0.309317	0.352997	0.073
H50C	0.917610	0.356969	0.270039	0.073
C51	0.9411(4)	0.4220(2)	0.4306(3)	0.0465(12)
H51A	1.003273	0.436388	0.385916	0.070
H51B	0.975389	0.386888	0.467481	0.070
H51C	0.919588	0.456834	0.472469	0.070
C52	0.3567(3)	0.38936(17)	0.4336(3)	0.0279(8)
H52	0.359190	0.412127	0.372427	0.033
C53	0.2877(4)	0.3282(2)	0.4183(4)	0.0436(11)
H53A	0.203432	0.337137	0.394287	0.065
H53B	0.282497	0.305422	0.477755	0.065
H53C	0.332368	0.302359	0.372976	0.065
C54	0.2877(4)	0.4311(3)	0.5028(4)	0.0630(17)
H54A	0.337537	0.468815	0.516531	0.095
H54B	0.274264	0.407541	0.560816	0.095
H54C	0.206972	0.443605	0.475569	0.095
C55	0.1890(4)	0.3509(2)	0.1138(4)	0.0467(12)
C56	0.2594(4)	0.6579(2)	0.8413(3)	0.0427(11)
C57	0.3429(4)	0.7021(2)	0.8740(3)	0.0425(11)
H57	0.430122	0.695440	0.869033	0.051
C58	0.2977(4)	0.7558(2)	0.9139(3)	0.0446(11)
H58	0.353891	0.786908	0.937183	0.054
C59	0.1696(4)	0.7650(2)	0.9207(3)	0.0480(11)
H59	0.138503	0.802667	0.947791	0.058
C60	0.0877(4)	0.7193(2)	0.8879(3)	0.0445(11)
H60	0.000264	0.725353	0.893143	0.053

C61	0.1328(4)	0.6655(2)	0.8477(3)	0.0448(11)
H61	0.077338	0.633900	0.824769	0.054
F5A	1.1418(13)	0.7435(7)	0.3422(11)	0.058(4)
C62A	1.0445(16)	0.7565(11)	0.2848(13)	0.038(3)
C63A	0.950(2)	0.7922(15)	0.3182(17)	0.037(3)
H63A	0.952616	0.807907	0.380459	0.044
C64A	0.851(3)	0.805(2)	0.259(2)	0.036(3)
H64A	0.783595	0.829663	0.280940	0.043
C65A	0.849(4)	0.782(3)	0.170(3)	0.034(3)
H65A	0.780601	0.791466	0.129209	0.041
C66A	0.946(4)	0.747(3)	0.138(3)	0.035(3)
H66A	0.944862	0.731091	0.076075	0.042
C67A	1.046(3)	0.733(3)	0.196(2)	0.037(3)
H67A	1.113287	0.708045	0.175507	0.044
F5B	0.9544(6)	0.8092(3)	0.4065(4)	0.0511(16)
C62B	0.9467(8)	0.7902(5)	0.3158(5)	0.038(2)
C63B	0.8420(12)	0.8058(9)	0.2646(9)	0.036(2)
H63B	0.776488	0.829485	0.291581	0.044
C64B	0.8348(17)	0.7861(12)	0.1725(13)	0.033(2)
H64B	0.763196	0.796012	0.135392	0.040
C65B	0.9313(19)	0.7521(13)	0.1341(14)	0.033(2)
H65B	0.925901	0.738757	0.070746	0.040
C66B	1.0355(17)	0.7373(12)	0.1879(12)	0.036(2)
H66B	1.101768	0.714023	0.161204	0.043
C67B	1.0441(12)	0.7563(9)	0.2803(8)	0.038(2)
H67B	1.115102	0.746212	0.317977	0.045
F5C	0.7625(8)	0.8304(4)	0.3058(6)	0.064(2)
C62C	0.8625(8)	0.7987(6)	0.2715(7)	0.035(2)
C63C	0.8582(14)	0.7796(12)	0.1796(10)	0.034(2)
H63C	0.787536	0.788364	0.140739	0.041
C64C	0.9602(19)	0.7473(15)	0.1454(14)	0.034(3)
H64C	0.959690	0.733256	0.082311	0.041
C65C	1.0629(19)	0.7352(14)	0.2029(15)	0.037(3)
H65C	1.132546	0.713111	0.178971	0.045
C66C	1.0639(16)	0.7553(12)	0.2951(12)	0.037(2)
H66C	1.134560	0.747097	0.334186	0.045
C67C	0.9624(12)	0.7874(9)	0.3307(9)	0.036(2)
H67C	0.962007	0.801225	0.393900	0.043

U_{eq} is defined as 1/3 of the trace of the orthogonalized U_{ij} tensor.

Table S8: Bond lengths and angles for compound **2**.

**Atom–Atom**	**Length [Å]**
Cu1–N4	1.844(3)
Cu1–N1	1.844(3)
S1–O3	1.432(3)
S1–O1	1.435(3)
S1–O2	1.437(3)
S1–C55	1.820(4)
F1–C55	1.321(6)
F2–C55	1.322(5)
F3–C55	1.318(6)
F4–C56	1.352(5)
N1–C1	1.304(4)

N1–H1	0.8800
N2–C1	1.363(4)
N2–C2	1.405(4)
N2–C4	1.438(4)
N3–C1	1.370(4)
N3–C3	1.395(5)
N3–C16	1.445(4)
N4–C28	1.315(4)
N4–H4	0.8800
N5–C28	1.360(4)
N5–C29	1.399(5)
N5–C31	1.433(4)
N6–C28	1.356(4)
N6–C30	1.404(4)
N6–C43	1.438(4)
C2–C3	1.338(5)
C2–H2	0.9500
C3–H3	0.9500
C4–C9	1.398(5)
C4–C5	1.403(5)
C5–C6	1.389(5)
C5–C10	1.521(5)
C6–C7	1.376(6)
C6–H6	0.9500
C7–C8	1.389(6)
C7–H7	0.9500
C8–C9	1.396(5)
C8–H8	0.9500
C9–C13	1.522(5)
C10–C11	1.521(6)
C10–C12	1.534(5)
C10–H10	1.0000
C11–H11A	0.9800
C11–H11B	0.9800
C11–H11C	0.9800
C12–H12A	0.9800
C12–H12B	0.9800
C12–H12C	0.9800
C13–C15	1.522(5)
C13–C14	1.525(5)
C13–H13	1.0000
C14–H14A	0.9800
C14–H14B	0.9800
C14–H14C	0.9800
C15–H15A	0.9800
C15–H15B	0.9800
C15–H15C	0.9800
C16–C17	1.395(5)
C16–C21	1.402(5)
C17–C18	1.398(5)
C17–C22	1.526(5)
C18–C19	1.379(6)
C18–H18	0.9500
C19–C20	1.386(5)
C19–H19	0.9500

C20–C21	1.387(5)
C20–H20	0.9500
C21–C25	1.523(5)
C22–C23	1.499(6)
C22–C24	1.502(6)
C22–H22	1.0000
C23–H23A	0.9800
C23–H23B	0.9800
C23–H23C	0.9800
C24–H24A	0.9800
C24–H24B	0.9800
C24–H24C	0.9800
C25–C26	1.526(6)
C25–C27	1.527(6)
C25–H25	1.0000
C26–H26A	0.9800
C26–H26B	0.9800
C26–H26C	0.9800
C27–H27A	0.9800
C27–H27B	0.9800
C27–H27C	0.9800
C29–C30	1.327(5)
C29–H29	0.9500
C30–H30	0.9500
C31–C36	1.396(5)
C31–C32	1.401(5)
C32–C33	1.397(6)
C32–C37	1.511(5)
C33–C34	1.380(6)
C33–H33	0.9500
C34–C35	1.370(6)
C34–H34	0.9500
C35–C36	1.388(6)
C35–H35	0.9500
C36–C40	1.524(5)
C37–C39	1.528(6)
C37–C38	1.528(6)
C37–H37	1.0000
C38–H38A	0.9800
C38–H38B	0.9800
C38–H38C	0.9800
C39–H39A	0.9800
C39–H39B	0.9800
C39–H39C	0.9800
C40–C41	1.528(5)
C40–C42	1.533(5)
C40–H40	1.0000
C41–H41A	0.9800
C41–H41B	0.9800
C41–H41C	0.9800
C42–H42A	0.9800
C42–H42B	0.9800
C42–H42C	0.9800
C43–C44	1.396(5)
C43–C48	1.397(5)

C44–C45	1.392(5)
C44–C49	1.520(5)
C45–C46	1.385(5)
C45–H45	0.9500
C46–C47	1.383(5)
C46–H46	0.9500
C47–C48	1.399(5)
C47–H47	0.9500
C48–C52	1.525(5)
C49–C50	1.518(6)
C49–C51	1.526(5)
C49–H49	1.0000
C50–H50A	0.9800
C50–H50B	0.9800
C50–H50C	0.9800
C51–H51A	0.9800
C51–H51B	0.9800
C51–H51C	0.9800
C52–C53	1.506(5)
C52–C54	1.527(6)
C52–H52	1.0000
C53–H53A	0.9800
C53–H53B	0.9800
C53–H53C	0.9800
C54–H54A	0.9800
C54–H54B	0.9800
C54–H54C	0.9800
C56–C57	1.372(7)
C56–C61	1.372(6)
C57–C58	1.365(7)
C57–H57	0.9500
C58–C59	1.392(6)
C58–H58	0.9500
C59–C60	1.384(7)
C59–H59	0.9500
C60–C61	1.369(7)
C60–H60	0.9500
C61–H61	0.9500
F5A–C62A	1.345(13)
C62A–C63A	1.357(13)
C62A–C67A	1.357(13)
C63A–C64A	1.369(13)
C63A–H63A	0.9500
C64A–C65A	1.368(13)
C64A–H64A	0.9500
C65A–C66A	1.368(13)
C65A–H65A	0.9500
C66A–C67A	1.369(13)
C66A–H66A	0.9500
C67A–H67A	0.9500
F5B–C62B	1.358(6)
C62B–C67B	1.371(6)
C62B–C63B	1.371(6)
C63B–C64B	1.383(6)
C63B–H63B	0.9500

C64B–C65B	1.382(6)
C64B–H64B	0.9500
C65B–C66B	1.382(6)
C65B–H65B	0.9500
C66B–C67B	1.383(6)
C66B–H66B	0.9500
C67B–H67B	0.9500
F5C–C62C	1.362(6)
C62C–C63C	1.375(6)
C62C–C67C	1.375(7)
C63C–C64C	1.387(7)
C63C–H63C	0.9500
C64C–C65C	1.386(7)
C64C–H64C	0.9500
C65C–C66C	1.386(7)
C65C–H65C	0.9500
C66C–C67C	1.387(7)
C66C–H66C	0.9500
C67C–H67C	0.9500
**Atom–Atom–Atom**	**Angle [°]**
N4–Cu1–N1	179.81(16)
O3–S1–O1	114.04(19)
O3–S1–O2	114.86(16)
O1–S1–O2	115.39(18)
O3–S1–C55	104.0(2)
O1–S1–C55	103.4(2)
O2–S1–C55	102.88(18)
C1–N1–Cu1	128.6(2)
C1–N1–H1	115.7
Cu1–N1–H1	115.7
C1–N2–C2	109.5(3)
C1–N2–C4	121.5(3)
C2–N2–C4	129.0(3)
C1–N3–C3	109.2(3)
C1–N3–C16	122.7(3)
C3–N3–C16	128.1(3)
C28–N4–Cu1	128.1(2)
C28–N4–H4	115.9
Cu1–N4–H4	115.9
C28–N5–C29	109.0(3)
C28–N5–C31	122.3(3)
C29–N5–C31	128.3(3)
C28–N6–C30	109.2(3)
C28–N6–C43	124.3(3)
C30–N6–C43	126.5(3)
N1–C1–N2	128.0(3)
N1–C1–N3	125.9(3)
N2–C1–N3	106.1(3)
C3–C2–N2	107.1(3)
C3–C2–H2	126.4
N2–C2–H2	126.4
C2–C3–N3	108.1(3)
C2–C3–H3	126.0
N3–C3–H3	126.0

C9–C4–C5	123.1(3)
C9–C4–N2	118.0(3)
C5–C4–N2	118.7(3)
C6–C5–C4	117.1(3)
C6–C5–C10	121.4(3)
C4–C5–C10	121.5(3)
C7–C6–C5	121.1(4)
C7–C6–H6	119.5
C5–C6–H6	119.5
C6–C7–C8	121.0(4)
C6–C7–H7	119.5
C8–C7–H7	119.5
C7–C8–C9	120.3(4)
C7–C8–H8	119.9
C9–C8–H8	119.9
C8–C9–C4	117.4(3)
C8–C9–C13	120.1(3)
C4–C9–C13	122.5(3)
C11–C10–C5	111.2(3)
C11–C10–C12	110.2(3)
C5–C10–C12	112.3(3)
C11–C10–H10	107.6
C5–C10–H10	107.6
C12–C10–H10	107.6
C10–C11–H11A	109.5
C10–C11–H11B	109.5
H11A–C11–H11B	109.5
C10–C11–H11C	109.5
H11A–C11–H11C	109.5
H11B–C11–H11C	109.5
C10–C12–H12A	109.5
C10–C12–H12B	109.5
H12A–C12–H12B	109.5
C10–C12–H12C	109.5
H12A–C12–H12C	109.5
H12B–C12–H12C	109.5
C15–C13–C9	110.9(3)
C15–C13–C14	111.1(4)
C9–C13–C14	111.3(3)
C15–C13–H13	107.8
C9–C13–H13	107.8
C14–C13–H13	107.8
C13–C14–H14A	109.5
C13–C14–H14B	109.5
H14A–C14–H14B	109.5
C13–C14–H14C	109.5
H14A–C14–H14C	109.5
H14B–C14–H14C	109.5
C13–C15–H15A	109.5
C13–C15–H15B	109.5
H15A–C15–H15B	109.5
C13–C15–H15C	109.5
H15A–C15–H15C	109.5
H15B–C15–H15C	109.5
C17–C16–C21	122.8(3)

C17–C16–N3	118.7(3)
C21–C16–N3	118.5(3)
C16–C17–C18	117.5(3)
C16–C17–C22	122.2(3)
C18–C17–C22	120.3(3)
C19–C18–C17	120.8(3)
C19–C18–H18	119.6
C17–C18–H18	119.6
C18–C19–C20	120.4(3)
C18–C19–H19	119.8
C20–C19–H19	119.8
C19–C20–C21	121.1(4)
C19–C20–H20	119.5
C21–C20–H20	119.5
C20–C21–C16	117.4(3)
C20–C21–C25	121.2(3)
C16–C21–C25	121.3(3)
C23–C22–C24	111.6(4)
C23–C22–C17	111.6(4)
C24–C22–C17	110.6(3)
C23–C22–H22	107.6
C24–C22–H22	107.6
C17–C22–H22	107.6
C22–C23–H23A	109.5
C22–C23–H23B	109.5
H23A–C23–H23B	109.5
C22–C23–H23C	109.5
H23A–C23–H23C	109.5
H23B–C23–H23C	109.5
C22–C24–H24A	109.5
C22–C24–H24B	109.5
H24A–C24–H24B	109.5
C22–C24–H24C	109.5
H24A–C24–H24C	109.5
H24B–C24–H24C	109.5
C21–C25–C26	112.9(3)
C21–C25–C27	110.2(3)
C26–C25–C27	110.7(3)
C21–C25–H25	107.6
C26–C25–H25	107.6
C27–C25–H25	107.6
C25–C26–H26A	109.5
C25–C26–H26B	109.5
H26A–C26–H26B	109.5
C25–C26–H26C	109.5
H26A–C26–H26C	109.5
H26B–C26–H26C	109.5
C25–C27–H27A	109.5
C25–C27–H27B	109.5
H27A–C27–H27B	109.5
C25–C27–H27C	109.5
H27A–C27–H27C	109.5
H27B–C27–H27C	109.5
N4–C28–N6	125.7(3)
N4–C28–N5	127.8(3)

N6–C28–N5	106.6(3)
C30–C29–N5	107.9(3)
C30–C29–H29	126.1
N5–C29–H29	126.1
C29–C30–N6	107.4(3)
C29–C30–H30	126.3
N6–C30–H30	126.3
C36–C31–C32	123.4(3)
C36–C31–N5	118.4(3)
C32–C31–N5	118.1(3)
C33–C32–C31	116.3(4)
C33–C32–C37	121.2(3)
C31–C32–C37	122.4(3)
C34–C33–C32	121.2(4)
C34–C33–H33	119.4
C32–C33–H33	119.4
C35–C34–C33	120.8(4)
C35–C34–H34	119.6
C33–C34–H34	119.6
C34–C35–C36	120.9(4)
C34–C35–H35	119.5
C36–C35–H35	119.5
C35–C36–C31	117.4(4)
C35–C36–C40	121.3(4)
C31–C36–C40	121.3(3)
C32–C37–C39	110.6(4)
C32–C37–C38	112.0(4)
C39–C37–C38	110.9(3)
C32–C37–H37	107.7
C39–C37–H37	107.7
C38–C37–H37	107.7
C37–C38–H38A	109.5
C37–C38–H38B	109.5
H38A–C38–H38B	109.5
C37–C38–H38C	109.5
H38A–C38–H38C	109.5
H38B–C38–H38C	109.5
C37–C39–H39A	109.5
C37–C39–H39B	109.5
H39A–C39–H39B	109.5
C37–C39–H39C	109.5
H39A–C39–H39C	109.5
H39B–C39–H39C	109.5
C36–C40–C41	112.2(3)
C36–C40–C42	110.5(3)
C41–C40–C42	111.3(3)
C36–C40–H40	107.5
C41–C40–H40	107.5
C42–C40–H40	107.5
C40–C41–H41A	109.5
C40–C41–H41B	109.5
H41A–C41–H41B	109.5
C40–C41–H41C	109.5
H41A–C41–H41C	109.5
H41B–C41–H41C	109.5

C40–C42–H42A	109.5
C40–C42–H42B	109.5
H42A–C42–H42B	109.5
C40–C42–H42C	109.5
H42A–C42–H42C	109.5
H42B–C42–H42C	109.5
C44–C43–C48	123.2(3)
C44–C43–N6	118.4(3)
C48–C43–N6	118.4(3)
C45–C44–C43	117.4(3)
C45–C44–C49	121.7(3)
C43–C44–C49	120.9(3)
C46–C45–C44	120.9(3)
C46–C45–H45	119.6
C44–C45–H45	119.6
C47–C46–C45	120.5(3)
C47–C46–H46	119.7
C45–C46–H46	119.7
C46–C47–C48	120.8(3)
C46–C47–H47	119.6
C48–C47–H47	119.6
C43–C48–C47	117.2(3)
C43–C48–C52	122.4(3)
C47–C48–C52	120.4(3)
C50–C49–C44	110.6(3)
C50–C49–C51	110.6(4)
C44–C49–C51	113.3(3)
C50–C49–H49	107.3
C44–C49–H49	107.3
C51–C49–H49	107.3
C49–C50–H50A	109.5
C49–C50–H50B	109.5
H50A–C50–H50B	109.5
C49–C50–H50C	109.5
H50A–C50–H50C	109.5
H50B–C50–H50C	109.5
C49–C51–H51A	109.5
C49–C51–H51B	109.5
H51A–C51–H51B	109.5
C49–C51–H51C	109.5
H51A–C51–H51C	109.5
H51B–C51–H51C	109.5
C53–C52–C48	111.6(3)
C53–C52–C54	110.5(4)
C48–C52–C54	110.8(3)
C53–C52–H52	107.9
C48–C52–H52	107.9
C54–C52–H52	107.9
C52–C53–H53A	109.5
C52–C53–H53B	109.5
H53A–C53–H53B	109.5
C52–C53–H53C	109.5
H53A–C53–H53C	109.5
H53B–C53–H53C	109.5
C52–C54–H54A	109.5

C52–C54–H54B	109.5
H54A–C54–H54B	109.5
C52–C54–H54C	109.5
H54A–C54–H54C	109.5
H54B–C54–H54C	109.5
F3–C55–F1	106.9(4)
F3–C55–F2	107.5(4)
F1–C55–F2	107.8(4)
F3–C55–S1	110.5(3)
F1–C55–S1	111.5(3)
F2–C55–S1	112.5(3)
F4–C56–C57	119.9(4)
F4–C56–C61	117.6(4)
C57–C56–C61	122.5(4)
C58–C57–C56	118.5(4)
C58–C57–H57	120.7
C56–C57–H57	120.7
C57–C58–C59	120.2(4)
C57–C58–H58	119.9
C59–C58–H58	119.9
C60–C59–C58	120.0(4)
C60–C59–H59	120.0
C58–C59–H59	120.0
C61–C60–C59	119.9(4)
C61–C60–H60	120.1
C59–C60–H60	120.1
C60–C61–C56	118.9(4)
C60–C61–H61	120.6
C56–C61–H61	120.6
F5A–C62A–C63A	118.3
F5A–C62A–C67A	118.3
C63A–C62A–C67A	123.4
C62A–C63A–C64A	117.9
C62A–C63A–H63A	121.1
C64A–C63A–H63A	121.1
C65A–C64A–C63A	120.4
C65A–C64A–H64A	119.8
C63A–C64A–H64A	119.8
C66A–C65A–C64A	120.1
C66A–C65A–H65A	120.0
C64A–C65A–H65A	120.0
C65A–C66A–C67A	120.4
C65A–C66A–H66A	119.8
C67A–C66A–H66A	119.8
C62A–C67A–C66A	117.9
C62A–C67A–H67A	121.1
C66A–C67A–H67A	121.1
F5B–C62B–C67B	118.3
F5B–C62B–C63B	118.3
C67B–C62B–C63B	123.4
C62B–C63B–C64B	117.9
C62B–C63B–H63B	121.1
C64B–C63B–H63B	121.1
C65B–C64B–C63B	120.4
C65B–C64B–H64B	119.8

C63B–C64B–H64B	119.8
C66B–C65B–C64B	120.1
C66B–C65B–H65B	120.0
C64B–C65B–H65B	120.0
C65B–C66B–C67B	120.4
C65B–C66B–H66B	119.8
C67B–C66B–H66B	119.8
C62B–C67B–C66B	117.9
C62B–C67B–H67B	121.1
C66B–C67B–H67B	121.1
F5C–C62C–C63C	118.3
F5C–C62C–C67C	118.3
C63C–C62C–C67C	123.4
C62C–C63C–C64C	117.9
C62C–C63C–H63C	121.1
C64C–C63C–H63C	121.1
C65C–C64C–C63C	120.4
C65C–C64C–H64C	119.8
C63C–C64C–H64C	119.8
C64C–C65C–C66C	120.1
C64C–C65C–H65C	120.0
C66C–C65C–H65C	120.0
C65C–C66C–C67C	120.4
C65C–C66C–H66C	119.8
C67C–C66C–H66C	119.8
C62C–C67C–C66C	117.9
C62C–C67C–H67C	121.1
C66C–C67C–H67C	121.1

Table S9: Torsion angles for compound **2**.

Atom–Atom–Atom–Atom	Torsion Angle [°]
Cu1–N1–C1–N2	173.8(3)
Cu1–N1–C1–N3	−6.0(5)
C2–N2–C1–N1	−179.1(3)
C4–N2–C1–N1	0.5(5)
C2–N2–C1–N3	0.7(4)
C4–N2–C1–N3	−179.7(3)
C3–N3–C1–N1	179.1(3)
C16–N3–C1–N1	−2.7(5)
C3–N3–C1–N2	−0.7(4)
C16–N3–C1–N2	177.5(3)
C1–N2–C2–C3	−0.4(4)
C4–N2–C2–C3	−180.0(3)
N2–C2–C3–N3	0.0(4)
C1–N3–C3–C2	0.5(4)
C16–N3–C3–C2	−177.6(3)
C1–N2–C4–C9	−85.6(4)
C2–N2–C4–C9	93.9(4)
C1–N2–C4–C5	91.3(4)
C2–N2–C4–C5	−89.2(4)
C9–C4–C5–C6	2.0(5)
N2–C4–C5–C6	−174.6(3)
C9–C4–C5–C10	−179.1(3)
N2–C4–C5–C10	4.3(5)
C4–C5–C6–C7	−0.8(6)
C10–C5–C6–C7	−179.7(4)
C5–C6–C7–C8	−1.0(6)
C6–C7–C8–C9	1.6(6)
C7–C8–C9–C4	−0.5(6)
C7–C8–C9–C13	178.0(4)
C5–C4–C9–C8	−1.4(5)
N2–C4–C9–C8	175.3(3)
C5–C4–C9–C13	−179.8(3)
N2–C4–C9–C13	−3.1(5)
C6–C5–C10–C11	81.1(4)
C4–C5–C10–C11	−97.8(4)
C6–C5–C10–C12	−42.9(5)
C4–C5–C10–C12	138.2(4)
C8–C9–C13–C15	61.5(5)
C4–C9–C13–C15	−120.1(4)
C8–C9–C13–C14	−62.6(5)
C4–C9–C13–C14	115.8(4)
C1–N3–C16–C17	95.5(4)
C3–N3–C16–C17	−86.7(4)
C1–N3–C16–C21	−82.3(4)
C3–N3–C16–C21	95.5(4)
C21–C16–C17–C18	0.4(5)
N3–C16–C17–C18	−177.3(3)
C21–C16–C17–C22	−179.8(3)
N3–C16–C17–C22	2.5(5)
C16–C17–C18–C19	0.2(5)
C22–C17–C18–C19	−179.5(3)
C17–C18–C19–C20	−1.2(6)
C18–C19–C20–C21	1.6(6)
C19–C20–C21–C16	−1.0(5)

C19–C20–C21–C25	−178.9(3)
C17–C16–C21–C20	0.0(5)
N3–C16–C21–C20	177.7(3)
C17–C16–C21–C25	177.9(3)
N3–C16–C21–C25	−4.4(5)
C16–C17–C22–C23	−119.9(5)
C18–C17–C22–C23	59.8(5)
C16–C17–C22–C24	115.1(4)
C18–C17–C22–C24	−65.1(5)
C20–C21–C25–C26	−36.3(5)
C16–C21–C25–C26	145.8(4)
C20–C21–C25–C27	88.1(4)
C16–C21–C25–C27	−89.7(4)
Cu1–N4–C28–N6	3.2(5)
Cu1–N4–C28–N5	−176.0(2)
C30–N6–C28–N4	−179.3(3)
C43–N6–C28–N4	1.9(5)
C30–N6–C28–N5	0.0(4)
C43–N6–C28–N5	−178.8(3)
C29–N5–C28–N4	178.8(3)
C31–N5–C28–N4	6.2(5)
C29–N5–C28–N6	−0.6(4)
C31–N5–C28–N6	−173.1(3)
C28–N5–C29–C30	0.9(4)
C31–N5–C29–C30	172.9(3)
N5–C29–C30–N6	−0.9(4)
C28–N6–C30–C29	0.5(4)
C43–N6–C30–C29	179.3(3)
C28–N5–C31–C36	82.8(4)
C29–N5–C31–C36	−88.3(4)
C28–N5–C31–C32	−94.8(4)
C29–N5–C31–C32	94.2(4)
C36–C31–C32–C33	0.0(5)
N5–C31–C32–C33	177.4(3)
C36–C31–C32–C37	−179.2(4)
N5–C31–C32–C37	−1.8(5)
C31–C32–C33–C34	−0.3(6)
C37–C32–C33–C34	179.0(4)
C32–C33–C34–C35	0.5(7)
C33–C34–C35–C36	−0.5(7)
C34–C35–C36–C31	0.2(6)
C34–C35–C36–C40	−179.1(4)
C32–C31–C36–C35	0.0(5)
N5–C31–C36–C35	−177.4(3)
C32–C31–C36–C40	179.3(3)
N5–C31–C36–C40	1.9(5)
C33–C32–C37–C39	−74.4(5)
C31–C32–C37–C39	104.8(4)
C33–C32–C37–C38	49.8(5)
C31–C32–C37–C38	−131.0(4)
C35–C36–C40–C41	−55.6(5)
C31–C36–C40–C41	125.1(4)
C35–C36–C40–C42	69.2(5)
C31–C36–C40–C42	−110.1(4)
C28–N6–C43–C44	−85.5(4)

C30–N6–C43–C44	95.9(4)
C28–N6–C43–C48	93.7(4)
C30–N6–C43–C48	−84.9(4)
C48–C43–C44–C45	−0.2(5)
N6–C43–C44–C45	178.9(3)
C48–C43–C44–C49	178.3(3)
N6–C43–C44–C49	−2.6(5)
C43–C44–C45–C46	0.1(5)
C49–C44–C45–C46	−178.3(3)
C44–C45–C46–C47	0.4(5)
C45–C46–C47–C48	−1.0(5)
C44–C43–C48–C47	−0.3(5)
N6–C43–C48–C47	−179.4(3)
C44–C43–C48–C52	179.0(3)
N6–C43–C48–C52	−0.1(5)
C46–C47–C48–C43	0.9(5)
C46–C47–C48–C52	−178.4(3)
C45–C44–C49–C50	91.7(4)
C43–C44–C49–C50	−86.8(4)
C45–C44–C49–C51	−33.2(5)
C43–C44–C49–C51	148.4(4)
C43–C48–C52–C53	113.3(4)
C47–C48–C52–C53	−67.5(5)
C43–C48–C52–C54	−123.2(4)
C47–C48–C52–C54	56.1(5)
O3–S1–C55–F3	−57.1(3)
O1–S1–C55–F3	−176.5(3)
O2–S1–C55–F3	63.0(4)
O3–S1–C55–F1	−175.7(3)
O1–S1–C55–F1	64.8(4)
O2–S1–C55–F1	−55.6(4)
O3–S1–C55–F2	63.0(4)
O1–S1–C55–F2	−56.4(4)
O2–S1–C55–F2	−176.8(4)
F4–C56–C57–C58	177.7(4)
C61–C56–C57–C58	−0.5(7)
C56–C57–C58–C59	−0.1(7)
C57–C58–C59–C60	0.8(7)
C58–C59–C60–C61	−0.8(7)
C59–C60–C61–C56	0.2(7)
F4–C56–C61–C60	−177.8(4)
C57–C56–C61–C60	0.4(7)
F5A–C62A–C63A–C64A	−179.8
C67A–C62A–C63A–C64A	0.2
C62A–C63A–C64A–C65A	−0.4
C63A–C64A–C65A–C66A	0.2
C64A–C65A–C66A–C67A	0.2
F5A–C62A–C67A–C66A	−179.8
C63A–C62A–C67A–C66A	0.2
C65A–C66A–C67A–C62A	−0.4
F5B–C62B–C63B–C64B	−179.8
C67B–C62B–C63B–C64B	0.2
C62B–C63B–C64B–C65B	−0.4
C63B–C64B–C65B–C66B	0.2
C64B–C65B–C66B–C67B	0.2

F5B–C62B–C67B–C66B	−179.8
C63B–C62B–C67B–C66B	0.2
C65B–C66B–C67B–C62B	−0.4
F5C–C62C–C63C–C64C	−179.8
C67C–C62C–C63C–C64C	0.2
C62C–C63C–C64C–C65C	−0.4
C63C–C64C–C65C–C66C	0.2
C64C–C65C–C66C–C67C	0.2
F5C–C62C–C67C–C66C	−179.8
C63C–C62C–C67C–C66C	0.2
C65C–C66C–C67C–C62C	−0.4

Table S10: Hydrogen bonds for compound **2**.

**D–H···A [Å]**	**d(D–H) [Å]**	**d(H···A) [Å]**	**d(D···A) [Å]**	**<(DHA) [°]**
C3–H3···O3[#1]	0.95	2.34	3.179(5)	147.8
C30–H30···O3	0.95	2.37	3.047(4)	128.1

Symmetry transformations used to generate equivalent atoms:
#1: 1-X, 0.5+Y, 1-Z;

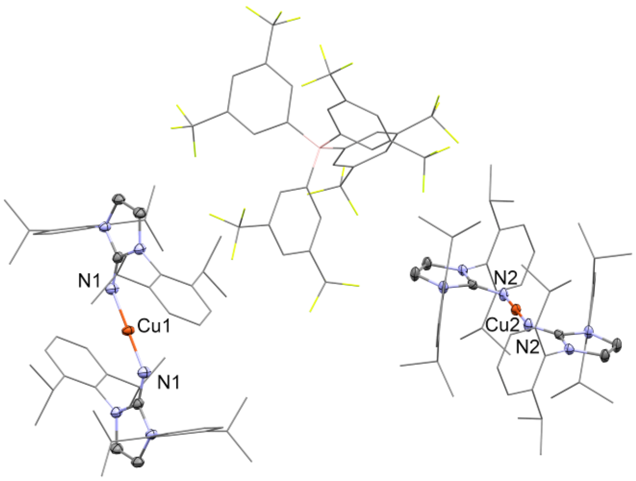

Table S11: Crystal data and structure refinement for [Cu(DippImH)$_2$][BAr^F] (**3**).

CCDC number	2363595
Empirical formula	$C_{86}H_{86}BCuF_{24}N_6$
Formula weight	1733.95
Temperature [K]	100(2)
Crystal system	triclinic
Space group (number)	$P\bar{1}$ (2)
a [Å]	12.8005(12)
b [Å]	13.6014(13)
c [Å]	25.404(2)
α [°]	93.948(3)
β [°]	97.557(3)
γ [°]	102.824(3)
Volume [Å^3]	4253.1(7)
Z	2
ρ_{calc} [gcm^{-3}]	1.354
μ [mm^{-1}]	0.357
F(000)	1788
Crystal size [mm^3]	0.139×0.223×0.228
Crystal colour	colourless
Crystal shape	block
Radiation	MoK_α (λ=0.71073 Å)
2θ range [°]	3.96 to 52.74 (0.80 Å)
Index ranges	−15 ≤ h ≤ 15<br>−16 ≤ k ≤ 16<br>−31 ≤ l ≤ 31
Reflections collected	269350
Independent reflections	17364<br>R_{int} = 0.0462<br>R_{sigma} = 0.0178
Completeness to θ = 25.242°	99.9 %
Data / Restraints / Parameters	17364 / 474 / 1218
Goodness-of-fit on F^2	1.033
Final *R* indexes [I≥2σ(I)]	R_1 = 0.0342<br>wR_2 = 0.0782
Final *R* indexes [all data]	R_1 = 0.0412<br>wR_2 = 0.0820
Largest peak/hole [eÅ^{-3}]	0.43/−0.47

Table S12: Atomic coordinates and Ueq [Å^2] for compound **3**.

**Atom**	***x***	***y***	***z***	U_{eq}
Cu1	1.000000	1.000000	0.500000	0.02107(6)
Cu2	0.500000	1.000000	1.000000	0.01662(6)
F1	1.12143(7)	0.65954(7)	0.74514(4)	0.0294(2)
F2	1.04851(8)	0.67146(8)	0.66548(4)	0.0333(2)
F3	1.12503(7)	0.80647(7)	0.71806(4)	0.0313(2)
F4	0.84808(10)	0.97264(7)	0.75962(5)	0.0463(3)
F5	0.76803(10)	0.90514(8)	0.82153(4)	0.0393(3)
F6	0.68519(9)	0.88296(8)	0.74164(5)	0.0452(3)
F7	0.35612(11)	0.70318(9)	0.71040(5)	0.0524(3)
F8	0.41988(10)	0.63386(11)	0.64815(4)	0.0509(3)
F9	0.26802(10)	0.56262(10)	0.66911(6)	0.0645(4)
F10	0.4074(4)	0.3656(6)	0.8767(3)	0.0689(16)
F11	0.2586(6)	0.3888(8)	0.8327(4)	0.0374(13)
F12	0.3653(4)	0.5069(4)	0.88989(16)	0.0596(12)
F10A	0.2585(6)	0.4058(8)	0.8416(4)	0.0554(18)
F11A	0.4010(4)	0.4593(5)	0.89588(13)	0.0791(16)
F12A	0.3775(3)	0.3201(3)	0.8492(2)	0.0532(10)
F13	0.71690(8)	0.51409(9)	0.97589(4)	0.0379(2)
F14	0.83642(9)	0.64116(7)	0.96062(4)	0.0375(2)
F15	0.88429(9)	0.52286(9)	1.00304(4)	0.0413(3)
F16	0.8893(3)	0.1962(2)	0.82673(17)	0.0442(7)
F17	0.9806(3)	0.2528(3)	0.90365(8)	0.0470(7)
F18	1.0352(3)	0.3110(4)	0.83253(17)	0.0444(8)
F16A	1.0247(12)	0.2912(12)	0.9000(4)	0.046(2)
F17A	1.0163(13)	0.3010(18)	0.8190(6)	0.039(2)
F18A	0.8966(13)	0.1903(9)	0.8445(6)	0.041(2)
F19	0.50189(10)	0.11736(8)	0.68527(6)	0.0534(3)
F20	0.66144(11)	0.09574(8)	0.70136(5)	0.0512(3)
F21	0.58137(13)	0.07660(8)	0.62090(5)	0.0633(4)
F22	0.7176(12)	0.5054(7)	0.5546(6)	0.0368(17)
F23	0.6711(7)	0.3525(4)	0.5182(2)	0.0480(14)
F24	0.8370(6)	0.4174(10)	0.5535(4)	0.0659(19)
F22A	0.7198(15)	0.3478(5)	0.5197(3)	0.069(3)
F23A	0.8538(6)	0.4494(8)	0.5674(5)	0.0521(18)
F24A	0.7068(13)	0.4960(9)	0.5461(7)	0.0356(19)
N1	0.93663(12)	0.87643(10)	0.46118(5)	0.0241(3)
H1	0.9087(16)	0.8767(15)	0.4308(8)	0.036
N2	0.89185(10)	0.69541(9)	0.44519(5)	0.0194(3)
N3	0.97673(11)	0.76384(9)	0.52419(5)	0.0216(3)
N4	0.38084(10)	0.95504(10)	0.94740(5)	0.0189(3)
H4	0.3190(16)	0.9482(14)	0.9544(7)	0.028
N5	0.47360(10)	0.92454(10)	0.87547(5)	0.0191(3)
N6	0.29693(9)	0.87917(9)	0.85914(5)	0.0164(2)
C1	0.93524(12)	0.78586(11)	0.47521(6)	0.0194(3)
C2	0.90597(13)	0.61654(12)	0.47594(6)	0.0240(3)
H2	0.882500	0.546067	0.464701	0.029
C3	0.95853(14)	0.65849(12)	0.52403(6)	0.0254(3)
H3	0.979781	0.623007	0.552985	0.031
C4	0.82354(12)	0.69140(11)	0.39478(6)	0.0200(3)

C5	0.71417(13)	0.69055(12)	0.39551(6)	0.0244(3)
C6	0.65248(14)	0.69902(13)	0.34727(7)	0.0307(4)
H6	0.577855	0.698337	0.346223	0.037
C7	0.69808(15)	0.70839(13)	0.30098(7)	0.0325(4)
H7	0.655125	0.716122	0.268758	0.039
C8	0.80566(14)	0.70663(13)	0.30113(6)	0.0279(4)
H8	0.835429	0.711696	0.268811	0.033
C9	0.87113(13)	0.69749(11)	0.34815(6)	0.0217(3)
C10	0.66293(14)	0.68248(14)	0.44610(7)	0.0311(4)
H10	0.720514	0.679027	0.476133	0.037
C11	0.57365(17)	0.58507(16)	0.44153(9)	0.0453(5)
H11A	0.541911	0.581159	0.474606	0.068
H11B	0.604660	0.526266	0.435545	0.068
H11C	0.517250	0.585459	0.411500	0.068
C12	0.61980(16)	0.77629(15)	0.45928(8)	0.0395(4)
H12A	0.588247	0.769712	0.492350	0.059
H12B	0.564124	0.782234	0.430049	0.059
H12C	0.679428	0.836932	0.463870	0.059
C13	0.98838(13)	0.69321(12)	0.34754(6)	0.0251(3)
H13	1.021024	0.687478	0.384864	0.030
C14	0.99527(17)	0.59990(16)	0.31275(10)	0.0511(6)
H14A	1.071433	0.597709	0.313401	0.077
H14B	0.962425	0.603069	0.275999	0.077
H14C	0.956381	0.538821	0.326430	0.077
C15	1.05425(18)	0.78829(17)	0.32899(12)	0.0594(7)
H15A	1.129019	0.782000	0.328856	0.089
H15B	1.053860	0.847323	0.353296	0.089
H15C	1.022357	0.796851	0.292847	0.089
C16	1.02211(14)	0.84004(11)	0.56874(6)	0.0232(3)
C17	1.13466(14)	0.87515(12)	0.58017(6)	0.0251(3)
C18	1.17573(15)	0.95331(12)	0.62151(6)	0.0291(4)
H18	1.251802	0.979665	0.630005	0.035
C19	1.10711(16)	0.99249(12)	0.65004(6)	0.0311(4)
H19	1.136335	1.045737	0.677828	0.037
C20	0.99584(16)	0.95480(12)	0.63851(6)	0.0297(4)
H20	0.949973	0.981633	0.659156	0.036
C21	0.94998(14)	0.87840(12)	0.59727(6)	0.0256(3)
C22	1.21163(14)	0.83178(13)	0.55004(6)	0.0292(4)
H22A	1.167695	0.781375	0.520393	0.033(7)
H22B	1.173084	0.761383	0.534742	0.03(2)
C23	1.2764(4)	0.7765(4)	0.58783(12)	0.0494(10)
H23A	1.326657	0.748631	0.568651	0.074
H23B	1.226571	0.721262	0.600971	0.074
H23C	1.317399	0.824190	0.618058	0.074
C24	1.2873(4)	0.9136(2)	0.52591(17)	0.0498(10)
H24A	1.333153	0.882242	0.505330	0.075
H24B	1.333073	0.962249	0.554550	0.075
H24C	1.244328	0.948778	0.502338	0.075
C23A	1.3174(13)	0.8255(16)	0.5827(6)	0.051(3)
H23D	1.354599	0.784960	0.561594	0.076
H23E	1.302629	0.793686	0.615185	0.076
H23F	1.363499	0.893939	0.592302	0.076

C24A	1.2358(14)	0.8937(10)	0.5035(6)	0.038(3)
H24D	1.288675	0.868583	0.485006	0.057
H24E	1.265407	0.965096	0.516954	0.057
H24F	1.168740	0.887262	0.478586	0.057
C25	0.82796(15)	0.83968(13)	0.58289(6)	0.0295(4)
H25	0.811544	0.825915	0.543134	0.035
C26	0.78683(16)	0.73950(14)	0.60591(7)	0.0347(4)
H26A	0.709475	0.713421	0.592560	0.052
H26B	0.797620	0.750913	0.644959	0.052
H26C	0.827072	0.690091	0.594935	0.052
C27	0.76496(17)	0.91730(16)	0.59934(8)	0.0418(5)
H27A	0.688217	0.892668	0.584327	0.063
H27B	0.793979	0.982037	0.585850	0.063
H27C	0.772363	0.926877	0.638353	0.063
C28	0.38225(11)	0.92198(10)	0.89802(5)	0.0159(3)
C29	0.44428(13)	0.88201(12)	0.82228(6)	0.0240(3)
H29	0.492578	0.874086	0.797785	0.029
C30	0.33629(12)	0.85465(12)	0.81226(6)	0.0218(3)
H30	0.293780	0.824060	0.779281	0.026
C31	0.58245(11)	0.95951(12)	0.90384(6)	0.0192(3)
C32	0.63233(12)	1.06285(12)	0.90672(6)	0.0216(3)
C33	0.73912(13)	1.09360(13)	0.93248(6)	0.0257(3)
H33	0.775956	1.162951	0.934974	0.031
C34	0.79211(13)	1.02430(13)	0.95446(6)	0.0278(4)
H34	0.865488	1.046323	0.970999	0.033
C35	0.73939(13)	0.92329(13)	0.95260(6)	0.0260(3)
H35	0.776573	0.877005	0.968453	0.031
C36	0.63205(12)	0.88865(12)	0.92766(6)	0.0215(3)
C37	0.57155(13)	1.13788(13)	0.88343(7)	0.0282(4)
H37	0.492942	1.111088	0.885330	0.034
C38	0.58298(18)	1.14561(17)	0.82449(8)	0.0466(5)
H38A	0.540349	1.191775	0.809901	0.070
H38B	0.556510	1.078357	0.804606	0.070
H38C	0.659401	1.171640	0.821252	0.070
C39	0.60636(15)	1.24261(14)	0.91468(8)	0.0377(4)
H39A	0.556320	1.284061	0.902188	0.057
H39B	0.679888	1.275245	0.909161	0.057
H39C	0.605231	1.235795	0.952769	0.057
C40	0.57280(13)	0.77854(12)	0.92658(7)	0.0278(4)
H40	0.493480	0.774829	0.917623	0.033
C41	0.60425(16)	0.71320(15)	0.88252(9)	0.0415(5)
H41A	0.566541	0.642220	0.882835	0.062
H41B	0.682730	0.719296	0.888738	0.062
H41C	0.583623	0.736274	0.847785	0.062
C42	0.59100(17)	0.73691(15)	0.98049(8)	0.0408(4)
H42A	0.544515	0.668674	0.978886	0.061
H42B	0.573060	0.781378	1.008218	0.061
H42C	0.667108	0.733961	0.988949	0.061
C43	0.18568(11)	0.86484(11)	0.86757(5)	0.0170(3)
C44	0.13083(12)	0.76864(11)	0.87824(6)	0.0197(3)
C45	0.02456(13)	0.75808(13)	0.88805(6)	0.0263(3)
H45	−0.015477	0.693811	0.894893	0.032

C46	−0.02361(13)	0.83952(14)	0.88799(7)	0.0298(4)
H46	−0.095843	0.830777	0.895274	0.036
C47	0.03207(13)	0.93327(13)	0.87748(6)	0.0269(3)
H47	−0.002297	0.988411	0.877612	0.032
C48	0.13856(12)	0.94839(12)	0.86662(6)	0.0208(3)
C49	0.18657(13)	0.68133(12)	0.88212(7)	0.0249(3)
H49	0.241094	0.689493	0.856975	0.030
C50	0.10848(15)	0.57770(13)	0.86684(8)	0.0345(4)
H50A	0.149832	0.525265	0.864983	0.052
H50B	0.065234	0.577332	0.831965	0.052
H50C	0.060292	0.564007	0.893757	0.052
C51	0.24750(15)	0.68634(14)	0.93885(8)	0.0363(4)
H51A	0.284775	0.630784	0.940995	0.054
H51B	0.195837	0.680122	0.964369	0.054
H51C	0.300798	0.751308	0.947391	0.054
C52	0.19823(14)	1.05149(12)	0.85422(7)	0.0277(4)
H52A	0.273366	1.047303	0.849211	0.040(8)
H52B	0.277180	1.055596	0.865074	0.014(19)
C53	0.1421(5)	1.0809(4)	0.80139(15)	0.0411(11)
H53A	0.182967	1.146838	0.793544	0.062
H53B	0.068004	1.085107	0.805290	0.062
H53C	0.140017	1.029431	0.772087	0.062
C54	0.2068(6)	1.1334(4)	0.8996(2)	0.0433(12)
H54A	0.248013	1.198288	0.890701	0.065
H54B	0.243950	1.115158	0.932477	0.065
H54C	0.133981	1.139570	0.904959	0.065
C53A	0.1823(18)	1.0563(12)	0.7965(5)	0.041(3)
H53D	0.217801	1.124027	0.788458	0.061
H53E	0.104518	1.042654	0.783092	0.061
H53F	0.213728	1.005578	0.779176	0.061
C54A	0.1711(19)	1.1361(16)	0.8882(9)	0.043(4)
H54D	0.220756	1.200643	0.884723	0.065
H54E	0.178800	1.122018	0.925700	0.065
H54F	0.096399	1.140171	0.876255	0.065
C55	0.78470(12)	0.60957(11)	0.75368(5)	0.0170(3)
C56	0.88851(12)	0.61867(11)	0.73987(5)	0.0175(3)
H56	0.913544	0.558891	0.733329	0.021
C57	0.95636(12)	0.71193(11)	0.73537(6)	0.0184(3)
C58	0.92459(12)	0.80173(11)	0.74582(6)	0.0200(3)
H58	0.971187	0.865549	0.743169	0.024
C59	0.82276(13)	0.79512(11)	0.76023(6)	0.0198(3)
C60	0.75471(12)	0.70114(11)	0.76404(6)	0.0186(3)
H60	0.685380	0.699196	0.774040	0.022
C61	1.06219(12)	0.71309(12)	0.71638(6)	0.0219(3)
C62	0.78206(14)	0.88865(12)	0.77078(6)	0.0254(3)
C63	0.58501(12)	0.50344(10)	0.76492(6)	0.0174(3)
C64	0.52844(12)	0.55090(11)	0.72704(6)	0.0196(3)
H64	0.563333	0.577365	0.698544	0.024
C65	0.42362(12)	0.56054(11)	0.72972(6)	0.0219(3)
C66	0.36870(13)	0.52074(12)	0.76998(7)	0.0252(3)
H66	0.297374	0.528148	0.772419	0.030
C67	0.42112(13)	0.46999(12)	0.80640(6)	0.0244(3)

C68	0.52715(12)	0.46179(11)	0.80401(6)	0.0196(3)
H68	0.560880	0.426780	0.829810	0.024
C69	0.36820(13)	0.61464(13)	0.68948(7)	0.0279(4)
C70	0.36402(15)	0.42372(17)	0.84943(8)	0.0426(5)
C71	0.77009(11)	0.45979(11)	0.81304(6)	0.0171(3)
C72	0.77031(11)	0.50961(11)	0.86333(6)	0.0177(3)
H72	0.732433	0.562088	0.865417	0.021
C73	0.82361(12)	0.48542(11)	0.91018(6)	0.0192(3)
C74	0.88151(12)	0.41012(11)	0.90894(6)	0.0211(3)
H74	0.917819	0.392832	0.940807	0.025
C75	0.88441(12)	0.36128(11)	0.85967(6)	0.0211(3)
C76	0.82983(12)	0.38548(11)	0.81278(6)	0.0195(3)
H76	0.833478	0.350289	0.779693	0.023
C77	0.81585(13)	0.54023(12)	0.96214(6)	0.0245(3)
C78	0.94846(13)	0.28190(13)	0.85591(7)	0.0287(4)
C79	0.69880(11)	0.41792(11)	0.70624(6)	0.0177(3)
C80	0.65583(12)	0.31384(11)	0.70720(6)	0.0199(3)
H80	0.635594	0.290072	0.739551	0.024
C81	0.64167(13)	0.24402(11)	0.66258(6)	0.0223(3)
C82	0.66925(13)	0.27564(12)	0.61448(6)	0.0253(3)
H82	0.661099	0.228256	0.584096	0.030
C83	0.70903(13)	0.37826(12)	0.61199(6)	0.0239(3)
C84	0.72343(12)	0.44784(11)	0.65672(6)	0.0203(3)
H84	0.750787	0.517726	0.653573	0.024
C85	0.59695(15)	0.13389(12)	0.66718(7)	0.0309(4)
C86	0.73917(17)	0.41515(13)	0.56070(7)	0.0356(4)
B1	0.70961(13)	0.49708(12)	0.75946(6)	0.0162(3)

U_{eq} is defined as 1/3 of the trace of the orthogonalized U_{ij} tensor.

Table S13: Bond lengths and angles for compound **3**.

**Atom–Atom**	**Length [Å]**
Cu1–N1	1.8470(13)
Cu1–N1[#1]	1.8470(13)
Cu2–N4[#2]	1.8502(13)
Cu2–N4	1.8503(13)
F1–C61	1.3461(18)
F2–C61	1.3486(18)
F3–C61	1.3386(18)
F4–C62	1.3337(19)
F5–C62	1.3356(19)
F6–C62	1.342(2)
F7–C69	1.3314(19)
F8–C69	1.324(2)
F9–C69	1.336(2)
F10–C70	1.258(4)
F11–C70	1.328(7)
F12–C70	1.471(5)
F10A–C70	1.304(7)
F11A–C70	1.234(4)
F12A–C70	1.457(4)
F13–C77	1.3364(19)
F14–C77	1.3435(19)
F15–C77	1.3375(18)
F16–C78	1.350(3)
F17–C78	1.343(2)
F18–C78	1.324(3)
F16A–C78	1.363(8)
F17A–C78	1.360(12)
F18A–C78	1.268(11)
F19–C85	1.335(2)
F20–C85	1.330(2)
F21–C85	1.331(2)
F22–C86	1.331(7)
F23–C86	1.396(5)
F24–C86	1.283(6)
F22A–C86	1.299(6)
F23A–C86	1.421(8)
F24A–C86	1.319(9)
N1–C1	1.303(2)
N1–H1	0.81(2)
N2–C1	1.3638(19)
N2–C2	1.3992(19)

N2–C4	1.4430(19)
N3–C1	1.3668(19)
N3–C3	1.399(2)
N3–C16	1.4458(19)
N4–C28	1.3069(19)
N4–H4	0.82(2)
N5–C28	1.3628(18)
N5–C29	1.4015(19)
N5–C31	1.4428(18)
N6–C28	1.3647(18)
N6–C30	1.4004(18)
N6–C43	1.4403(18)
C2–C3	1.337(2)
C2–H2	0.9500
C3–H3	0.9500
C4–C5	1.400(2)
C4–C9	1.401(2)
C5–C6	1.394(2)
C5–C10	1.518(2)
C6–C7	1.381(3)
C6–H6	0.9500
C7–C8	1.382(2)
C7–H7	0.9500
C8–C9	1.396(2)
C8–H8	0.9500
C9–C13	1.517(2)
C10–C11	1.532(3)
C10–C12	1.532(2)
C10–H10	1.0000
C11–H11A	0.9800
C11–H11B	0.9800
C11–H11C	0.9800
C12–H12A	0.9800
C12–H12B	0.9800
C12–H12C	0.9800
C13–C15	1.520(3)
C13–C14	1.522(2)
C13–H13	1.0000
C14–H14A	0.9800
C14–H14B	0.9800
C14–H14C	0.9800
C15–H15A	0.9800
C15–H15B	0.9800
C15–H15C	0.9800
C16–C17	1.398(2)
C16–C21	1.410(2)
C17–C18	1.401(2)
C17–C22	1.516(2)
C18–C19	1.379(3)
C18–H18	0.9500
C19–C20	1.387(3)
C19–H19	0.9500
C20–C21	1.393(2)
C20–H20	0.9500
C21–C25	1.519(2)

C22–C23A	1.514(13)
C22–C24A	1.523(11)
C22–C24	1.524(3)
C22–C23	1.530(3)
C22–H22A	1.0000
C22–H22B	1.0000
C23–H23A	0.9800
C23–H23B	0.9800
C23–H23C	0.9800
C24–H24A	0.9800
C24–H24B	0.9800
C24–H24C	0.9800
C23A–H23D	0.9800
C23A–H23E	0.9800
C23A–H23F	0.9800
C24A–H24D	0.9800
C24A–H24E	0.9800
C24A–H24F	0.9800
C25–C27	1.532(2)
C25–C26	1.532(2)
C25–H25	1.0000
C26–H26A	0.9800
C26–H26B	0.9800
C26–H26C	0.9800
C27–H27A	0.9800
C27–H27B	0.9800
C27–H27C	0.9800
C29–C30	1.334(2)
C29–H29	0.9500
C30–H30	0.9500
C31–C36	1.396(2)
C31–C32	1.401(2)
C32–C33	1.395(2)
C32–C37	1.520(2)
C33–C34	1.385(2)
C33–H33	0.9500
C34–C35	1.384(2)
C34–H34	0.9500
C35–C36	1.398(2)
C35–H35	0.9500
C36–C40	1.518(2)
C37–C39	1.526(3)
C37–C38	1.532(2)
C37–H37	1.0000
C38–H38A	0.9800
C38–H38B	0.9800
C38–H38C	0.9800
C39–H39A	0.9800
C39–H39B	0.9800
C39–H39C	0.9800
C40–C42	1.527(3)
C40–C41	1.531(2)
C40–H40	1.0000
C41–H41A	0.9800
C41–H41B	0.9800

C41–H41C	0.9800
C42–H42A	0.9800
C42–H42B	0.9800
C42–H42C	0.9800
C43–C48	1.400(2)
C43–C44	1.403(2)
C44–C45	1.393(2)
C44–C49	1.517(2)
C45–C46	1.382(2)
C45–H45	0.9500
C46–C47	1.378(2)
C46–H46	0.9500
C47–C48	1.400(2)
C47–H47	0.9500
C48–C52	1.516(2)
C49–C50	1.528(2)
C49–C51	1.535(2)
C49–H49	1.0000
C50–H50A	0.9800
C50–H50B	0.9800
C50–H50C	0.9800
C51–H51A	0.9800
C51–H51B	0.9800
C51–H51C	0.9800
C52–C53A	1.462(12)
C52–C54A	1.520(17)
C52–C54	1.523(5)
C52–C53	1.558(4)
C52–H52A	1.0000
C52–H52B	1.0000
C53–H53A	0.9800
C53–H53B	0.9800
C53–H53C	0.9800
C54–H54A	0.9800
C54–H54B	0.9800
C54–H54C	0.9800
C53A–H53D	0.9800
C53A–H53E	0.9800
C53A–H53F	0.9800
C54A–H54D	0.9800
C54A–H54E	0.9800
C54A–H54F	0.9800
C55–C56	1.400(2)
C55–C60	1.401(2)
C55–B1	1.644(2)
C56–C57	1.390(2)
C56–H56	0.9500
C57–C58	1.390(2)
C57–C61	1.494(2)
C58–C59	1.385(2)
C58–H58	0.9500
C59–C60	1.395(2)
C59–C62	1.498(2)
C60–H60	0.9500
C63–C68	1.393(2)
C63–C64	1.406(2)
C63–B1	1.640(2)
C64–C65	1.387(2)
C64–H64	0.9500
C65–C66	1.389(2)
C65–C69	1.495(2)
C66–C67	1.383(2)
C66–H66	0.9500
C67–C68	1.395(2)
C67–C70	1.492(2)
C68–H68	0.9500
C71–C76	1.396(2)
C71–C72	1.404(2)
C71–B1	1.644(2)
C72–C73	1.389(2)
C72–H72	0.9500
C73–C74	1.392(2)
C73–C77	1.497(2)
C74–C75	1.385(2)
C74–H74	0.9500
C75–C76	1.397(2)
C75–C78	1.499(2)
C76–H76	0.9500
C79–C84	1.402(2)
C79–C80	1.403(2)
C79–B1	1.640(2)
C80–C81	1.393(2)
C80–H80	0.9500
C81–C82	1.386(2)
C81–C85	1.499(2)
C82–C83	1.384(2)
C82–H82	0.9500
C83–C84	1.393(2)
C83–C86	1.495(2)
C84–H84	0.9500

**Atom–Atom–Atom**	**Angle [°]**
N1–Cu1–N1$^{\#1}$	180.0
N4$^{\#2}$–Cu2–N4	180.0
C1–N1–Cu1	128.50(11)
C1–N1–H1	113.7(15)
Cu1–N1–H1	117.7(15)
C1–N2–C2	109.14(12)
C1–N2–C4	120.25(12)
C2–N2–C4	129.25(13)
C1–N3–C3	108.74(12)
C1–N3–C16	123.23(12)
C3–N3–C16	127.80(12)
C28–N4–Cu2	126.29(11)
C28–N4–H4	112.3(13)
Cu2–N4–H4	121.1(13)
C28–N5–C29	109.20(12)
C28–N5–C31	124.37(12)
C29–N5–C31	126.31(12)
C28–N6–C30	109.20(12)

C28–N6–C43	122.89(12)
C30–N6–C43	127.91(12)
N1–C1–N2	127.70(13)
N1–C1–N3	125.66(14)
N2–C1–N3	106.64(12)
C3–C2–N2	107.50(13)
C3–C2–H2	126.3
N2–C2–H2	126.2
C2–C3–N3	107.97(13)
C2–C3–H3	126.0
N3–C3–H3	126.0
C5–C4–C9	123.04(14)
C5–C4–N2	117.95(14)
C9–C4–N2	118.76(13)
C6–C5–C4	117.14(15)
C6–C5–C10	120.18(15)
C4–C5–C10	122.68(14)
C7–C6–C5	121.08(16)
C7–C6–H6	119.5
C5–C6–H6	119.5
C6–C7–C8	120.57(15)
C6–C7–H7	119.7
C8–C7–H7	119.7
C7–C8–C9	120.86(16)
C7–C8–H8	119.6
C9–C8–H8	119.6
C8–C9–C4	117.26(15)
C8–C9–C13	120.34(14)
C4–C9–C13	122.40(14)
C5–C10–C11	110.77(15)
C5–C10–C12	111.09(15)
C11–C10–C12	111.35(16)
C5–C10–H10	107.8
C11–C10–H10	107.8
C12–C10–H10	107.8
C10–C11–H11A	109.5
C10–C11–H11B	109.5
H11A–C11–H11B	109.5
C10–C11–H11C	109.5
H11A–C11–H11C	109.5
H11B–C11–H11C	109.5
C10–C12–H12A	109.5
C10–C12–H12B	109.5
H12A–C12–H12B	109.5
C10–C12–H12C	109.5
H12A–C12–H12C	109.5
H12B–C12–H12C	109.5
C9–C13–C15	112.22(15)
C9–C13–C14	110.64(14)
C15–C13–C14	110.16(17)
C9–C13–H13	107.9
C15–C13–H13	107.9
C14–C13–H13	107.9
C13–C14–H14A	109.5
C13–C14–H14B	109.5

H14A–C14–H14B	109.5
C13–C14–H14C	109.5
H14A–C14–H14C	109.5
H14B–C14–H14C	109.5
C13–C15–H15A	109.5
C13–C15–H15B	109.5
H15A–C15–H15B	109.5
C13–C15–H15C	109.5
H15A–C15–H15C	109.5
H15B–C15–H15C	109.5
C17–C16–C21	123.09(14)
C17–C16–N3	118.78(14)
C21–C16–N3	118.08(15)
C16–C17–C18	117.35(16)
C16–C17–C22	122.68(14)
C18–C17–C22	119.97(16)
C19–C18–C17	120.84(17)
C19–C18–H18	119.6
C17–C18–H18	119.6
C18–C19–C20	120.55(15)
C18–C19–H19	119.7
C20–C19–H19	119.7
C19–C20–C21	121.31(16)
C19–C20–H20	119.3
C21–C20–H20	119.3
C20–C21–C16	116.83(16)
C20–C21–C25	121.65(15)
C16–C21–C25	121.50(14)
C23A–C22–C17	115.9(6)
C23A–C22–C24A	109.2(7)
C17–C22–C24A	108.9(4)
C17–C22–C24	111.89(18)
C17–C22–C23	109.05(18)
C24–C22–C23	110.7(2)
C17–C22–H22A	108.4
C24–C22–H22A	108.4
C23–C22–H22A	108.4
C23A–C22–H22B	107.5
C17–C22–H22B	107.5
C24A–C22–H22B	107.5
C22–C23–H23A	109.5
C22–C23–H23B	109.5
H23A–C23–H23B	109.5
C22–C23–H23C	109.5
H23A–C23–H23C	109.5
H23B–C23–H23C	109.5
C22–C24–H24A	109.5
C22–C24–H24B	109.5
H24A–C24–H24B	109.5
C22–C24–H24C	109.5
H24A–C24–H24C	109.5
H24B–C24–H24C	109.5
C22–C23A–H23D	109.5
C22–C23A–H23E	109.5
H23D–C23A–H23E	109.5

C22–C23A–H23F	109.5
H23D–C23A–H23F	109.5
H23E–C23A–H23F	109.5
C22–C24A–H24D	109.5
C22–C24A–H24E	109.5
H24D–C24A–H24E	109.5
C22–C24A–H24F	109.5
H24D–C24A–H24F	109.5
H24E–C24A–H24F	109.5
C21–C25–C27	113.06(15)
C21–C25–C26	112.06(14)
C27–C25–C26	110.00(16)
C21–C25–H25	107.1
C27–C25–H25	107.1
C26–C25–H25	107.1
C25–C26–H26A	109.5
C25–C26–H26B	109.5
H26A–C26–H26B	109.5
C25–C26–H26C	109.5
H26A–C26–H26C	109.5
H26B–C26–H26C	109.5
C25–C27–H27A	109.5
C25–C27–H27B	109.5
H27A–C27–H27B	109.5
C25–C27–H27C	109.5
H27A–C27–H27C	109.5
H27B–C27–H27C	109.5
N4–C28–N5	124.94(13)
N4–C28–N6	128.72(13)
N5–C28–N6	106.34(12)
C30–C29–N5	107.64(13)
C30–C29–H29	126.2
N5–C29–H29	126.2
C29–C30–N6	107.63(13)
C29–C30–H30	126.2
N6–C30–H30	126.2
C36–C31–C32	123.41(14)
C36–C31–N5	118.15(13)
C32–C31–N5	118.43(14)
C33–C32–C31	117.06(15)
C33–C32–C37	121.78(14)
C31–C32–C37	121.14(14)
C34–C33–C32	120.81(15)
C34–C33–H33	119.6
C32–C33–H33	119.6
C35–C34–C33	120.76(15)
C35–C34–H34	119.6
C33–C34–H34	119.6
C34–C35–C36	120.73(15)
C34–C35–H35	119.6
C36–C35–H35	119.6
C31–C36–C35	117.11(14)
C31–C36–C40	121.82(14)
C35–C36–C40	121.07(15)
C32–C37–C39	112.85(14)
C32–C37–C38	110.93(15)
C39–C37–C38	110.45(15)
C32–C37–H37	107.5
C39–C37–H37	107.5
C38–C37–H37	107.5
C37–C38–H38A	109.5
C37–C38–H38B	109.5
H38A–C38–H38B	109.5
C37–C38–H38C	109.5
H38A–C38–H38C	109.5
H38B–C38–H38C	109.5
C37–C39–H39A	109.5
C37–C39–H39B	109.5
H39A–C39–H39B	109.5
C37–C39–H39C	109.5
H39A–C39–H39C	109.5
H39B–C39–H39C	109.5
C36–C40–C42	112.67(15)
C36–C40–C41	110.48(14)
C42–C40–C41	110.98(15)
C36–C40–H40	107.5
C42–C40–H40	107.5
C41–C40–H40	107.5
C40–C41–H41A	109.5
C40–C41–H41B	109.5
H41A–C41–H41B	109.5
C40–C41–H41C	109.5
H41A–C41–H41C	109.5
H41B–C41–H41C	109.5
C40–C42–H42A	109.5
C40–C42–H42B	109.5
H42A–C42–H42B	109.5
C40–C42–H42C	109.5
H42A–C42–H42C	109.5
H42B–C42–H42C	109.5
C48–C43–C44	123.07(14)
C48–C43–N6	118.45(13)
C44–C43–N6	118.43(13)
C45–C44–C43	117.07(14)
C45–C44–C49	121.22(14)
C43–C44–C49	121.62(13)
C46–C45–C44	121.12(15)
C46–C45–H45	119.4
C44–C45–H45	119.4
C47–C46–C45	120.67(15)
C47–C46–H46	119.7
C45–C46–H46	119.7
C46–C47–C48	120.92(15)
C46–C47–H47	119.5
C48–C47–H47	119.5
C47–C48–C43	117.14(14)
C47–C48–C52	120.37(14)
C43–C48–C52	122.49(14)
C44–C49–C50	113.10(14)
C44–C49–C51	109.68(14)

C50–C49–C51	110.07(14)
C44–C49–H49	107.9
C50–C49–H49	107.9
C51–C49–H49	107.9
C49–C50–H50A	109.5
C49–C50–H50B	109.5
H50A–C50–H50B	109.5
C49–C50–H50C	109.5
H50A–C50–H50C	109.5
H50B–C50–H50C	109.5
C49–C51–H51A	109.5
C49–C51–H51B	109.5
H51A–C51–H51B	109.5
C49–C51–H51C	109.5
H51A–C51–H51C	109.5
H51B–C51–H51C	109.5
C53A–C52–C48	109.3(5)
C53A–C52–C54A	116.6(8)
C48–C52–C54A	111.1(9)
C48–C52–C54	111.9(3)
C48–C52–C53	110.71(18)
C54–C52–C53	110.0(2)
C48–C52–H52A	108.0
C54–C52–H52A	108.0
C53–C52–H52A	108.0
C53A–C52–H52B	106.4
C48–C52–H52B	106.4
C54A–C52–H52B	106.4
C52–C53–H53A	109.5
C52–C53–H53B	109.5
H53A–C53–H53B	109.5
C52–C53–H53C	109.5
H53A–C53–H53C	109.5
H53B–C53–H53C	109.5
C52–C54–H54A	109.5
C52–C54–H54B	109.5
H54A–C54–H54B	109.5
C52–C54–H54C	109.5
H54A–C54–H54C	109.5
H54B–C54–H54C	109.5
C52–C53A–H53D	109.5
C52–C53A–H53E	109.5
H53D–C53A–H53E	109.5
C52–C53A–H53F	109.5
H53D–C53A–H53F	109.5
H53E–C53A–H53F	109.5
C52–C54A–H54D	109.5
C52–C54A–H54E	109.5
H54D–C54A–H54E	109.5
C52–C54A–H54F	109.5
H54D–C54A–H54F	109.5
H54E–C54A–H54F	109.5
C56–C55–C60	115.39(13)
C56–C55–B1	119.95(12)
C60–C55–B1	124.59(13)

C57–C56–C55	122.47(14)
C57–C56–H56	118.8
C55–C56–H56	118.8
C58–C57–C56	120.98(14)
C58–C57–C61	120.65(13)
C56–C57–C61	118.31(13)
C59–C58–C57	117.82(14)
C59–C58–H58	121.1
C57–C58–H58	121.1
C58–C59–C60	120.80(14)
C58–C59–C62	120.74(14)
C60–C59–C62	118.44(14)
C59–C60–C55	122.51(14)
C59–C60–H60	118.7
C55–C60–H60	118.7
F3–C61–F1	106.47(12)
F3–C61–F2	106.48(12)
F1–C61–F2	105.62(13)
F3–C61–C57	113.27(13)
F1–C61–C57	112.59(12)
F2–C61–C57	111.87(12)
F4–C62–F5	106.46(13)
F4–C62–F6	106.38(13)
F5–C62–F6	105.52(14)
F4–C62–C59	113.13(14)
F5–C62–C59	112.90(13)
F6–C62–C59	111.89(13)
C68–C63–C64	115.74(13)
C68–C63–B1	124.85(13)
C64–C63–B1	119.38(13)
C65–C64–C63	122.45(14)
C65–C64–H64	118.8
C63–C64–H64	118.8
C64–C65–C66	120.63(14)
C64–C65–C69	120.43(14)
C66–C65–C69	118.94(14)
C67–C66–C65	117.92(14)
C67–C66–H66	121.0
C65–C66–H66	121.0
C66–C67–C68	121.24(15)
C66–C67–C70	119.68(15)
C68–C67–C70	119.08(15)
C63–C68–C67	121.93(14)
C63–C68–H68	119.0
C67–C68–H68	119.0
F8–C69–F7	106.60(15)
F8–C69–F9	105.95(15)
F7–C69–F9	105.07(14)
F8–C69–C65	113.60(13)
F7–C69–C65	112.26(14)
F9–C69–C65	112.72(14)
F11A–C70–F10A	110.9(5)
F10–C70–F11	114.5(6)
F11A–C70–F12A	102.5(3)
F10A–C70–F12A	99.6(5)

F10–C70–F12	102.0(3)
F11–C70–F12	102.0(5)
F11A–C70–C67	117.3(2)
F10–C70–C67	117.8(2)
F10A–C70–C67	116.9(6)
F11–C70–C67	111.5(6)
F12A–C70–C67	106.9(2)
F12–C70–C67	107.0(2)
C76–C71–C72	115.54(13)
C76–C71–B1	124.91(13)
C72–C71–B1	119.38(12)
C73–C72–C71	122.76(14)
C73–C72–H72	118.6
C71–C72–H72	118.6
C72–C73–C74	120.59(14)
C72–C73–C77	118.86(14)
C74–C73–C77	120.54(13)
C75–C74–C73	117.75(14)
C75–C74–H74	121.1
C73–C74–H74	121.1
C74–C75–C76	121.30(14)
C74–C75–C78	119.91(14)
C76–C75–C78	118.78(14)
C71–C76–C75	122.03(14)
C71–C76–H76	119.0
C75–C76–H76	119.0
F13–C77–F15	106.12(13)
F13–C77–F14	105.54(13)
F15–C77–F14	106.73(13)
F13–C77–C73	112.40(13)
F15–C77–C73	113.17(14)
F14–C77–C73	112.33(13)
F18–C78–F17	108.1(2)
F18–C78–F16	105.8(3)
F17–C78–F16	105.06(18)
F18A–C78–F17A	105.9(11)
F18A–C78–F16A	110.6(7)
F17A–C78–F16A	98.4(9)
F18A–C78–C75	117.9(8)
F18–C78–C75	112.9(3)
F17–C78–C75	112.84(16)
F16–C78–C75	111.7(2)
F17A–C78–C75	110.9(11)
F16A–C78–C75	111.3(4)
C84–C79–C80	115.50(13)
C84–C79–B1	123.96(13)
C80–C79–B1	120.40(13)
C81–C80–C79	122.59(14)
C81–C80–H80	118.7
C79–C80–H80	118.7
C82–C81–C80	120.55(14)
C82–C81–C85	120.16(14)
C80–C81–C85	119.29(14)
C83–C82–C81	118.08(14)
C83–C82–H82	121.0
C81–C82–H82	121.0
C82–C83–C84	121.22(14)
C82–C83–C86	119.55(14)
C84–C83–C86	119.22(14)
C83–C84–C79	122.02(14)
C83–C84–H84	119.0
C79–C84–H84	119.0
F20–C85–F21	106.62(15)
F20–C85–F19	105.30(15)
F21–C85–F19	106.76(15)
F20–C85–C81	112.45(14)
F21–C85–C81	112.96(14)
F19–C85–C81	112.21(14)
F22A–C86–F24A	108.9(8)
F24–C86–F22	110.0(8)
F24–C86–F23	107.2(3)
F22–C86–F23	103.3(6)
F22A–C86–F23A	102.3(5)
F24A–C86–F23A	102.2(8)
F24–C86–C83	115.6(4)
F22A–C86–C83	116.7(4)
F24A–C86–C83	116.8(8)
F22–C86–C83	111.0(7)
F23–C86–C83	109.1(3)
F23A–C86–C83	107.9(4)
C63–B1–C79	105.11(11)
C63–B1–C55	110.86(12)
C79–B1–C55	111.90(12)
C63–B1–C71	112.15(12)
C79–B1–C71	112.14(12)
C55–B1–C71	104.84(11)

Symmetry transformations used to generate equivalent atoms:
#1: 2-X, 2-Y, 1-Z; #2: 1-X, 2-Y, 2-Z;

Table S14: Torsion angles for compound **3**.

**Atom–Atom–Atom–Atom**	**Torsion Angle [°]**
Cu1–N1–C1–N2	−177.05(12)
Cu1–N1–C1–N3	4.0(3)
C2–N2–C1–N1	−178.78(16)
C4–N2–C1–N1	−10.9(2)
C2–N2–C1–N3	0.35(17)
C4–N2–C1–N3	168.22(13)
C3–N3–C1–N1	179.24(16)
C16–N3–C1–N1	4.3(3)
C3–N3–C1–N2	0.09(17)
C16–N3–C1–N2	−174.84(14)
C1–N2–C2–C3	−0.68(18)

C4–N2–C2–C3	−167.12(15)
N2–C2–C3–N3	0.72(19)
C1–N3–C3–C2	−0.52(19)
C16–N3–C3–C2	174.11(15)
C1–N2–C4–C5	−82.31(18)
C2–N2–C4–C5	82.8(2)
C1–N2–C4–C9	92.19(17)
C2–N2–C4–C9	−102.67(19)
C9–C4–C5–C6	−1.8(2)
N2–C4–C5–C6	172.46(14)
C9–C4–C5–C10	179.14(15)
N2–C4–C5–C10	−6.6(2)
C4–C5–C6–C7	−0.3(2)
C10–C5–C6–C7	178.75(16)
C5–C6–C7–C8	1.9(3)
C6–C7–C8–C9	−1.3(3)
C7–C8–C9–C4	−0.7(2)
C7–C8–C9–C13	178.59(15)
C5–C4–C9–C8	2.3(2)
N2–C4–C9–C8	−171.91(13)
C5–C4–C9–C13	−176.96(14)
N2–C4–C9–C13	8.8(2)
C6–C5–C10–C11	63.8(2)
C4–C5–C10–C11	−117.18(18)
C6–C5–C10–C12	−60.5(2)
C4–C5–C10–C12	118.52(17)
C8–C9–C13–C15	60.7(2)
C4–C9–C13–C15	−120.10(19)
C8–C9–C13–C14	−62.8(2)
C4–C9–C13–C14	116.41(18)
C1–N3–C16–C17	−99.37(18)
C3–N3–C16–C17	86.7(2)
C1–N3–C16–C21	78.08(19)
C3–N3–C16–C21	−95.83(19)
C21–C16–C17–C18	−1.1(2)
N3–C16–C17–C18	176.22(13)
C21–C16–C17–C22	178.60(14)
N3–C16–C17–C22	−4.1(2)
C16–C17–C18–C19	0.9(2)
C22–C17–C18–C19	−178.79(15)
C17–C18–C19–C20	0.3(2)
C18–C19–C20–C21	−1.5(2)
C19–C20–C21–C16	1.3(2)
C19–C20–C21–C25	−177.45(15)
C17–C16–C21–C20	0.0(2)
N3–C16–C21–C20	−177.32(13)
C17–C16–C21–C25	178.75(14)
N3–C16–C21–C25	1.4(2)
C16–C17–C22–C23A	−144.3(9)
C18–C17–C22–C23A	35.4(9)
C16–C17–C22–C24A	92.1(8)
C18–C17–C22–C24A	−88.2(8)
C16–C17–C22–C24	124.3(3)
C18–C17–C22–C24	−56.0(3)
C16–C17–C22–C23	−112.8(3)

C18–C17–C22–C23	66.9(3)
C20–C21–C25–C27	24.4(2)
C16–C21–C25–C27	−154.26(15)
C20–C21–C25–C26	−100.58(18)
C16–C21–C25–C26	80.74(18)
Cu2–N4–C28–N5	−6.9(2)
Cu2–N4–C28–N6	173.12(11)
C29–N5–C28–N4	179.48(14)
C31–N5–C28–N4	3.4(2)
C29–N5–C28–N6	−0.54(16)
C31–N5–C28–N6	−176.66(13)
C30–N6–C28–N4	−179.70(15)
C43–N6–C28–N4	1.0(2)
C30–N6–C28–N5	0.31(16)
C43–N6–C28–N5	−179.00(12)
C28–N5–C29–C30	0.57(18)
C31–N5–C29–C30	176.60(14)
N5–C29–C30–N6	−0.36(18)
C28–N6–C30–C29	0.04(18)
C43–N6–C30–C29	179.31(14)
C28–N5–C31–C36	90.67(18)
C29–N5–C31–C36	−84.78(19)
C28–N5–C31–C32	−88.45(18)
C29–N5–C31–C32	96.10(18)
C36–C31–C32–C33	3.5(2)
N5–C31–C32–C33	−177.39(13)
C36–C31–C32–C37	−175.55(14)
N5–C31–C32–C37	3.5(2)
C31–C32–C33–C34	−0.6(2)
C37–C32–C33–C34	178.53(15)
C32–C33–C34–C35	−1.7(2)
C33–C34–C35–C36	1.1(2)
C32–C31–C36–C35	−4.1(2)
N5–C31–C36–C35	176.85(13)
C32–C31–C36–C40	176.13(14)
N5–C31–C36–C40	−3.0(2)
C34–C35–C36–C31	1.6(2)
C34–C35–C36–C40	−178.55(15)
C33–C32–C37–C39	−31.7(2)
C31–C32–C37–C39	147.34(15)
C33–C32–C37–C38	92.85(19)
C31–C32–C37–C38	−88.11(18)
C31–C36–C40–C42	−135.90(16)
C35–C36–C40–C42	44.3(2)
C31–C36–C40–C41	99.34(18)
C35–C36–C40–C41	−80.46(19)
C28–N6–C43–C48	78.49(17)
C30–N6–C43–C48	−100.69(18)
C28–N6–C43–C44	−99.26(16)
C30–N6–C43–C44	81.56(19)
C48–C43–C44–C45	0.2(2)
N6–C43–C44–C45	177.82(13)
C48–C43–C44–C49	−176.38(14)
N6–C43–C44–C49	1.3(2)
C43–C44–C45–C46	−0.9(2)

C49–C44–C45–C46	175.64(15)
C44–C45–C46–C47	0.9(3)
C45–C46–C47–C48	0.0(2)
C46–C47–C48–C43	−0.7(2)
C46–C47–C48–C52	178.98(15)
C44–C43–C48–C47	0.6(2)
N6–C43–C48–C47	−177.00(13)
C44–C43–C48–C52	−179.06(14)
N6–C43–C48–C52	3.3(2)
C45–C44–C49–C50	31.7(2)
C43–C44–C49–C50	−151.88(15)
C45–C44–C49–C51	−91.58(18)
C43–C44–C49–C51	84.84(18)
C47–C48–C52–C53A	−92.2(11)
C43–C48–C52–C53A	87.5(11)
C47–C48–C52–C54A	37.9(10)
C43–C48–C52–C54A	−142.5(10)
C47–C48–C52–C54	58.9(3)
C43–C48–C52–C54	−121.4(3)
C47–C48–C52–C53	−64.3(4)
C43–C48–C52–C53	115.4(3)
C60–C55–C56–C57	−1.6(2)
B1–C55–C56–C57	−178.80(13)
C55–C56–C57–C58	1.6(2)
C55–C56–C57–C61	−175.40(13)
C56–C57–C58–C59	−0.7(2)
C61–C57–C58–C59	176.20(13)
C57–C58–C59–C60	0.0(2)
C57–C58–C59–C62	−178.50(13)
C58–C59–C60–C55	0.0(2)
C62–C59–C60–C55	178.48(14)
C56–C55–C60–C59	0.8(2)
B1–C55–C60–C59	177.88(13)
C58–C57–C61–F3	8.57(19)
C56–C57–C61–F3	−174.40(12)
C58–C57–C61–F1	129.46(14)
C56–C57–C61–F1	−53.52(18)
C58–C57–C61–F2	−111.78(15)
C56–C57–C61–F2	65.25(17)
C58–C59–C62–F4	6.3(2)
C60–C59–C62–F4	−172.19(14)
C58–C59–C62–F5	−114.66(16)
C60–C59–C62–F5	66.82(19)
C58–C59–C62–F6	126.47(15)
C60–C59–C62–F6	−52.05(19)
C68–C63–C64–C65	3.2(2)
B1–C63–C64–C65	−178.87(13)
C63–C64–C65–C66	−1.6(2)
C63–C64–C65–C69	177.99(14)
C64–C65–C66–C67	−1.1(2)
C69–C65–C66–C67	179.27(15)
C65–C66–C67–C68	2.1(2)
C65–C66–C67–C70	−178.41(16)
C64–C63–C68–C67	−2.2(2)
B1–C63–C68–C67	179.98(14)

C66–C67–C68–C63	−0.4(2)
C70–C67–C68–C63	−179.88(16)
C64–C65–C69–F8	11.4(2)
C66–C65–C69–F8	−169.01(15)
C64–C65–C69–F7	−109.63(17)
C66–C65–C69–F7	69.9(2)
C64–C65–C69–F9	131.94(16)
C66–C65–C69–F9	−48.5(2)
C66–C67–C70–F11A	−114.7(5)
C68–C67–C70–F11A	64.7(5)
C66–C67–C70–F10	169.6(5)
C68–C67–C70–F10	−10.9(5)
C66–C67–C70–F10A	20.5(5)
C68–C67–C70–F10A	−160.0(5)
C66–C67–C70–F11	34.2(5)
C68–C67–C70–F11	−146.3(5)
C66–C67–C70–F12A	131.0(3)
C68–C67–C70–F12A	−49.5(3)
C66–C67–C70–F12	−76.5(3)
C68–C67–C70–F12	103.0(3)
C76–C71–C72–C73	−1.7(2)
B1–C71–C72–C73	−177.17(13)
C71–C72–C73–C74	1.0(2)
C71–C72–C73–C77	−177.79(13)
C72–C73–C74–C75	0.4(2)
C77–C73–C74–C75	179.14(14)
C73–C74–C75–C76	−0.9(2)
C73–C74–C75–C78	178.09(14)
C72–C71–C76–C75	1.1(2)
B1–C71–C76–C75	176.31(13)
C74–C75–C76–C71	0.1(2)
C78–C75–C76–C71	−178.89(14)
C72–C73–C77–F13	70.27(18)
C74–C73–C77–F13	−108.51(16)
C72–C73–C77–F15	−169.54(13)
C74–C73–C77–F15	11.7(2)
C72–C73–C77–F14	−48.58(19)
C74–C73–C77–F14	132.64(15)
C74–C75–C78–F18A	109.1(8)
C76–C75–C78–F18A	−71.8(8)
C74–C75–C78–F18	−110.8(3)
C76–C75–C78–F18	68.3(3)
C74–C75–C78–F17	12.1(3)
C76–C75–C78–F17	−168.8(3)
C74–C75–C78–F16	130.2(3)
C76–C75–C78–F16	−50.7(3)
C74–C75–C78–F17A	−128.6(8)
C76–C75–C78–F17A	50.4(8)
C74–C75–C78–F16A	−20.2(10)
C76–C75–C78–F16A	158.8(10)
C84–C79–C80–C81	2.0(2)
B1–C79–C80–C81	177.84(14)
C79–C80–C81–C82	−0.6(2)
C79–C80–C81–C85	178.98(14)
C80–C81–C82–C83	−1.2(2)

C85–C81–C82–C83	179.24(15)
C81–C82–C83–C84	1.4(2)
C81–C82–C83–C86	−179.74(16)
C82–C83–C84–C79	0.1(2)
C86–C83–C84–C79	−178.71(15)
C80–C79–C84–C83	−1.8(2)
B1–C79–C84–C83	−177.44(14)
C82–C81–C85–F20	116.38(17)
C80–C81–C85–F20	−63.2(2)
C82–C81–C85–F21	−4.4(2)
C80–C81–C85–F21	176.08(15)
C82–C81–C85–F19	−125.12(17)
C80–C81–C85–F19	55.3(2)
C82–C83–C86–F24	−88.5(7)
C84–C83–C86–F24	90.3(7)
C82–C83–C86–F22A	4.0(10)
C84–C83–C86–F22A	−177.1(10)
C82–C83–C86–F24A	135.3(8)
C84–C83–C86–F24A	−45.8(8)
C82–C83–C86–F22	145.4(7)
C84–C83–C86–F22	−35.8(7)
C82–C83–C86–F23	32.3(5)
C84–C83–C86–F23	−148.9(4)
C82–C83–C86–F23A	−110.4(5)
C84–C83–C86–F23A	68.5(5)
C68–C63–B1–C79	108.59(15)
C64–C63–B1–C79	−69.16(16)
C68–C63–B1–C55	−130.32(14)
C64–C63–B1–C55	51.93(17)
C68–C63–B1–C71	−13.50(19)
C64–C63–B1–C71	168.74(12)
C84–C79–B1–C63	103.68(15)
C80–C79–B1–C63	−71.76(16)
C84–C79–B1–C55	−16.73(19)
C80–C79–B1–C55	167.83(13)
C84–C79–B1–C71	−134.21(14)
C80–C79–B1–C71	50.35(18)
C56–C55–B1–C63	−169.58(12)
C60–C55–B1–C63	13.47(19)
C56–C55–B1–C79	−52.59(17)
C60–C55–B1–C79	130.46(14)
C56–C55–B1–C71	69.19(16)
C60–C55–B1–C71	−107.76(15)
C76–C71–B1–C63	133.28(14)
C72–C71–B1–C63	−51.72(17)
C76–C71–B1–C79	15.3(2)
C72–C71–B1–C79	−169.72(12)
C76–C71–B1–C55	−106.35(15)
C72–C71–B1–C55	68.66(16)

Table S15: Hydrogen bonds for compound **3**.

D–H···A [Å]	d(D–H) [Å]	d(H···A) [Å]	d(D···A) [Å]	<(DHA) [°]
C3–H3···F23A^b	0.95	2.63	3.198(8)	119.1
C30–H30···F3[#1]	0.95	2.44	3.2808(18)	146.7
C30–H30···F7	0.95	2.64	3.2673(18)	124.2

Symmetry transformations used to generate equivalent atoms:
#1: -1+X, +Y, +Z;

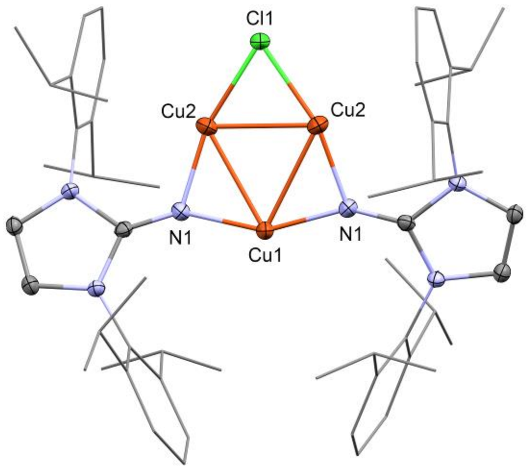

Table S16: Crystal data and structure refinement for [$Cu_3(DippIm)_2Cl$] (**4**).

CCDC number	2363596
Empirical formula	$C_{54}H_{72}ClCu_2N_6$
Formula weight	967.70
Temperature [K]	100(2)
Crystal system	monoclinic
Space group (number)	$C2/c$ (15)
a [Å]	24.1413(12)
b [Å]	11.7772(5)
c [Å]	18.2952(8)
α [°]	90
β [°]	103.963(2)
γ [°]	90
Volume [Å^3]	5047.9(4)
Z	4
ρ_{calc} [gcm^{-3}]	1.273
μ [mm^{-1}]	0.937
F(000)	2052
Crystal size [mm^3]	0.124×0.227×0.310
Crystal colour	colourless
Crystal shape	plate
Radiation	MoK_α (λ=0.71073 Å)
2θ range [°]	4.28 to 50.05 (0.84 Å)
Index ranges	−28 ≤ h ≤ 28<br>−14 ≤ k ≤ 14<br>−21 ≤ l ≤ 21
Reflections collected	48604
Independent reflections	4444<br>R_{int} = 0.0704<br>R_{sigma} = 0.0297
Completeness to θ = 25.027°	99.9 %
Data / Restraints / Parameters	4444 / 0 / 298
Goodness-of-fit on F^2	1.070
Final *R* indexes [I≥2σ(I)]	R_1 = 0.0301<br>wR_2 = 0.0732
Final *R* indexes [all data]	R_1 = 0.0364<br>wR_2 = 0.0750
Largest peak/hole [eÅ^{-3}]	0.37/−0.49

Table S17: Atomic coordinates and Ueq [Å^2] for compound **4**.

**Atom**	***x***	***y***	***z***	U_{eq}
Cu1	0.500000	0.41183(2)	0.750000	0.01699(10)
Cu2	0.55241(2)	0.59859(4)	0.75953(3)	0.02318(12)
Cl1	0.500000	0.74914(5)	0.750000	0.03130(18)
N1	0.57422(6)	0.44882(12)	0.74934(9)	0.0191(3)
N2	0.66871(6)	0.48906(12)	0.73879(8)	0.0150(3)
N3	0.63706(6)	0.31676(12)	0.70553(8)	0.0146(3)
C1	0.62011(7)	0.41951(14)	0.73283(9)	0.0144(4)
C2	0.71210(7)	0.43010(15)	0.71725(10)	0.0163(4)
H2	0.748813	0.458935	0.717039	0.020
C3	0.69287(7)	0.32566(15)	0.69691(10)	0.0167(4)
H3	0.713630	0.267258	0.679520	0.020
C4	0.67204(7)	0.59872(14)	0.77256(10)	0.0153(4)
C5	0.68381(8)	0.60706(14)	0.85161(10)	0.0179(4)
C6	0.68123(8)	0.71392(15)	0.88300(10)	0.0209(4)
H6	0.688889	0.721672	0.936181	0.025
C7	0.66768(8)	0.80902(15)	0.83791(11)	0.0208(4)
H7	0.664391	0.880810	0.860162	0.025
C8	0.65890(7)	0.80019(15)	0.76061(10)	0.0190(4)
H8	0.651048	0.866608	0.730411	0.023
C9	0.66139(7)	0.69523(15)	0.72634(10)	0.0165(4)
C10	0.69993(9)	0.50479(16)	0.90285(10)	0.0255(4)
H10	0.701648	0.437104	0.870475	0.031
C11	0.75940(10)	0.52239(19)	0.95465(12)	0.0404(6)
H11A	0.771201	0.453338	0.984195	0.061
H11B	0.758364	0.586126	0.988735	0.061
H11C	0.786687	0.538972	0.924168	0.061
C12	0.65566(11)	0.48089(19)	0.94791(13)	0.0419(6)
H12A	0.666085	0.411463	0.977567	0.063
H12B	0.618061	0.471090	0.913324	0.063
H12C	0.654382	0.544766	0.981799	0.063
C13	0.65258(8)	0.68518(15)	0.64143(10)	0.0193(4)
H13	0.676598	0.620539	0.631452	0.023
C14	0.67160(8)	0.79165(16)	0.60629(11)	0.0249(4)
H14A	0.673424	0.775217	0.554409	0.037
H14B	0.709404	0.814965	0.635679	0.037
H14C	0.644174	0.853002	0.606262	0.037
C15	0.59056(8)	0.65753(17)	0.60315(11)	0.0269(4)
H15A	0.586575	0.648506	0.548878	0.040
H15B	0.565878	0.719494	0.612007	0.040
H15C	0.579422	0.586844	0.624008	0.040
C16	0.60481(7)	0.21310(14)	0.69320(10)	0.0147(4)
C17	0.58429(7)	0.17481(15)	0.61895(10)	0.0174(4)
C18	0.55864(8)	0.06792(15)	0.60900(11)	0.0204(4)
H18	0.545706	0.038093	0.559564	0.025
C19	0.55171(8)	0.00463(15)	0.66987(10)	0.0206(4)
H19	0.534044	−0.067884	0.661859	0.025
C20	0.57036(8)	0.04630(15)	0.74228(10)	0.0190(4)
H20	0.564373	0.002966	0.783494	0.023
C21	0.59787(7)	0.15119(15)	0.75568(10)	0.0169(4)

C22	0.58650(8)	0.25037(16)	0.55233(10)	0.0224(4)
H22	0.625172	0.286156	0.562469	0.027
C23	0.57660(10)	0.18684(19)	0.47745(11)	0.0331(5)
H23A	0.580988	0.239475	0.437746	0.050
H23B	0.537941	0.155051	0.464775	0.050
H23C	0.604509	0.125266	0.481827	0.050
C24	0.54213(9)	0.34549(17)	0.54645(11)	0.0296(5)
H24A	0.544724	0.397086	0.505387	0.044
H24B	0.549544	0.387758	0.593969	0.044
H24C	0.503792	0.312332	0.536257	0.044
C25	0.62117(8)	0.19526(15)	0.83516(10)	0.0193(4)
H25	0.618843	0.280004	0.832853	0.023
C26	0.68421(8)	0.16387(17)	0.86298(11)	0.0266(4)
H26A	0.699672	0.198561	0.912387	0.040
H26B	0.705353	0.191803	0.827134	0.040
H26C	0.688006	0.081143	0.867258	0.040
C27	0.58673(9)	0.15603(19)	0.89001(11)	0.0330(5)
H27A	0.600312	0.195275	0.938248	0.050
H27B	0.591380	0.073908	0.897765	0.050
H27C	0.546297	0.173631	0.869348	0.050

U_{eq} is defined as 1/3 of the trace of the orthogonalized U_{ij} tensor.

Table S17: Bond lengths and angles for compound **4**.

**Atom–Atom**	**Length [Å]**
Cu1–N1$^{\#1}$	1.8468(15)
Cu1–N1	1.8469(15)
Cu1–Cu2	2.5224(5)
Cu1–Cu2$^{\#1}$	2.5225(5)
Cu2–N1	1.8629(15)
Cu2–Cl1	2.1607(7)
Cu2–Cu2$^{\#1}$	2.4698(9)
N1–C1	1.264(2)
N2–C2	1.391(2)
N2–C1	1.413(2)
N2–C4	1.426(2)
N3–C3	1.398(2)
N3–C1	1.407(2)
N3–C16	1.436(2)
C2–C3	1.335(2)
C2–H2	0.9500
C3–H3	0.9500
C4–C9	1.403(2)
C4–C5	1.408(2)
C5–C6	1.391(3)
C5–C10	1.518(2)
C6–C7	1.383(3)
C6–H6	0.9500
C7–C8	1.382(3)
C7–H7	0.9500
C8–C9	1.394(3)
C8–H8	0.9500
C9–C13	1.521(2)
C10–C12	1.525(3)
C10–C11	1.532(3)
C10–H10	1.0000
C11–H11A	0.9800
C11–H11B	0.9800
C11–H11C	0.9800
C12–H12A	0.9800
C12–H12B	0.9800
C12–H12C	0.9800
C13–C15	1.527(3)
C13–C14	1.529(3)
C13–H13	1.0000
C14–H14A	0.9800
C14–H14B	0.9800
C14–H14C	0.9800
C15–H15A	0.9800
C15–H15B	0.9800
C15–H15C	0.9800
C16–C21	1.399(3)
C16–C17	1.403(2)
C17–C18	1.395(2)
C17–C22	1.520(3)
C18–C19	1.383(3)
C18–H18	0.9500
C19–C20	1.382(3)
C19–H19	0.9500
C20–C21	1.396(2)
C20–H20	0.9500
C21–C25	1.518(2)

C22–C23	1.528(3)
C22–C24	1.536(3)
C22–H22	1.0000
C23–H23A	0.9800
C23–H23B	0.9800
C23–H23C	0.9800
C24–H24A	0.9800
C24–H24B	0.9800
C24–H24C	0.9800
C25–C27	1.522(3)
C25–C26	1.529(3)
C25–H25	1.0000
C26–H26A	0.9800
C26–H26B	0.9800
C26–H26C	0.9800
C27–H27A	0.9800
C27–H27B	0.9800
C27–H27C	0.9800
**Atom–Atom–Atom**	**Angle [°]**
N1$^{\#1}$–Cu1–N1	152.71(9)
N1$^{\#1}$–Cu1–Cu2	105.37(5)
N1–Cu1–Cu2	47.43(5)
N1$^{\#1}$–Cu1–Cu2$^{\#1}$	47.43(5)
N1–Cu1–Cu2$^{\#1}$	105.37(5)
Cu2–Cu1–Cu2$^{\#1}$	58.62(2)
N1–Cu2–Cl1	160.56(5)
N1–Cu2–Cu2$^{\#1}$	106.89(5)
Cl1–Cu2–Cu2$^{\#1}$	55.144(16)
N1–Cu2–Cu1	46.89(5)
Cl1–Cu2–Cu1	115.83(2)
Cu2$^{\#1}$–Cu2–Cu1	60.689(11)
Cu2–Cl1–Cu2$^{\#1}$	69.71(3)
C1–N1–Cu1	147.61(13)
C1–N1–Cu2	124.57(12)
Cu1–N1–Cu2	85.68(6)
C2–N2–C1	110.91(14)
C2–N2–C4	127.41(14)
C1–N2–C4	121.16(14)
C3–N3–C1	110.26(13)
C3–N3–C16	123.15(14)
C1–N3–C16	126.42(14)
N1–C1–N3	131.77(16)
N1–C1–N2	125.60(15)
N3–C1–N2	102.63(14)
C3–C2–N2	107.69(15)
C3–C2–H2	126.2
N2–C2–H2	126.2
C2–C3–N3	108.51(15)
C2–C3–H3	125.7
N3–C3–H3	125.7
C9–C4–C5	121.64(15)
C9–C4–N2	119.33(15)
C5–C4–N2	118.98(15)
C6–C5–C4	117.97(16)
C6–C5–C10	119.55(16)
C4–C5–C10	122.47(15)
C7–C6–C5	120.95(17)
C7–C6–H6	119.5
C5–C6–H6	119.5
C8–C7–C6	120.34(16)
C8–C7–H7	119.8
C6–C7–H7	119.8
C7–C8–C9	120.95(16)
C7–C8–H8	119.5
C9–C8–H8	119.5
C8–C9–C4	117.97(16)
C8–C9–C13	121.19(16)
C4–C9–C13	120.84(15)
C5–C10–C12	111.81(17)
C5–C10–C11	109.70(17)
C12–C10–C11	111.41(19)
C5–C10–H10	107.9
C12–C10–H10	107.9
C11–C10–H10	107.9
C10–C11–H11A	109.5
C10–C11–H11B	109.5
H11A–C11–H11B	109.5
C10–C11–H11C	109.5
H11A–C11–H11C	109.5
H11B–C11–H11C	109.5
C10–C12–H12A	109.5
C10–C12–H12B	109.5
H12A–C12–H12B	109.5
C10–C12–H12C	109.5
H12A–C12–H12C	109.5
H12B–C12–H12C	109.5
C9–C13–C15	111.39(15)
C9–C13–C14	112.55(15)
C15–C13–C14	110.04(15)
C9–C13–H13	107.5
C15–C13–H13	107.5
C14–C13–H13	107.5
C13–C14–H14A	109.5
C13–C14–H14B	109.5
H14A–C14–H14B	109.5
C13–C14–H14C	109.5
H14A–C14–H14C	109.5
H14B–C14–H14C	109.5
C13–C15–H15A	109.5
C13–C15–H15B	109.5
H15A–C15–H15B	109.5
C13–C15–H15C	109.5
H15A–C15–H15C	109.5
H15B–C15–H15C	109.5
C21–C16–C17	122.64(16)
C21–C16–N3	118.81(15)
C17–C16–N3	118.45(15)
C18–C17–C16	117.26(16)
C18–C17–C22	121.58(16)

C16–C17–C22	121.03(16)
C19–C18–C17	121.14(17)
C19–C18–H18	119.4
C17–C18–H18	119.4
C20–C19–C18	120.37(16)
C20–C19–H19	119.8
C18–C19–H19	119.8
C19–C20–C21	120.91(17)
C19–C20–H20	119.5
C21–C20–H20	119.5
C20–C21–C16	117.60(16)
C20–C21–C25	121.42(16)
C16–C21–C25	120.95(15)
C17–C22–C23	113.82(16)
C17–C22–C24	108.92(15)
C23–C22–C24	109.80(16)
C17–C22–H22	108.0
C23–C22–H22	108.0
C24–C22–H22	108.0
C22–C23–H23A	109.5
C22–C23–H23B	109.5
H23A–C23–H23B	109.5
C22–C23–H23C	109.5
H23A–C23–H23C	109.5
H23B–C23–H23C	109.5
C22–C24–H24A	109.5
C22–C24–H24B	109.5
H24A–C24–H24B	109.5
C22–C24–H24C	109.5
H24A–C24–H24C	109.5
H24B–C24–H24C	109.5
C21–C25–C27	113.12(15)
C21–C25–C26	110.20(15)
C27–C25–C26	111.44(16)
C21–C25–H25	107.3
C27–C25–H25	107.3
C26–C25–H25	107.3
C25–C26–H26A	109.5
C25–C26–H26B	109.5
H26A–C26–H26B	109.5
C25–C26–H26C	109.5
H26A–C26–H26C	109.5
H26B–C26–H26C	109.5
C25–C27–H27A	109.5
C25–C27–H27B	109.5
H27A–C27–H27B	109.5
C25–C27–H27C	109.5
H27A–C27–H27C	109.5
H27B–C27–H27C	109.5

Symmetry transformations used to generate equivalent atoms:
#1: 1-X, +Y, 1.5-Z;

Table S18: Torsion angles for compound **4**.

Atom–Atom–Atom–Atom	Torsion Angle [°]
N1$^{\#1}$–Cu1–N1–C1	−155.1(3)
Cu2–Cu1–N1–C1	−160.6(3)
Cu2$^{\#1}$–Cu1–N1–C1	−150.9(2)
N1$^{\#1}$–Cu1–N1–Cu2	5.47(4)
Cu2$^{\#1}$–Cu1–N1–Cu2	9.65(6)
Cl1–Cu2–N1–C1	136.69(15)
Cu2$^{\#1}$–Cu2–N1–C1	157.56(14)
Cu1–Cu2–N1–C1	167.50(18)
Cl1–Cu2–N1–Cu1	−30.81(19)
Cu2$^{\#1}$–Cu2–N1–Cu1	−9.94(6)
Cu1–N1–C1–N3	−6.1(4)
Cu2–N1–C1–N3	−162.35(14)
Cu1–N1–C1–N2	174.07(16)
Cu2–N1–C1–N2	17.8(3)
C3–N3–C1–N1	−179.17(18)
C16–N3–C1–N1	−3.8(3)
C3–N3–C1–N2	0.68(17)
C16–N3–C1–N2	176.07(15)
C2–N2–C1–N1	179.05(17)
C4–N2–C1–N1	6.7(3)
C2–N2–C1–N3	−0.81(18)
C4–N2–C1–N3	−173.12(14)
C1–N2–C2–C3	0.67(19)
C4–N2–C2–C3	172.38(16)
N2–C2–C3–N3	−0.21(19)
C1–N3–C3–C2	−0.31(19)
C16–N3–C3–C2	−175.89(15)
C2–N2–C4–C9	88.1(2)
C1–N2–C4–C9	−100.93(19)
C2–N2–C4–C5	−94.3(2)
C1–N2–C4–C5	76.6(2)
C9–C4–C5–C6	3.8(3)
N2–C4–C5–C6	−173.69(16)
C9–C4–C5–C10	−175.18(17)
N2–C4–C5–C10	7.3(3)
C4–C5–C6–C7	−0.2(3)
C10–C5–C6–C7	178.84(17)
C5–C6–C7–C8	−2.9(3)
C6–C7–C8–C9	2.4(3)
C7–C8–C9–C4	1.1(3)
C7–C8–C9–C13	−179.13(16)
C5–C4–C9–C8	−4.3(3)
N2–C4–C9–C8	173.22(15)
C5–C4–C9–C13	175.99(16)
N2–C4–C9–C13	−6.5(2)
C6–C5–C10–C12	64.6(2)
C4–C5–C10–C12	−116.4(2)
C6–C5–C10–C11	−59.5(2)
C4–C5–C10–C11	119.45(19)

C8–C9–C13–C15	−94.5(2)
C4–C9–C13–C15	85.2(2)
C8–C9–C13–C14	29.6(2)
C4–C9–C13–C14	−150.63(16)
C3–N3–C16–C21	103.91(19)
C1–N3–C16–C21	−70.9(2)
C3–N3–C16–C17	−72.5(2)
C1–N3–C16–C17	112.62(19)
C21–C16–C17–C18	−3.2(3)
N3–C16–C17–C18	173.07(15)
C21–C16–C17–C22	172.81(16)
N3–C16–C17–C22	−10.9(2)
C16–C17–C18–C19	2.6(3)
C22–C17–C18–C19	−173.42(17)
C17–C18–C19–C20	−0.2(3)
C18–C19–C20–C21	−1.8(3)
C19–C20–C21–C16	1.2(3)
C19–C20–C21–C25	−176.91(16)
C17–C16–C21–C20	1.4(3)
N3–C16–C21–C20	−174.91(15)
C17–C16–C21–C25	179.48(16)
N3–C16–C21–C25	3.2(2)
C18–C17–C22–C23	−18.6(2)
C16–C17–C22–C23	165.57(17)
C18–C17–C22–C24	104.30(19)
C16–C17–C22–C24	−71.6(2)
C20–C21–C25–C27	−31.4(2)
C16–C21–C25–C27	150.61(17)
C20–C21–C25–C26	94.1(2)
C16–C21–C25–C26	−83.9(2)

Symmetry transformations used to generate equivalent atoms:
#1: 1-X, +Y, 1.5-Z;

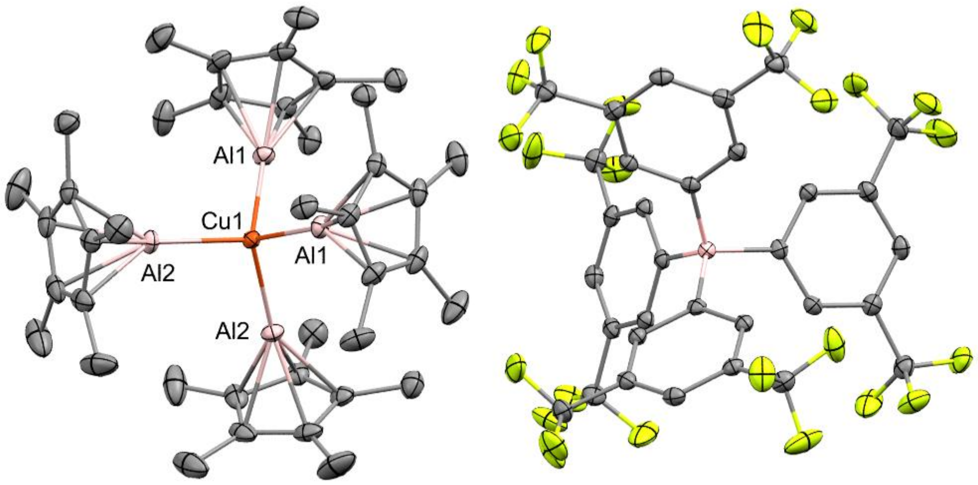

Table S19: Crystal data and structure refinement for [Cu(AlCp*)$_4$][BAr^F] (**5**).

CCDC number	2363597
Empirical formula	$C_{72}H_{72}Al_4BCuF_{24}$
Formula weight	1575.56
Temperature [K]	100(2)
Crystal system	monoclinic
Space group (number)	$P2/n$ (13)
a [Å]	15.3552(12)
b [Å]	12.7831(10)
c [Å]	19.2429(13)
α [°]	90
β [°]	98.371(2)
γ [°]	90
Volume [Å^3]	3736.9(5)
Z	2
ρ_{calc} [gcm^{-3}]	1.400
μ [mm^{-1}]	0.439
F(000)	1612
Crystal size [mm^3]	0.105×0.180×0.182
Crystal colour	white
Crystal shape	fragment
Radiation	MoK_α (λ=0.71073 Å)
2θ range [°]	4.28 to 52.80 (0.80 Å)
Index ranges	−19 ≤ h ≤ 19<br>−15 ≤ k ≤ 15<br>−24 ≤ l ≤ 24
Reflections collected	375395
Independent reflections	7642<br>R_{int} = 0.0670<br>R_{sigma} = 0.0148
Completeness to θ = 25.242°	99.9 %
Data / Restraints / Parameters	7642 / 0 / 471
Goodness-of-fit on F^2	1.063
Final *R* indexes [I≥2σ(I)]	R_1 = 0.0298<br>wR_2 = 0.0855
Final *R* indexes [all data]	R_1 = 0.0319<br>wR_2 = 0.0866
Largest peak/hole [eÅ^{-3}]	0.49/−0.45

Table S20: Atomic coordinates and Ueq [Å^2] for compound **5**.

Atom	*x*	*y*	*z*	U_{eq}
Cu1	0.250000	0.81756(2)	0.250000	0.01525(7)
Al1	0.32087(3)	0.71311(3)	0.18116(2)	0.01958(10)
Al2	0.34944(3)	0.92088(3)	0.31598(2)	0.02031(10)
F1	0.45371(6)	0.42589(9)	0.41326(6)	0.0474(3)
F2	0.54998(6)	0.37279(8)	0.49666(4)	0.0342(2)
F3	0.50912(7)	0.27221(8)	0.40965(5)	0.0374(2)
F4	0.82996(7)	0.65335(9)	0.41449(7)	0.0489(3)
F5	0.72318(8)	0.72703(7)	0.34897(5)	0.0412(2)
F6	0.71382(7)	0.69781(7)	0.45702(5)	0.0371(2)
F7	0.65701(7)	−0.08105(7)	0.15291(6)	0.0376(2)
F8	0.75736(6)	−0.01450(7)	0.10104(5)	0.0356(2)
F9	0.62780(8)	−0.04007(8)	0.04430(5)	0.0472(3)
F10	0.40054(6)	0.21737(8)	0.04968(5)	0.0342(2)
F11	0.45832(6)	0.37020(8)	0.06082(6)	0.0423(3)
F12	0.40167(6)	0.30279(8)	0.14538(5)	0.0349(2)
C1	0.35238(9)	0.64894(12)	0.08073(7)	0.0214(3)
C2	0.33122(9)	0.56432(11)	0.12363(7)	0.0213(3)
C3	0.39447(9)	0.56346(12)	0.18587(7)	0.0244(3)
C4	0.45454(9)	0.64703(13)	0.18113(7)	0.0266(3)
C5	0.42853(9)	0.70017(12)	0.11646(7)	0.0251(3)
C6	0.30530(10)	0.67483(13)	0.00880(7)	0.0284(3)
H6A	0.328509	0.631240	−0.026185	0.043
H6B	0.314493	0.748785	−0.001420	0.043
H6C	0.242197	0.661350	0.007068	0.043
C7	0.25702(10)	0.48870(12)	0.10398(8)	0.0281(3)
H7A	0.272798	0.439399	0.068855	0.042
H7B	0.203852	0.527151	0.084416	0.042
H7C	0.245941	0.450146	0.145834	0.042
C8	0.39807(12)	0.49019(14)	0.24724(8)	0.0356(4)
H8A	0.450301	0.445576	0.249457	0.053
H8B	0.345103	0.446427	0.241556	0.053
H8C	0.401141	0.530749	0.290744	0.053
C9	0.53263(11)	0.67320(16)	0.23513(9)	0.0409(4)
H9A	0.580955	0.624878	0.230363	0.061
H9B	0.516190	0.666652	0.282270	0.061
H9C	0.551568	0.745091	0.227889	0.061
C10	0.47315(11)	0.79407(15)	0.09058(9)	0.0371(4)
H10A	0.524185	0.771371	0.069253	0.056
H10B	0.492653	0.840906	0.130111	0.056
H10C	0.431716	0.831174	0.055502	0.056
C11	0.40637(11)	1.07963(12)	0.33396(8)	0.0318(3)
C12	0.47635(10)	1.00723(13)	0.33379(8)	0.0292(3)
C13	0.47353(9)	0.93581(11)	0.39044(7)	0.0230(3)
C14	0.40148(9)	0.96472(11)	0.42535(7)	0.0232(3)
C15	0.36017(10)	1.05395(12)	0.39081(8)	0.0288(3)
C16	0.38574(16)	1.16846(15)	0.28275(11)	0.0537(6)
H16A	0.438011	1.212597	0.283270	0.080
H16B	0.337429	1.210325	0.296258	0.080
H16C	0.368355	1.140229	0.235414	0.080

C17	0.54282(12)	1.00771(19)	0.28338(10)	0.0509(5)
H17A	0.584314	1.065479	0.295110	0.076
H17B	0.512397	1.016730	0.235334	0.076
H17C	0.574961	0.941244	0.286899	0.076
C18	0.53615(11)	0.84801(14)	0.41213(8)	0.0321(3)
H18A	0.569322	0.863154	0.458488	0.048
H18B	0.577046	0.840576	0.377812	0.048
H18C	0.503054	0.782824	0.414297	0.048
C19	0.37465(11)	0.90850(14)	0.48748(8)	0.0330(3)
H19A	0.413499	0.929377	0.530214	0.049
H19B	0.379229	0.832821	0.480733	0.049
H19C	0.313722	0.926650	0.492011	0.049
C20	0.28197(14)	1.11093(16)	0.41050(10)	0.0491(5)
H20A	0.302021	1.170257	0.440934	0.074
H20B	0.247373	1.063238	0.435539	0.074
H20C	0.245392	1.136531	0.367904	0.074
C21	0.70352(8)	0.39751(10)	0.30530(6)	0.0151(2)
C22	0.63367(8)	0.35936(10)	0.33807(6)	0.0157(2)
H22	0.608544	0.293352	0.324141	0.019
C23	0.60043(8)	0.41551(10)	0.39023(6)	0.0173(3)
C24	0.63408(9)	0.51340(11)	0.41162(6)	0.0186(3)
H24	0.610217	0.552499	0.446375	0.022
C25	0.70322(9)	0.55176(10)	0.38069(6)	0.0180(3)
C26	0.73783(8)	0.49453(10)	0.32933(6)	0.0163(2)
H26	0.786400	0.522414	0.309986	0.020
C27	0.52853(9)	0.37152(11)	0.42641(7)	0.0214(3)
C28	0.74248(10)	0.65683(12)	0.40064(7)	0.0244(3)
C29	0.67893(8)	0.25456(10)	0.19876(6)	0.0152(2)
C30	0.70208(8)	0.15723(10)	0.17318(6)	0.0167(2)
H30	0.759344	0.130408	0.188528	0.020
C31	0.64434(9)	0.09856(10)	0.12620(6)	0.0183(3)
C32	0.56069(9)	0.13584(11)	0.10071(6)	0.0192(3)
H32	0.520794	0.095619	0.069031	0.023
C33	0.53745(8)	0.23389(11)	0.12316(6)	0.0180(3)
C34	0.59499(8)	0.29130(10)	0.17161(6)	0.0162(2)
H34	0.576577	0.357451	0.186638	0.019
C35	0.67150(9)	−0.00807(11)	0.10564(7)	0.0243(3)
C36	0.45003(9)	0.28048(11)	0.09451(7)	0.0224(3)
B1	0.750000	0.32637(15)	0.250000	0.0140(4)

U_{eq} is defined as 1/3 of the trace of the orthogonalized U_{ij} tensor.

Table S21: Bond lengths and angles for compound **5**.

Atom–Atom	Length [Å]
Cu1–Al2[#1]	2.2635(4)
Cu1–Al2	2.2635(4)
Cu1–Al1	2.2675(4)
Cu1–Al1[#1]	2.2676(4)
Al1–C5	2.2157(14)
Al1–C1	2.2168(13)
Al1–C3	2.2169(15)
Al1–C2	2.2179(14)
Al1–C4	2.2195(14)
Al2–C14	2.2119(13)
Al2–C11	2.2167(15)
Al2–C13	2.2192(14)
Al2–C15	2.2193(15)
Al2–C12	2.2224(15)
F1–C27	1.3351(17)
F2–C27	1.3441(15)
F3–C27	1.3329(18)

F4–C28	1.3316(18)
F5–C28	1.3401(18)
F6–C28	1.3357(17)
F7–C35	1.3440(17)
F8–C35	1.3368(17)
F9–C35	1.3341(17)
F10–C36	1.3347(16)
F11–C36	1.3326(17)
F12–C36	1.3426(17)
C1–C5	1.4265(19)
C1–C2	1.427(2)
C1–C6	1.5023(19)
C2–C3	1.4280(19)
C2–C7	1.500(2)
C3–C4	1.423(2)
C3–C8	1.502(2)
C4–C5	1.423(2)
C4–C9	1.505(2)
C5–C10	1.502(2)
C6–H6A	0.9800
C6–H6B	0.9800
C6–H6C	0.9800
C7–H7A	0.9800
C7–H7B	0.9800
C7–H7C	0.9800
C8–H8A	0.9800
C8–H8B	0.9800
C8–H8C	0.9800
C9–H9A	0.9800
C9–H9B	0.9800
C9–H9C	0.9800
C10–H10A	0.9800
C10–H10B	0.9800
C10–H10C	0.9800
C11–C12	1.419(2)
C11–C15	1.426(2)
C11–C16	1.507(2)
C12–C13	1.427(2)
C12–C17	1.507(2)
C13–C14	1.424(2)
C13–C18	1.497(2)
C14–C15	1.422(2)
C14–C19	1.503(2)
C15–C20	1.499(2)
C16–H16A	0.9800
C16–H16B	0.9800
C16–H16C	0.9800
C17–H17A	0.9800
C17–H17B	0.9800
C17–H17C	0.9800
C18–H18A	0.9800
C18–H18B	0.9800
C18–H18C	0.9800
C19–H19A	0.9800
C19–H19B	0.9800

C19–H19C	0.9800
C20–H20A	0.9800
C20–H20B	0.9800
C20–H20C	0.9800
C21–C26	1.3995(18)
C21–C22	1.4081(18)
C21–B1	1.6401(16)
C22–C23	1.3900(18)
C22–H22	0.9500
C23–C24	1.3930(19)
C23–C27	1.4980(18)
C24–C25	1.3806(19)
C24–H24	0.9500
C25–C26	1.3956(18)
C25–C28	1.4991(19)
C26–H26	0.9500
C29–C34	1.3996(18)
C29–C30	1.4026(18)
C29–B1	1.6409(16)
C30–C31	1.3901(18)
C30–H30	0.9500
C31–C32	1.3907(19)
C31–C35	1.4955(19)
C32–C33	1.3892(19)
C32–H32	0.9500
C33–C34	1.3959(18)
C33–C36	1.4983(19)
C34–H34	0.9500
**Atom–Atom–Atom**	**Angle [°]**
Al2$^{\#1}$–Cu1–Al2	108.61(2)
Al2$^{\#1}$–Cu1–Al1	110.969(15)
Al2–Cu1–Al1	109.228(16)
Al2$^{\#1}$–Cu1–Al1$^{\#1}$	109.227(16)
Al2–Cu1–Al1$^{\#1}$	110.968(15)
Al1–Cu1–Al1$^{\#1}$	107.85(2)
C5–Al1–C1	37.55(5)
C5–Al1–C3	62.68(6)
C1–Al1–C3	62.72(5)
C5–Al1–C2	62.74(5)
C1–Al1–C2	37.53(5)
C3–Al1–C2	37.57(5)
C5–Al1–C4	37.43(6)
C1–Al1–C4	62.60(5)
C3–Al1–C4	37.41(6)
C2–Al1–C4	62.62(5)
C5–Al1–Cu1	146.40(5)
C1–Al1–Cu1	155.70(4)
C3–Al1–Cu1	140.30(4)
C2–Al1–Cu1	150.68(4)
C4–Al1–Cu1	138.31(4)
C14–Al2–C11	62.60(5)
C14–Al2–C13	37.50(5)
C11–Al2–C13	62.51(6)
C14–Al2–C15	37.44(5)

C11–Al2–C15	37.51(6)
C13–Al2–C15	62.58(5)
C14–Al2–C12	62.62(5)
C11–Al2–C12	37.27(6)
C13–Al2–C12	37.49(5)
C15–Al2–C12	62.53(6)
C14–Al2–Cu1	143.36(4)
C11–Al2–Cu1	147.16(4)
C13–Al2–Cu1	149.09(4)
C15–Al2–Cu1	142.32(5)
C12–Al2–Cu1	151.77(4)
C5–C1–C2	107.99(12)
C5–C1–C6	126.42(13)
C2–C1–C6	125.50(13)
C5–C1–Al1	71.19(7)
C2–C1–Al1	71.28(7)
C6–C1–Al1	125.80(10)
C1–C2–C3	107.87(12)
C1–C2–C7	124.85(12)
C3–C2–C7	127.25(13)
C1–C2–Al1	71.19(8)
C3–C2–Al1	71.18(8)
C7–C2–Al1	124.85(10)
C4–C3–C2	107.97(13)
C4–C3–C8	124.66(13)
C2–C3–C8	127.37(15)
C4–C3–Al1	71.39(8)
C2–C3–Al1	71.26(8)
C8–C3–Al1	122.04(10)
C3–C4–C5	108.21(12)
C3–C4–C9	125.53(15)
C5–C4–C9	126.26(15)
C3–C4–Al1	71.19(8)
C5–C4–Al1	71.14(8)
C9–C4–Al1	123.98(10)
C4–C5–C1	107.96(13)
C4–C5–C10	126.00(14)
C1–C5–C10	126.04(14)
C4–C5–Al1	71.43(8)
C1–C5–Al1	71.27(8)
C10–C5–Al1	122.70(11)
C1–C6–H6A	109.5
C1–C6–H6B	109.5
H6A–C6–H6B	109.5
C1–C6–H6C	109.5
H6A–C6–H6C	109.5
H6B–C6–H6C	109.5
C2–C7–H7A	109.5
C2–C7–H7B	109.5
H7A–C7–H7B	109.5
C2–C7–H7C	109.5
H7A–C7–H7C	109.5
H7B–C7–H7C	109.5
C3–C8–H8A	109.5
C3–C8–H8B	109.5

H8A–C8–H8B	109.5
C3–C8–H8C	109.5
H8A–C8–H8C	109.5
H8B–C8–H8C	109.5
C4–C9–H9A	109.5
C4–C9–H9B	109.5
H9A–C9–H9B	109.5
C4–C9–H9C	109.5
H9A–C9–H9C	109.5
H9B–C9–H9C	109.5
C5–C10–H10A	109.5
C5–C10–H10B	109.5
H10A–C10–H10B	109.5
C5–C10–H10C	109.5
H10A–C10–H10C	109.5
H10B–C10–H10C	109.5
C12–C11–C15	108.25(13)
C12–C11–C16	125.27(17)
C15–C11–C16	126.47(18)
C12–C11–Al2	71.58(9)
C15–C11–Al2	71.35(8)
C16–C11–Al2	123.18(11)
C11–C12–C13	107.96(14)
C11–C12–C17	125.66(15)
C13–C12–C17	126.37(16)
C11–C12–Al2	71.15(9)
C13–C12–Al2	71.13(8)
C17–C12–Al2	124.30(12)
C14–C13–C12	107.84(13)
C14–C13–C18	125.06(13)
C12–C13–C18	127.06(14)
C14–C13–Al2	70.97(8)
C12–C13–Al2	71.38(8)
C18–C13–Al2	124.87(10)
C15–C14–C13	108.18(13)
C15–C14–C19	126.87(14)
C13–C14–C19	124.95(14)
C15–C14–Al2	71.56(8)
C13–C14–Al2	71.53(7)
C19–C14–Al2	122.19(10)
C14–C15–C11	107.77(14)
C14–C15–C20	126.21(16)
C11–C15–C20	126.03(15)
C14–C15–Al2	71.00(8)
C11–C15–Al2	71.15(8)
C20–C15–Al2	123.35(11)
C11–C16–H16A	109.5
C11–C16–H16B	109.5
H16A–C16–H16B	109.5
C11–C16–H16C	109.5
H16A–C16–H16C	109.5
H16B–C16–H16C	109.5
C12–C17–H17A	109.5
C12–C17–H17B	109.5
H17A–C17–H17B	109.5

C12–C17–H17C	109.5
H17A–C17–H17C	109.5
H17B–C17–H17C	109.5
C13–C18–H18A	109.5
C13–C18–H18B	109.5
H18A–C18–H18B	109.5
C13–C18–H18C	109.5
H18A–C18–H18C	109.5
H18B–C18–H18C	109.5
C14–C19–H19A	109.5
C14–C19–H19B	109.5
H19A–C19–H19B	109.5
C14–C19–H19C	109.5
H19A–C19–H19C	109.5
H19B–C19–H19C	109.5
C15–C20–H20A	109.5
C15–C20–H20B	109.5
H20A–C20–H20B	109.5
C15–C20–H20C	109.5
H20A–C20–H20C	109.5
H20B–C20–H20C	109.5
C26–C21–C22	115.70(11)
C26–C21–B1	121.82(11)
C22–C21–B1	122.03(11)
C23–C22–C21	121.88(12)
C23–C22–H22	119.1
C21–C22–H22	119.1
C22–C23–C24	121.20(12)
C22–C23–C27	120.98(12)
C24–C23–C27	117.81(11)
C25–C24–C23	117.84(12)
C25–C24–H24	121.1
C23–C24–H24	121.1
C24–C25–C26	121.01(12)
C24–C25–C28	120.90(12)
C26–C25–C28	118.08(12)
C25–C26–C21	122.32(12)
C25–C26–H26	118.8
C21–C26–H26	118.8
F3–C27–F1	107.10(12)
F3–C27–F2	105.57(11)
F1–C27–F2	105.22(11)
F3–C27–C23	113.52(11)
F1–C27–C23	112.87(11)
F2–C27–C23	111.94(11)
F4–C28–F6	107.02(12)
F4–C28–F5	106.17(13)
F6–C28–F5	106.05(12)
F4–C28–C25	112.19(12)
F6–C28–C25	113.06(12)
F5–C28–C25	111.88(11)
C34–C29–C30	115.67(11)
C34–C29–B1	122.16(11)
C30–C29–B1	121.79(11)
C31–C30–C29	122.45(12)
C31–C30–H30	118.8
C29–C30–H30	118.8
C30–C31–C32	120.88(12)
C30–C31–C35	119.20(12)
C32–C31–C35	119.86(12)
C33–C32–C31	117.74(12)
C33–C32–H32	121.1
C31–C32–H32	121.1
C32–C33–C34	121.05(12)
C32–C33–C36	120.07(12)
C34–C33–C36	118.87(12)
C33–C34–C29	122.13(12)
C33–C34–H34	118.9
C29–C34–H34	118.9
F9–C35–F8	107.24(11)
F9–C35–F7	105.93(12)
F8–C35–F7	105.32(12)
F9–C35–C31	112.94(12)
F8–C35–C31	112.91(11)
F7–C35–C31	111.94(11)
F11–C36–F10	106.95(11)
F11–C36–F12	105.90(12)
F10–C36–F12	106.03(11)
F11–C36–C33	112.17(12)
F10–C36–C33	113.14(12)
F12–C36–C33	112.15(11)
C21$^{\#2}$–B1–C21	112.65(14)
C21$^{\#2}$–B1–C29$^{\#2}$	112.70(6)
C21–B1–C29$^{\#2}$	103.56(6)
C21$^{\#2}$–B1–C29	103.56(6)
C21–B1–C29	112.70(6)
C29$^{\#2}$–B1–C29	111.96(14)

Symmetry transformations used to generate equivalent atoms:
#1: 0.5-X, +Y, 0.5-Z; #2: 1.5-X, +Y, 0.5-Z;

Table S22: Torsion angles for compound **5**.

**Atom–Atom–Atom–Atom**	**Torsion Angle [°]**
C5–C1–C2–C3	−0.01(15)
C6–C1–C2–C3	176.77(13)
Al1–C1–C2–C3	−62.10(9)
C5–C1–C2–C7	−178.01(13)
C6–C1–C2–C7	−1.2(2)
Al1–C1–C2–C7	119.90(13)
C5–C1–C2–Al1	62.09(10)
C6–C1–C2–Al1	−121.13(14)
C1–C2–C3–C4	−0.23(15)
C7–C2–C3–C4	177.71(13)
Al1–C2–C3–C4	−62.34(10)

C1–C2–C3–C8	178.58(14)
C7–C2–C3–C8	−3.5(2)
Al1–C2–C3–C8	116.47(15)
C1–C2–C3–Al1	62.11(9)
C7–C2–C3–Al1	−119.95(14)
C2–C3–C4–C5	0.38(16)
C8–C3–C4–C5	−178.46(13)
Al1–C3–C4–C5	−61.87(10)
C2–C3–C4–C9	−178.91(14)
C8–C3–C4–C9	2.2(2)
Al1–C3–C4–C9	118.84(15)
C2–C3–C4–Al1	62.25(9)
C8–C3–C4–Al1	−116.60(14)
C3–C4–C5–C1	−0.39(16)
C9–C4–C5–C1	178.90(14)
Al1–C4–C5–C1	−62.29(10)
C3–C4–C5–C10	179.31(14)
C9–C4–C5–C10	−1.4(2)
Al1–C4–C5–C10	117.41(15)
C3–C4–C5–Al1	61.90(10)
C9–C4–C5–Al1	−118.81(15)
C2–C1–C5–C4	0.25(16)
C6–C1–C5–C4	−176.50(14)
Al1–C1–C5–C4	62.40(10)
C2–C1–C5–C10	−179.45(14)
C6–C1–C5–C10	3.8(2)
Al1–C1–C5–C10	−117.30(15)
C2–C1–C5–Al1	−62.15(9)
C6–C1–C5–Al1	121.10(14)
C15–C11–C12–C13	0.32(16)
C16–C11–C12–C13	179.93(14)
Al2–C11–C12–C13	−61.97(10)
C15–C11–C12–C17	−178.49(15)
C16–C11–C12–C17	1.1(2)
Al2–C11–C12–C17	119.21(16)
C15–C11–C12–Al2	62.30(10)
C16–C11–C12–Al2	−118.10(15)
C11–C12–C13–C14	0.01(16)
C17–C12–C13–C14	178.82(15)
Al2–C12–C13–C14	−61.98(10)
C11–C12–C13–C18	−177.91(14)
C17–C12–C13–C18	0.9(2)
Al2–C12–C13–C18	120.10(15)
C11–C12–C13–Al2	61.98(10)
C17–C12–C13–Al2	−119.21(16)
C12–C13–C14–C15	−0.34(15)
C18–C13–C14–C15	177.64(13)
Al2–C13–C14–C15	−62.57(10)
C12–C13–C14–C19	179.10(13)
C18–C13–C14–C19	−2.9(2)
Al2–C13–C14–C19	116.87(14)
C12–C13–C14–Al2	62.24(10)
C18–C13–C14–Al2	−119.79(14)
C13–C14–C15–C11	0.53(16)
C19–C14–C15–C11	−178.89(14)

Al2–C14–C15–C11	−62.02(10)
C13–C14–C15–C20	−179.51(15)
C19–C14–C15–C20	1.1(2)
Al2–C14–C15–C20	117.94(16)
C13–C14–C15–Al2	62.55(10)
C19–C14–C15–Al2	−116.87(15)
C12–C11–C15–C14	−0.53(16)
C16–C11–C15–C14	179.87(14)
Al2–C11–C15–C14	61.92(10)
C12–C11–C15–C20	179.52(15)
C16–C11–C15–C20	−0.1(2)
Al2–C11–C15–C20	−118.03(16)
C12–C11–C15–Al2	−62.45(10)
C16–C11–C15–Al2	117.95(15)
C26–C21–C22–C23	0.86(17)
B1–C21–C22–C23	173.25(10)
C21–C22–C23–C24	1.08(19)
C21–C22–C23–C27	−177.52(11)
C22–C23–C24–C25	−1.72(18)
C27–C23–C24–C25	176.92(11)
C23–C24–C25–C26	0.42(18)
C23–C24–C25–C28	179.56(12)
C24–C25–C26–C21	1.60(19)
C28–C25–C26–C21	−177.57(11)
C22–C21–C26–C25	−2.18(17)
B1–C21–C26–C25	−174.59(10)
C22–C23–C27–F3	7.56(18)
C24–C23–C27–F3	−171.09(11)
C22–C23–C27–F1	−114.56(14)
C24–C23–C27–F1	66.80(16)
C22–C23–C27–F2	126.95(13)
C24–C23–C27–F2	−51.69(16)
C24–C25–C28–F4	131.33(14)
C26–C25–C28–F4	−49.50(17)
C24–C25–C28–F6	10.19(18)
C26–C25–C28–F6	−170.64(12)
C24–C25–C28–F5	−109.47(14)
C26–C25–C28–F5	69.70(16)
C34–C29–C30–C31	−2.66(18)
B1–C29–C30–C31	−175.81(10)
C29–C30–C31–C32	1.79(19)
C29–C30–C31–C35	−175.35(12)
C30–C31–C32–C33	0.75(18)
C35–C31–C32–C33	177.87(12)
C31–C32–C33–C34	−2.26(19)
C31–C32–C33–C36	176.74(12)
C32–C33–C34–C29	1.34(19)
C36–C33–C34–C29	−177.68(11)
C30–C29–C34–C33	1.11(18)
B1–C29–C34–C33	174.23(10)
C30–C31–C35–F9	−157.29(12)
C32–C31–C35–F9	25.53(18)
C30–C31–C35–F8	−35.40(17)
C32–C31–C35–F8	147.42(12)
C30–C31–C35–F7	83.25(15)

C32–C31–C35–F7	−93.93(15)
C32–C33–C36–F11	−119.05(14)
C34–C33–C36–F11	59.97(16)
C32–C33–C36–F10	2.04(18)
C34–C33–C36–F10	−178.93(11)
C32–C33–C36–F12	121.93(13)
C34–C33–C36–F12	−59.05(17)
C26–C21–B1–C21$^{\#1}$	−32.88(8)
C22–C21–B1–C21$^{\#1}$	155.18(12)
C26–C21–B1–C29$^{\#1}$	89.18(13)
C22–C21–B1–C29$^{\#1}$	−82.75(12)
C26–C21–B1–C29	−149.62(11)
C22–C21–B1–C29	38.44(15)
C34–C29–B1–C21$^{\#1}$	−83.80(12)
C30–C29–B1–C21$^{\#1}$	88.90(14)
C34–C29–B1–C21	38.23(15)
C30–C29–B1–C21	−149.07(12)
C34–C29–B1–C29$^{\#1}$	154.51(12)
C30–C29–B1–C29$^{\#1}$	−32.79(9)

Symmetry transformations used to generate equivalent atoms:
#1: 1.5-X, +Y, 0.5-Z;

5. Literature

(1) Smith, C. R.; Zhang, A.; Mans, D. J.; Rajanbabu, T. V.; Denmark, S. E.; Xie, M. (R)-3-METHYL-3-PHENYL-1-PENTENE VIA CATALYTIC ASYMMETRIC HYDROVINYLATION. *Org. Synth.* **2008**, *85*, 248-266.
(2) Useful Reagents and Ligands. In *Inorganic Syntheses*, *Inorg. Synth.*, 2002; pp 75-121.
(3) Yakelis, N. A.; Bergman, R. G. Safe Preparation and Purification of Sodium Tetrakis[(3,5-trifluoromethyl)phenyl]borate (NaBArF24): Reliable and Sensitive Analysis of Water in Solutions of Fluorinated Tetraarylborates. *Organometallics* **2005**, *24* (14), 3579-3581. DOI: 10.1021/om0501428.
(4) Tamm, M.; Randoll, S.; Herdtweck, E.; Kleigrewe, N.; Kehr, G.; Erker, G.; Rieger, B. Imidazolin-2-iminato titanium complexes: synthesis, structure and use in ethylene polymerization catalysis. *Dalton Tran.* **2006**, (3), 459-467.
(5) Liu, B.-C.; Ge, N.; Zhai, Y.-Q.; Zhang, T.; Ding, Y.-S.; Zheng, Y.-Z. An imido ligand significantly enhances the effective energy barrier of dysprosium(iii) single-molecule magnets. *Chem. Commun.* **2019**, *55* (63), 9355-9358.
(6) Franz, D.; Irran, E.; Inoue, S. Synthesis, characterization and reactivity of an imidazolin-2-iminato aluminium dihydride. *Dalton Trans.* **2014**, *43* (11), 4451-4461.
(7) Ganesamoorthy, C.; Loerke, S.; Gemel, C.; Jerabek, P.; Winter, M.; Frenking, G.; Fischer, R. A. Reductive elimination: a pathway to low-valent aluminium species. *Chem. Commun.* **2013**, *49* (28), 2858-2860.

Crystallography

[C1] *APEX4 Suite of Crystallographic Software, Version 2021-10.0,* Bruker AXS Inc., Madison, Wisconsin, USA, **2021**.
[C2] Bruker, *SAINT*, *V8.40B*, Bruker AXS Inc., Madison, Wisconsin, USA.
[C3] L. Krause, R. Herbst-Irmer, G. M. Sheldrick, D. Stalke, *J. Appl. Cryst.* **2015**, *48*, 3–10.
[C4] G. M. Sheldrick, *Acta Cryst.* **2015**, *A71*, 3–8.
[C5] G. M. Sheldrick, *Acta Cryst.* **2015**, *C71*, 3–8.
[C6] C. B. Huebschle, G. M. Sheldrick, B. Dittrich, *J. Appl. Cryst.* **2011**, *44*, 1281–1284.
[C7] D. Kratzert, J. J. Holstein, I. Krossing, *J. Appl. Cryst.*, **2015**, *48*, 933–938.
[C8] Ed. E. Prince, *International Tables for Crystallography Volume C, Mathematical, Physical and Chemical Tables*, International Union of Crystallography, Chester, England, **2006**, 500–502; 219–222; 193–199.
[C9] C. R. Groom, I. J. Bruno, M. P. Lightfoot, S. C. Ward, *Acta Cryst.* **2016**, *B72*, 171–179.
[C10] D. Kratzert, *FinalCif*, *V106*, https://dkratzert.de/finalcif.html.
[C11] C. F. Macrae, I. J. Bruno, J.A. Chisholm et al., *J. Appl. Cryst.*, **2008**, *41* (2), 466–470.
